# Viral etiology of acute respiratory infections in Sub-Saharan Africa during the pre-COVID-19 period (2006–2019): a systematic review and meta-analysis

**DOI:** 10.1186/s12879-025-12122-8

**Published:** 2025-11-23

**Authors:** Benjamin W. O. Kaboré, Nina Gouba, Abdoul Kader Ilboudo, Moussa Lingani, Madi Savadogo, Ezechiel Ouedraogo, Assana Cissé, Virginie Simonis, Zékiba Tarnagda

**Affiliations:** 1 Laboratoire National de Référence Grippes/Institut de Recherche en Sciences de la Santé (LNRG/IRSS), Ouagadougou, Burkina Faso; 2 UFR-SVT, Departement Biochimie-Microbiologie, University of Nazi Boni, Bobo-Dioulasso, Burkina Faso; 3 International Livestock Research Institute, Nairobi, Kenya; 4 Institut de Recherche en Sciences de la Santé/Direction Régionale du Centre Ouest (IRSS/DRCO), Nanoro, Burkina Faso; 5 Direction Générale des Services Vétérinaires, Ministère de l’Agriculture, des Ressources Animales et Halieutiques, Ouagadougou, Burkina Faso; 6 Fundamental and Applied Research for Animals and Health (FARAH), Faculty of Veterinary Medicine, University of Liege, Liege, Belgium; 7 Unité de Recherche Soins Primaires et Santé, Département de Médecine Générale, Université de Liege, Liege, Belgique

**Keywords:** Respiratory virus, Etiology, Prevalence, Acute respiratory infections, Sub-Saharan, Africa, Meta-analysis

## Abstract

**Objective:**

This systematic review and meta-analysis aimed to estimate the prevalence of respiratory viruses among people with acute respiratory infections (ARI) in Sub-Saharan Africa.

**Methods:**

We performed an electronic search through the PubMed, EMBASE, Medline, and Scopus databases to identify observational (cross-sectional and cohort), and randomized controlled trial studies published in English and French between January 2006, and December 2019. We used a random-effects meta-analysis to estimate the pooled prevalence of major respiratory viruses across studies. Heterogeneity (I^2^) was assessed via the chi-square test of Cochran’s Q statistic. A p-value < 0.05 was considered statistically.

**Results:**

This meta-analysis included 73 studies (199,393 participants). Human rhinovirus (HRV) was the most commonly detected virus at 21.2% (95% CI [16.76; 25.75]). The second predominant virus was respiratory syncytial virus (RSV) at 16% (95% CI [12.51; 19.59]), followed by human adenovirus (AdV) at 14.3% (95% CI [10.13; 18.57]), and influenza at 13.9% (95% CI [11.27; 16.62]). Other detected viruses included human parainfluenzavirus (HPIV) 8.9% (95% CI [6.08; 11.83]), human coronavirus (HCoV) 7.2% (95% CI [3.77; 10.67]), enterovirus (EV) 7% (95% CI [4.2; 9.81]), human metapneumovirus (HMPV) 4.6% (95% CI [3.53; 5.78]), and human bocavirus (HBoV) 4.1% (95% CI [1.99; 6.34]). Significant heterogeneity was observed across the overall prevalence and within subgroups for all viruses. Notable variations in respiratory virus prevalence were identified according to age, clinical presentation, setting, and Africa region.

**Conclusion:**

The present study has shown that HRV is the most common respiratory virus detected among ARI in Sub-Saharan Africa, followed by RSV, AdV, and influenza virus. Ongoing surveillance is important to monitor changes in the etiology, seasonality, and severity of pathogens of interest.

**Clinical trial number:**

Not applicable.

**Supplementary information:**

The online version contains supplementary material available at 10.1186/s12879-025-12122-8.

## Background

Worldwide, acute respiratory infections (ARIs) are one of the major causes of hospitalization, morbidity, and mortality rates [1]. In 2019, ARIs ranked as the fourth leading cause of death globally, responsible for approximately three million deaths according to the World Health Organization (WHO) [2]. Viruses, which are the primary contributors to ARIs, followed by bacteria and fungi, are responsible for a lesser portion of the global ARI-related mortality [3]. Commonly detected viruses include influenza virus, respiratory syncytial virus (RSV), human rhinovirus (HRV), enterovirus (EV), human parainfluenza virus (HPIV), and adenoviruses (AdV) [4, 5]. The transmission and prevalence of these viruses are influenced by geographic, demographic, socioeconomic, and environmental factors [6, 7]. These factors contribute to the variability in ARI impact across different regions and populations.

As the burden of ARIs increases globally, advances in molecular biology have significantly improved the sensitivity of virus detection in respiratory diseases. These advancements have made it possible to detect additional viruses such as human metapneumoviruses (HMPV) [8], Severe acute respiratory syndrome-associated coronavirus (SARS-CoV) [9], Middle East respiratory syndrome coronavirus (MERS-CoV) [10], and human bocaviruses (HBoV) [11], as well as the more recently SARS-CoV-2 [12]. These developments in diagnostic technologies enhance detection and help reduce unnecessary antibiotic use, addressing the growing concern of antimicrobial resistance. This highlights the increasing complexity of ARI etiology and underscores the need for accurate diagnosis to guide appropriate treatment.

However, understanding viral etiology alone does not directly guide treatment. The absence of specific clinical symptoms for each pathogen, combined with the possibility of mixed infections, complicates diagnosis. Accurate identification of the causative agent is essential for optimizing therapeutic management and making informed decisions about the initiation or continuation of antibiotic treatment.

Despite global advances in diagnostics, many regions, particularly in Sub-Saharan Africa, face significant barriers in diagnosing and managing ARIs due to limited healthcare resources, awareness, and access to technology. These limitations hinder effective disease management and result in the unique epidemiology of ARIs within the region. The available information is often sparse and fragmented, leading to a lack of comprehensive data on the burden of viral infections. A deeper understanding of the epidemiological landscape is crucial for developing effective public health policies aimed at controlling ARIs. However, there is currently no systematic review examining the viral etiology associated with ARIs in Sub-Saharan Africa. Addressing this gap, our systematic review and meta-analysis aim to estimate the prevalence of respiratory viral infections among individuals with ARIs in the region from 2006 to 2019.

## Methods

### Study design and ethical considerations

This systematic review was conducted according to the updated **PRISMA** guidelines (Table S1 in S1 File) [13]. This review reports previously published data and ethical clearance was not required.

### Search strategy

A systematic literature review was performed using the search terms detailed in the Supplementary online material. This was supplemented by hand searching of key online journals and reference lists of selected papers. The search included the following databases: PubMed, Medline, Embase, and Scopus. We included studies conducted in Sub-Saharan Africa and published between January 2006 to December 2019. The search strategy conducted in this study is shown in Table S2 in S1 File.

### Integration criteria

Observational studies (cross-sectional, cohort), and randomized controlled trial studies were considered if they reported the detection of respiratory viruses using polymerase chain reaction (PCR) assay on respiratory samples. Case reports, reviews, and studies reporting chronic respiratory infections were excluded.

### Data selection

Two investigators independently screened records based on titles and abstracts for eligibility. Full texts of articles deemed potentially eligible were retrieved. These investigators independently assessed the full text of each study for eligibility and consensually selected studies for inclusion. Any disagreements were solved.

Data from included studies were extracted using standardized templates formed by two investigators independently. The information gathered included: the name of the first author, the year of publication, the design of study, the country and setting, the period of study, the sample type, the viral detection assay, age range, the clinical presentation, the number of samples tested, the number of positives for each virus and the data of evaluation of study quality. We assigned the Sub-Saharan African region (Central, Eastern, Western, and Southern) to each study regarding the country of recruitment [14]. Additionally, we categorized clinical presentations into two groups: severe respiratory tract infections (SRTI), including severe acute respiratory infection, acute lower respiratory infections, bronchitis, bronchiolitis, pneumonia, and severe or very severe pneumonia; and benign respiratory tract infections (BRTI), which included upper respiratory tract infections and influenza-like illness. We assessed whether the case definitions used to include study participants aligned with those of the WHO [15]. Disagreements between investigators were reconciled through discussion and consensus or an arbitration of a third investigator.

### Assessing research quality

Two investigators evaluated the risk of bias in the included studies using a Hoy and al [16] modified eight-item rating scale (Table S3 in S1 File). The risk of bias in the included studies was estimated as low (6–8), moderate (3–5), and high risk (0–2). Disagreements were solved through discussion and consensus.

### Data synthesis and analysis

Statistical analysis was performed with R Core Team software (version 3.3.3.) with the “meta” and “metafor” packages. In all models, meta-analysis was performed using the random-effects regression approach [17]. We used “metaprop” to estimate the pooled effects of the prevalence. Unadjusted prevalence was recalculated based on the information of crude numerators (case number) and denominators (sample size) provided by individual studies for each virus. A dual arcsine transformation of Freeman-Tukey was used to stabilize the variances in the prevalence calculation. This procedure stabilized the variance of study-specific prevalence before applying a random-effects model to assess heterogeneity and generate a pooled prevalence estimate. Studies were pooled using the inverse and logit-transformation approaches to estimate the pooled prevalence with Clopper-Pearson 95% confidence intervals (CI). Heterogeneity was assessed using the chi-square test on Cochrane’s Q statistic [18], and quantified by H and I^2^ values. The I^2^ statistic represents the percentage of total variation across studies due to true between-study differences rather than chance. Generally, I^2^ values greater than 70% indicate substantial heterogeneity. Value of H close to 1 suggest some homogeneity between studies [18]. Forest plots, summary tables, and a narrative summary were used to present overall results. Publication bias was evaluated through visual inspection of funnel plots and the Egger test [19]. Subgroup analyses were conducted for variables of public health importance including age groups, clinical presentations, locality, setting, and African regions. Heterogeneity in the subgroups was assessed using the same methods described. Statistical significance was considered at p-value < 0.05.

## Results

### Review process

**Fig. 1:**
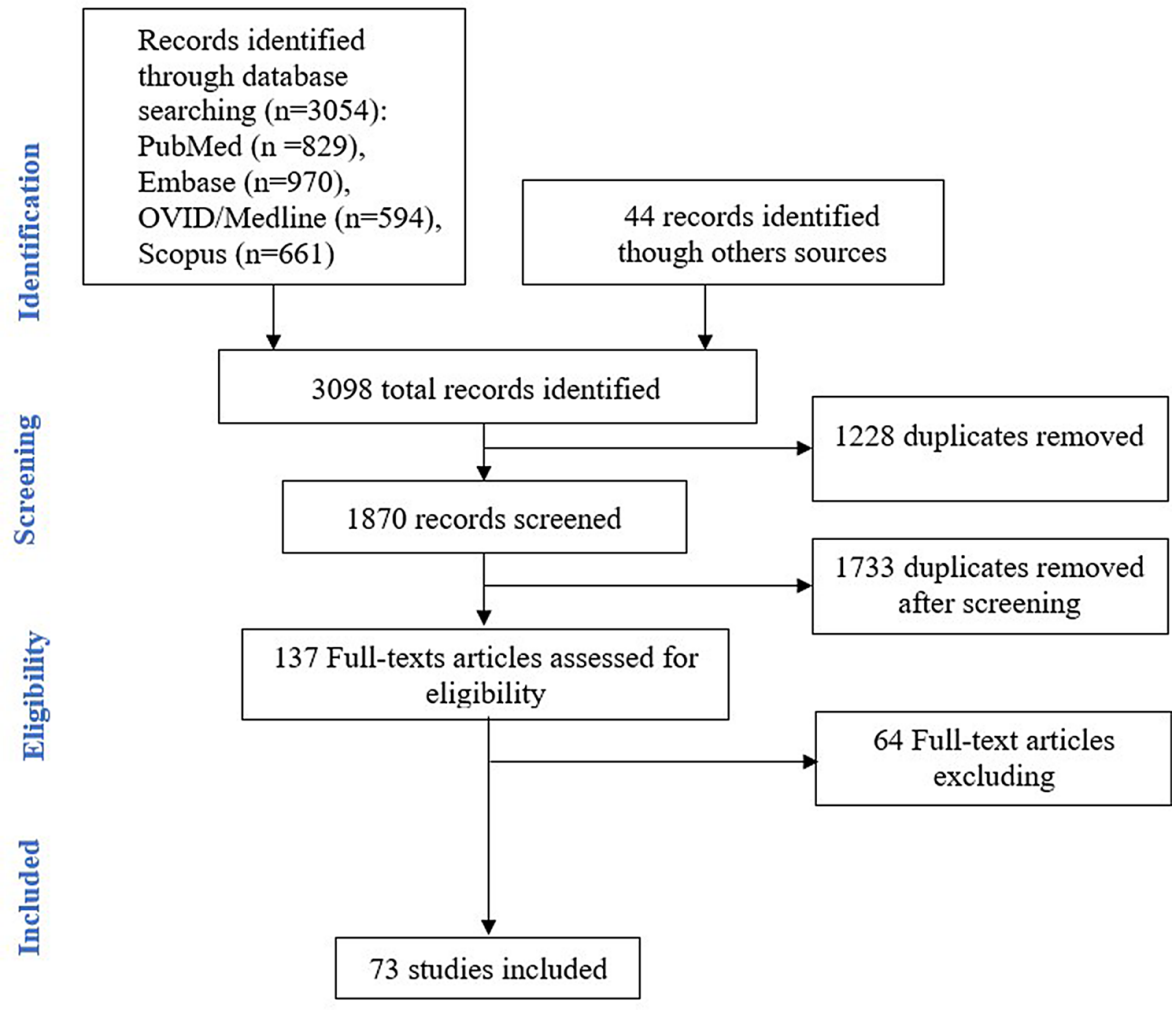
PRISMA flow diagram of the literature search

The literature search provided a total of 3098 records. After removing duplicates, 1870 records remained. After the screening of titles and abstracts, we excluded 1733 irrelevant records. We assessed the full texts of the remaining 137 papers for eligibility, of which 64 were excluded with reasons (Fig. 1). A total of 73 studies that met our strict eligibility criteria were included [20–93]. The selected studies were published between 2008 and 2019 (Table S4 in S1 File).

### Characteristics of included studies

Data were collected from 24 countries (involving 199,393 participants). Most of the studies were cross-sectional with a prospective design. Many of these studies were conducted in urban settings, with a notable concentration in Western Africa. Specifically, 53.4% of the studies primarily focused on children. Most studies adhered to the WHO case definition criteria (83.6%) for included participants. The most prevalent clinical presentation among participants was SRTI (48%). Analyzed samples were mainly nasopharyngeal secretions (63.3%). Most studies were at low risk of bias (93.1%) (Table 1).

**Table 1:** Sociodemographic and clinical characteristics of included studies

Characteristics	Data (%)
Design, n (%)	
Cross-sectional	69 (94.6%)
Cohort	2 (2.7%)
Randomized controlled trial	2 (2.7%
Setting, n (%)	
Urban	31 (42.5%)
Rural	10 (13.7%)
Urban and rural	23 (31.5%)
Not described	9 (12.3%)
Timing, n (%)	
Prospective	65 (89%)
Retrospective	8 (11%)
Study bias, n (%)	
Low risk of bias	68 (93.1%)
Moderate risk of bias	5 (6.9%)
Clinical presentation, n (%)	
SRTI	35 (48%)
BRTI	16 (21.9%)
SRTI and BRTI	22 (30.1%)
WHO definition criteria, n (%)	
YES	61 (83.6%)
NO	12 (16.4%)
Population, n (%)	
Children (under 15 years)	39 (53.4%)
Adults	3 (4%)
Children and Adults	31 (42.6%)
Africa Region, n (%)	
Eastern (Ethiopia = 1; Kenya = 11; Madagascar = 3; Rwanda = 1; Tanzania = 1; Uganda = 2; Sudan = 1; Malawi = 2)	22 (30.1%)
Western (Burkina Faso = 4; Ghana = 4; Republic of Cote d Ivoire = 1; Gambia = 2; Niger = 2; Nigeria = 3; Senegal = 7; Togo = 1)	24 (32.9%)
Southern (South Africa = 14; Mozambique = 3; Zambia = 2)	19 (26%)
Central (Cameroon = 3; Central Africa Republic = 2; Democratic Republic of Congo = 2; Gabon = 1)	8 (11.0%)
Respiratory Specimens, n (%)	100%
Nasopharyngeal: 63.37% Nasopharyngeal and Oropharyngeal: 21.78% Throat and Nasal: 1.98% Nasopharyngeal/Tracheal/Broncho-alveolar lavage: 1.98% Nasal: 5.94% Not described: 1.98% Oropharyngeal: 2.97%	
Diagnostic technique, n (%)	
Real-Time RT-PCR	73 (100%)

Abbreviations: BRTI-Benign respiratory tract infections; SRTI-Severe respiratory tract infections; WHO-World Health Organisation; RT-PCR-Real-Time polymerase chain reaction

### Prevalence of respiratory virus infections among ARI in Sub-Saharan Africa

The analysis showed that HRV had a higher pooled prevalence of virus detected (21.2%; 95% CI [16.76; 25.75]). Other viruses included in descending order: RSV (16%; 95% CI [12.51; 19.59], AdV (14.3%; 95% CI [10.13; 18.57], influenza virus (13.9%; 95% CI [11.27;16.62]), HPIV (8.9%; 95% CI [6.08; 11.83], HCoV (7.2%; 95% CI [3.77; 10.67], EV (7%; 95% CI [4.20; 9.81]), HMPV (4.6%, 95% CI [3.53; 5.78]) and HBoV (4.1%; 95% CI [1.99; 6.34]) (Table S5 in S1 File). The analysis showed substantial heterogeneity in overall pooled prevalence and within subgroups for all viruses (Fig. S1 in S1 File; Figs. S1-S9 in S2 File). Publication bias was detected for influenza virus, RSV, HRV, and AdV (Fig.S2 in S1 File).

### Subgroup analysis

The subgroup analysis revealed several important findings regarding the pooled prevalence of respiratory viruses (Table S5 in S1 File). The analysis found no statistically significant correlation between pooled prevalence and age group, except for influenza virus (*p* = 0.0022) and RSV (*p* < 0.0001) with children under 15 years old compared to adults.

Furthermore, within the pediatric population, individuals under 5 years old exhibited higher pooled prevalence rates compared to those over 5 years old for most viruses, including influenza virus (*p* = 0.0219), RSV (*p* < 0.0001), HMPV (*p* = 0.0350), HPIV (*p* = 0.0268), EV (*p* = 0.0006), and AdV (*p* = 0.0147). Subgroup analyses also revealed regional differences in the distribution of viruses within Sub-Saharan Africa. Specifically, substantial differences were observed for the influenza virus (*p* < 0.001), HRV (*p* = 0.005), and HCoV (*p* = 0.002). Additionally, regional differences in pooled prevalence were evident. In rural areas, lower pooled prevalence rates were observed for RSV (*p* = 0.006), EV (*p* = 0.024), and HBoV (*p* = 0.001) compared to urban areas.

However, a higher pooled prevalence of HMPV was found in rural settings (*p* = 0.007). Regarding the subgroup analyses according to the severity of respiratory infections; the analysis showed a significant increase in pooled prevalence in patients with severe respiratory tract infections (SRTI) compared to those with less severe bronchial respiratory tract infections (BRTI) for RSV (*p* = 0.01), HRV (*p* = 0.003), and HBoV (*p* = 0.006).

Conversely, a lower pooled prevalence of influenza virus was found in SRTI patients compared to patients with BRTI (*p* = 0.044). Our study revealed significant sources of heterogeneity in the estimation of pooled prevalence of all respiratory viruses in the subgroup analysis (Table S5 in S1 File; Fig. S1-S9 in S2 File).

## Discussion

This systematic review and meta-analysis, incorporating data from 73 studies involving 199,393 participants, provides a comprehensive assessment of the pooled prevalence of respiratory viruses among individuals with ARI in Sub-Saharan Africa. There was substantial heterogeneity across studies, reflecting variations in settings, populations, and methodologies.

### Pooled prevalence of respiratory viruses

Our study found that HRV had the highest pooled prevalence at 21.2%, similar to previous reports of 22.1% among community-acquired pneumonia patients [94]. This corroborates our observation that HRV infection was the most prevalent virus in Sub-Saharan Africa [94–96]. RSV was the second most prevalent virus, and our findings align with other studies [94, 97–99]. The overall prevalence of AdV was 14.3%, consistent with reports from the United States (15%) [100] and East Africa (13%) [101], but higher than in the Eastern Mediterranean (9.8%) [102]. Influenza virus prevalence was 13%, compared to regions like Southeast Asia (11%) [103] and the Middle East (10.2%) [104], with regional differences likely due to seasonality and vaccination efforts [105]. HPIV had an overall prevalence of 8.9%, consistent with East Africa (9%) [102] but higher than Latin America (3.2%) [106].

Finally, we found higher prevalence rates for HCoV, EV, HMPV, and HBoV compared to those reported in a previous systematic review [107], which may be due to differences in study design or regional virus circulation, highlighting the need for context-specific data.

### Comparison of RSV and influenza virus prevalence

Our findings indicated a significantly higher overall prevalence of RSV in comparison to influenza viruses. This disparity may be attributed to various factors, including differences in transmission dynamics that allow RSV to spread more effectively in certain environments [108, 109], seasonal variations that favor RSV activity during specific times of the year [110], and the immune responses present in the population that may offer less protection against RSV [111]. Additionally, the significant impact of RSV on young children, often leading to severe respiratory illnesses, may result in its more frequent detection in clinical settings, thereby contributing to its elevated prevalence [108].

### Factors contributing to high prevalence

The high prevalence of respiratory viruses in Sub-Saharan Africa can be attributed to several factors: subtropical climate; clinical diagnosis and sampling methods, and healthcare Access [112, 113]. Our study’s focus on sensitive PCR assays provides clearer insights. Geographic and demographic differences contribute to significant variations in infection rates [114]. Studies reporting higher rates may be more likely to be published, distorting the overall understanding of the viral landscape.

### Age-related findings

The subgroup analysis revealed a significant difference in the prevalence of certain respiratory viruses based on age. In our study, we found higher prevalence in children under 15 years compared to adults for influenza virus and RSV. This finding is consistent with existing literature, which suggests that these infections are more predominant in younger populations [106, 115, 116]. Within the pediatric population, children under 5 years exhibited higher prevalence rates for multiple viruses, including influenza, RSV, HMPV, HPIV, EV, and AdV. This finding underscores the heightened vulnerability of younger children to these infections [117], which could be attributed to their less developed immune systems and higher exposure rates in settings like schools and daycares [101]. The higher prevalence of RSV and HRV in children aligns with established knowledge about the increased susceptibility of younger age groups to these viruses. RSV is particularly known for being an important etiological agent of ARIs and a source of severe respiratory illness in infants and young children. Similarly, HRV has been implicated in a range of respiratory illnesses in young children, including asthma exacerbations [118, 119]. Younger age, particularly in children under five—was also associated with very severe cases and prolonged hospitalization [117], further reinforcing their increased vulnerability due to immature immune systems and higher exposure risks [119].

### Regional and locality variations

The analysis also identified significant regional differences in virus distribution across Sub-Saharan Africa, reflecting findings from previous studies [45, 94]. For instance, substantial differences were observed for the influenza virus, HRV, and HCoV. This regional variability may be attributed to factors such as socioeconomic conditions, healthcare access, and environmental influences that can affect viral transmission dynamics [112, 120, 121]. Notably, the prevalence of RSV, EV, and HBoV was lower in rural areas compared to urban settings, which aligns with these studies’ findings [122, 123]. Conversely, a higher prevalence of HMPV was found in rural regions. These findings suggest that urbanization may influence the spread of certain viruses, likely due to higher population density and increased human interactions, which facilitate transmission [124].

### Severity of infections

The severity analysis revealed a significant increase in prevalence among patients with SRTI compared to those with less BRTI for RSV, HRV, and HBoV. This aligns with previous research indicating that RSV is a leading cause of hospitalization in young children due to severe respiratory infections [125, 126]. RSV infections are known to be a leading cause of morbidity, mortality, and hospitalization among children [127]. The high prevalence of these viruses observed in SRTI suggests a potential role of these viruses in the severity of respiratory infections [126, 128, 129]. Interestingly, a lower prevalence of influenza virus was found in SRTI patients compared to those with BRTI. This may indicate that other viruses could be responsible for severe acute respiratory infections, making the role of the influenza virus less significant in these cases [130].

This study underscores the importance of understanding the demographic and regional variations in the prevalence of respiratory viruses. These findings can inform targeted public health interventions, including vaccination strategies and healthcare resource allocation, particularly in high-risk groups. Further research is necessary to explore the underlying factors contributing to these observed differences.

### Limitations

This study has several limitations. First, the lack of meta-regression limited our ability to analyze sources of heterogeneity related to study design and population characteristics. Second, we excluded systematic reviews that included studies not meeting our inclusion criteria, which may have introduced selection bias. Third, some included studies were conducted during the 2009 H1N1 influenza pandemic, a period that may have influenced virus prevalence due to enhanced surveillance and changes in clinical management. Furthermore, the restriction to studies published in English and French may have excluded relevant data from Portuguese- and Spanish-speaking countries, potentially limiting regional representativeness.

These limitations should be considered when interpreting the results, and future studies should strive for more standardized data collection, broader geographic coverage, and inclusive language criteria.

## Conclusions

This systematic review and meta-analysis provides a comprehensive synthesis of the viral etiology of ARI in Sub-Saharan Africa from 2006 to 2019. HRV and RSV were the most prevalent viruses identified, with notable variation by age, region, and clinical severity. Children under five years were disproportionately affected, highlighting the need for age-specific interventions. The findings emphasize the importance of strengthening regional surveillance systems, improving diagnostic capacity, and implementing targeted public health strategies. Future research should focus on addressing current limitations by adopting standardized methodologies, conducting meta-regression analyses, and expanding data collection to underrepresented regions and language groups. These efforts are essential to refine prevalence estimates and support effective respiratory infection control in Sub-Saharan Africa.

## supplementary material

Below is the link to the electronic supplementary material.

Supplementary Material 1

Supplementary Material 2

## Data Availability

This published article and its supplementary information files include all data generated or analyzed during this study.
