# Supplementary material for: Viral etiology of acute respiratory infections in Sub-Saharan Africa during the pre-COVID-19 period (2006–2019): a systematic review and meta-analysis
Source: BMC Infect Dis. 2025 Nov 23;25:1799. doi: 10.1186/s12879-025-12122-8 (PMC12750592; doi:10.1186/s12879-025-12122-8)

Fig. S1 Forest plots for respiratory viruses among people with ARI in Sub-Saharan africa

Influenza (57 studies)

Study	Events	Total	Proportion	95%-CI	Weight
Dia, 2014 (2)	67	232	0.29	[0.23; 0.35]	1.6%
Mohamed, 2015	66	419	0.16	[0.12; 0.20]	1.7%
Ho, 2018	163	1126	0.14	[0.12; 0.17]	1.8%
Cohen, 2015 (2)	621	7052	0.09	[0.08; 0.09]	1.8%
Hoffman, 2012	20	295	0.07	[0.04; 0.10]	1.8%
O'Callaghan-Gordo, 2011 (2)	51	333	0.15	[0.12; 0.20]	1.7%
Mackenzie, 2019	200	532	0.38	[0.33; 0.42]	1.7%
O'Callaghan-Gordo, 2011 (1)	39	475	0.08	[0.06; 0.11]	1.8%
Akinloye, 2011	17	246	0.07	[0.04; 0.11]	1.7%
Ghani, 2012	26	175	0.15	[0.10; 0.21]	1.7%
Kabego, 2018	1	146	0.01	[0.00; 0.04]	1.8%
Sanou, 2018	124	924	0.13	[0.11; 0.16]	1.8%
Famoroti, 2018	73	2172	0.03	[0.03; 0.04]	1.8%
Johnson, 2008	19	419	0.05	[0.03; 0.07]	1.8%
Nunes, 2014	61	1460	0.04	[0.03; 0.05]	1.8%
Ouedraogo, 2014	13	153	0.08	[0.05; 0.14]	1.7%
Smuts, 2008	9	1055	0.01	[0.00; 0.02]	1.8%
Pale, 2017	11	450	0.02	[0.01; 0.04]	1.8%
Serengbe, 2015	58	361	0.16	[0.12; 0.20]	1.7%
Cohen, 2016	56	1410	0.04	[0.03; 0.05]	1.8%
Lagare, 2015	40	160	0.25	[0.19; 0.32]	1.6%
Kwofie, 2012	1	128	0.01	[0.00; 0.04]	1.8%
Mazur, 2017	30	2322	0.01	[0.01; 0.02]	1.8%
Maman, 2014	236	955	0.25	[0.22; 0.28]	1.8%
Feikin, 2012	319	1216	0.26	[0.24; 0.29]	1.8%
Emukule, 2014	348	5898	0.06	[0.05; 0.07]	1.8%
Feikin, 2013	90	204	0.44	[0.37; 0.51]	1.6%
Nakouné, 2013	28	329	0.09	[0.06; 0.12]	1.7%
Onyango, 2012	71	1404	0.05	[0.04; 0.06]	1.8%
Enan, 2013	66	334	0.20	[0.16; 0.24]	1.7%
Jallow, 2019	19	8209	0.00	[0.00; 0.00]	1.8%
Fall, 2016	83	5338	0.02	[0.01; 0.02]	1.8%
Lonngren, 2014	39	569	0.07	[0.05; 0.09]	1.8%
Kim, 2011	321	2331	0.14	[0.12; 0.15]	1.8%
Njouom, 2010	70	238	0.29	[0.24; 0.36]	1.7%
Razanajatovo, 2011	97	313	0.31	[0.26; 0.36]	1.7%
Balinandi, 2013	119	369	0.32	[0.28; 0.37]	1.7%
Niang, 2012	1233	9176	0.13	[0.13; 0.14]	1.8%
Wabwire-Mangen, 2016	687	6628	0.10	[0.10; 0.11]	1.8%
Maïnassara, 2015	200	2128	0.09	[0.08; 0.11]	1.8%
Woyessa, 2018	988	4799	0.21	[0.19; 0.22]	1.8%
Theo, 2012	57	1234	0.05	[0.04; 0.06]	1.8%
Tamfum, 2012	605	4156	0.15	[0.13; 0.16]	1.8%
Kenmoe, 2016	34	347	0.10	[0.07; 0.13]	1.7%
Peterson, 2016	242	1505	0.16	[0.14; 0.18]	1.8%
Razanajatovo, 2018	222	876	0.25	[0.22; 0.28]	1.8%
Tarnagda, 2014	58	881	0.07	[0.05; 0.08]	1.8%
Njouom, 2012	158	561	0.28	[0.24; 0.32]	1.7%
Dia, 2014 (1)	317	1427	0.22	[0.20; 0.24]	1.8%
Kadjo, 2012	969	5074	0.19	[0.18; 0.20]	1.8%
Nyatanyi, 2012	377	1916	0.20	[0.18; 0.22]	1.8%
Ahmed, 2012	768	6264	0.12	[0.11; 0.13]	1.8%
Katz, 2014	6674	38775	0.17	[0.17; 0.18]	1.8%
Mmbaga,2012	140	1778	0.08	[0.07; 0.09]	1.8%
Pretorious, 2016	688	5743	0.12	[0.11; 0.13]	1.8%
Pretorious, 2012	703	8173	0.09	[0.08; 0.09]	1.8%
Thiam, 2015	847	2419	0.35	[0.33; 0.37]	1.8%
**Random effects model**	**19639**	**153612**	**0.14**	**[0.11; 0.17]**	**100.0%**

Heterogeneity: $I^2 = 100\%$, $\tau^2 = 0.0104$, $p = 0$

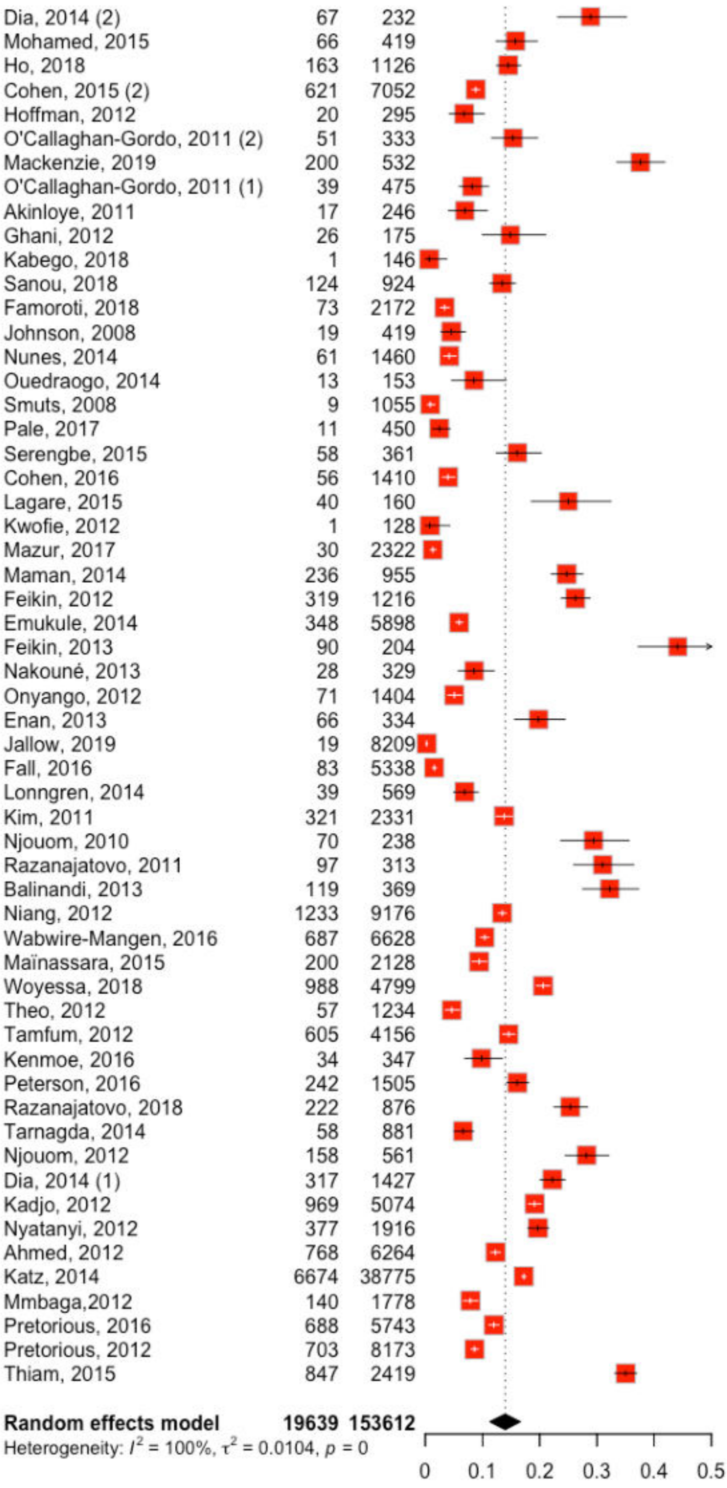

RSV (53 studies)

Study	Events	Total	Graphical proportions	Proportion	95%-CI	Weight
Dia, 2014 (2)	7	232		0.03	[0.01; 0.06]	1.9%
Mohamed, 2015	16	419		0.04	[0.02; 0.06]	1.9%
Moyes, 2017	329	7796		0.04	[0.04; 0.05]	1.9%
Cohen, 2015 (2)	321	7052		0.05	[0.04; 0.05]	1.9%
Nyawanda, 2016	470	3810		0.12	[0.11; 0.13]	1.9%
Hoffman, 2012	24	295		0.08	[0.05; 0.12]	1.9%
O'Callaghan-Gordo, 2011 (1)	50	475		0.11	[0.08; 0.14]	1.9%
Akinloye, 2011	1	246		0.00	[0.00; 0.02]	1.9%
Ghani, 2012	54	175		0.31	[0.24; 0.38]	1.8%
Simusika, 2015	24	297		0.08	[0.05; 0.12]	1.9%
Kabego, 2018	31	146		0.21	[0.15; 0.29]	1.8%
Adiku, 2015	20	108		0.19	[0.12; 0.27]	1.8%
Obodai, 2014	32	53		0.60	[0.46; 0.74]	1.5%
Famoroti, 2018	316	2172		0.15	[0.13; 0.16]	1.9%
Johnson, 2008	28	419		0.07	[0.04; 0.10]	1.9%
Nunes, 2014	237	1460		0.16	[0.14; 0.18]	1.9%
Ouédraogo Yugbaré, 2016	24	208		0.12	[0.08; 0.17]	1.9%
Ouedraogo, 2014	24	153		0.16	[0.10; 0.22]	1.8%
Oladele, 2019	41	120		0.34	[0.26; 0.43]	1.7%
Obodai, 2018	127	552		0.23	[0.20; 0.27]	1.9%
Smuts, 2008	42	1055		0.04	[0.03; 0.05]	1.9%
Pale, 2017	113	450		0.25	[0.21; 0.29]	1.9%
Serengbe, 2015	21	361		0.06	[0.04; 0.09]	1.9%
Cohen, 2016	469	1410		0.33	[0.31; 0.36]	1.9%
Moyes, 2013	1157	4293		0.27	[0.26; 0.28]	1.9%
Lagare, 2015	51	160		0.32	[0.25; 0.40]	1.8%
Kwofie, 2012	18	128		0.14	[0.09; 0.21]	1.8%
Agoti, 2014	240	834		0.29	[0.26; 0.32]	1.9%
Cohen, 2015 (1)	2268	8723		0.26	[0.25; 0.27]	1.9%
Howie, 2014	2	55		0.04	[0.00; 0.13]	1.9%
Mazur, 2017	1330	2322		0.57	[0.55; 0.59]	1.9%
Feikin, 2012	92	1216		0.08	[0.06; 0.09]	1.9%
Emukule, 2014	437	5898		0.07	[0.07; 0.08]	1.9%
Feikin, 2013	17	204		0.08	[0.05; 0.13]	1.9%
Nakouné, 2013	10	329		0.03	[0.01; 0.06]	1.9%
Enan, 2013	27	334		0.08	[0.05; 0.12]	1.9%
Jallow, 2019	28	8209		0.00	[0.00; 0.00]	1.9%
Fall, 2016	316	5338		0.06	[0.05; 0.07]	1.9%
Lonngren, 2014	244	569		0.43	[0.39; 0.47]	1.9%
Kim, 2011	328	2331		0.14	[0.13; 0.16]	1.9%
Razanajatovo, 2011	65	313		0.21	[0.16; 0.26]	1.9%
Balinandi, 2013	124	369		0.34	[0.29; 0.39]	1.9%
Niang, 2012	266	9176		0.03	[0.03; 0.03]	1.9%
Kenmoe, 2016	46	347		0.13	[0.10; 0.17]	1.9%
Peterson, 2016	164	1505		0.11	[0.09; 0.13]	1.9%
Razanajatovo, 2018	348	876		0.40	[0.36; 0.43]	1.9%
Lekana-Douki, 2014	119	1041		0.11	[0.10; 0.14]	1.9%
Njouom, 2012	32	561		0.06	[0.04; 0.08]	1.9%
Dia, 2014 (1)	158	1427		0.11	[0.09; 0.13]	1.9%
Ahmed, 2012	781	6264		0.12	[0.12; 0.13]	1.9%
Pretorious, 2016	616	5743		0.11	[0.10; 0.12]	1.9%
Pretorious, 2012	1169	8173		0.14	[0.14; 0.15]	1.9%
Pretorious, 2013	2182	13432		0.16	[0.16; 0.17]	1.9%
**Random effects model**	**15456**	**119634**		**0.16**	**[0.13; 0.20]**	**100.0%**

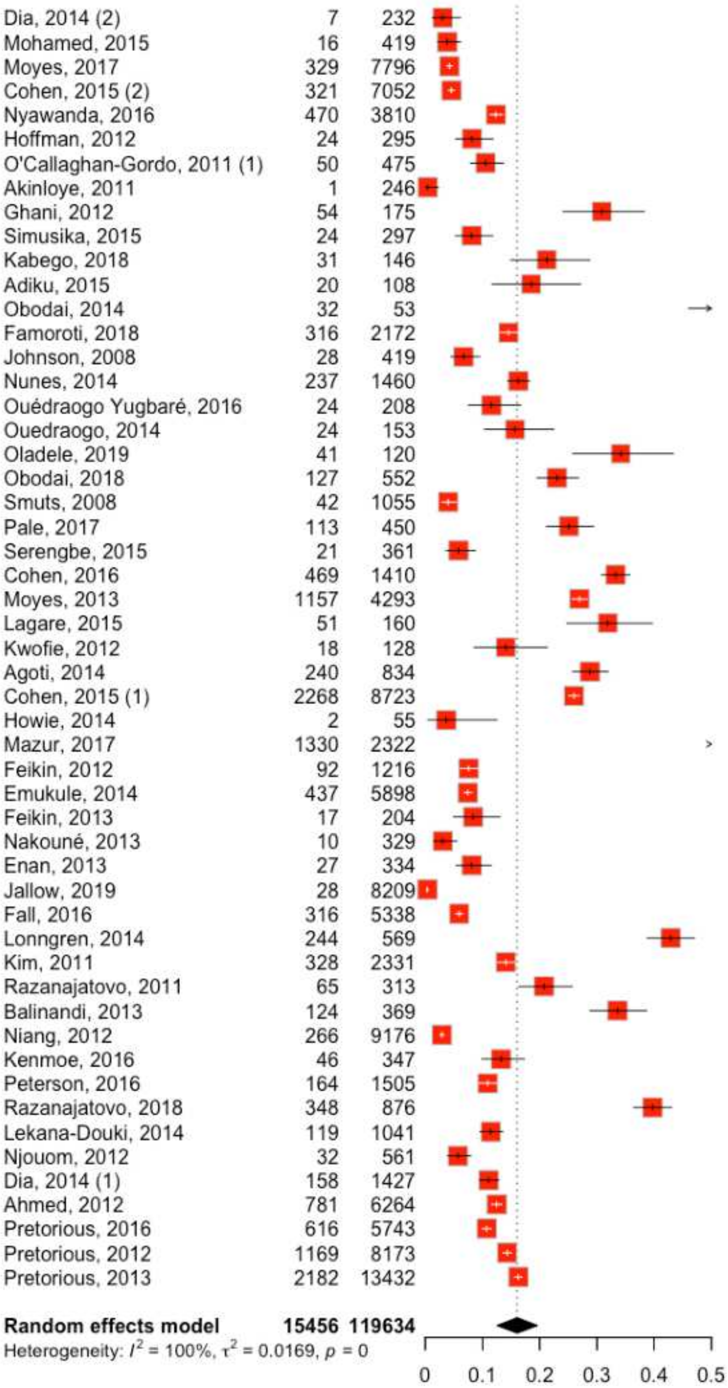

Heterogeneity: $I^2 = 100\%$, $\tau^2 = 0.0169$, $p = 0$

HMPV (29 studies)

Study	Events	Total	Proportion	95%-CI	Weight
Mohamed, 2015	14	419	0.03	[0.02; 0.06]	3.5%
Cohen, 2015 (2)	124	7052	0.02	[0.01; 0.02]	3.8%
Hoffman, 2012	31	295	0.11	[0.07; 0.15]	2.8%
O'Callaghan-Gordo, 2011 (2)	22	333	0.07	[0.04; 0.10]	3.1%
O'Callaghan-Gordo, 2011 (1)	39	475	0.08	[0.06; 0.11]	3.2%
Akinloye, 2011	7	246	0.03	[0.01; 0.06]	3.4%
Ghani, 2012	12	175	0.07	[0.04; 0.12]	2.7%
Simusika, 2015	11	297	0.04	[0.02; 0.07]	3.3%
Famoroti, 2018	14	2172	0.01	[0.00; 0.01]	3.8%
Nunes, 2014	107	1460	0.07	[0.06; 0.09]	3.6%
Ouedraogo, 2014	1	153	0.01	[0.00; 0.04]	3.6%
Smuts, 2008	28	1055	0.03	[0.02; 0.04]	3.7%
Owor, 2016	160	3320	0.05	[0.04; 0.06]	3.8%
Cohen, 2016	72	1410	0.05	[0.04; 0.06]	3.7%
Lagare, 2015	40	160	0.25	[0.19; 0.32]	1.6%
Feikin, 2012	60	1216	0.05	[0.04; 0.06]	3.7%
Feikin, 2013	15	204	0.07	[0.04; 0.12]	2.7%
Fall, 2016	60	5338	0.01	[0.01; 0.01]	3.8%
Kim, 2011	201	2331	0.09	[0.08; 0.10]	3.7%
Razanajatovo, 2011	3	313	0.01	[0.00; 0.03]	3.7%
Balinandi, 2013	19	369	0.05	[0.03; 0.08]	3.3%
Kenmoe, 2016	14	347	0.04	[0.02; 0.07]	3.4%
Peterson, 2016	25	1505	0.02	[0.01; 0.02]	3.8%
Njouom, 2012	28	561	0.05	[0.03; 0.07]	3.5%
Dia, 2014 (1)	56	1427	0.04	[0.03; 0.05]	3.7%
Ahmed, 2012	359	6264	0.06	[0.05; 0.06]	3.8%
Pretorious, 2016	227	5743	0.04	[0.03; 0.04]	3.8%
Pretorious, 2012	303	8173	0.04	[0.03; 0.04]	3.8%
Thiam, 2015	95	2419	0.04	[0.03; 0.05]	3.7%
**Random effects model**	**2147**	**55232**	**0.05**	**[0.04; 0.06]**	**100.0%**

Heterogeneity: $I^2 = 97\%$, $\tau^2 = 0.0009$, $p < 0.01$

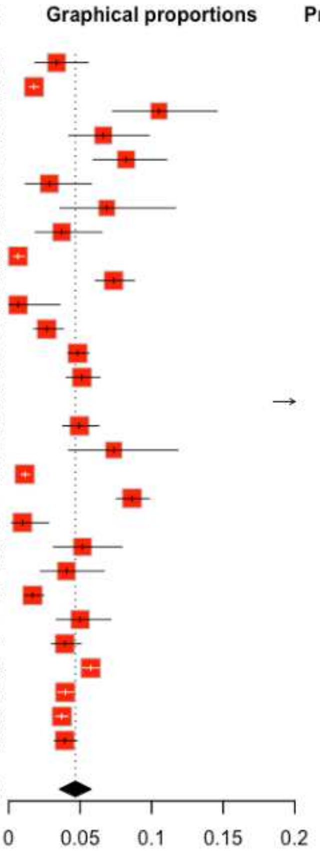

HPIV (36 studies)

Study	Events	Total	Proportion	95%-CI	Weight
Dia, 2014 (2)	13	232	0.06	[0.03; 0.09]	2.8%
Mohamed, 2015	24	419	0.06	[0.04; 0.08]	2.8%
Cohen, 2015 (2)	208	7052	0.03	[0.03; 0.03]	2.9%
Hoffman, 2012	13	295	0.04	[0.02; 0.07]	2.8%
O'Callaghan-Gordo, 2011 (2)	16	333	0.05	[0.03; 0.08]	2.8%
O'Callaghan-Gordo, 2011 (1)	31	475	0.07	[0.04; 0.09]	2.8%
Akinloye, 2011	77	246	0.31	[0.26; 0.37]	2.6%
Ghani, 2012	23	175	0.13	[0.09; 0.19]	2.6%
Simusika, 2015	13	297	0.04	[0.02; 0.07]	2.8%
Kabego, 2018	23	146	0.16	[0.10; 0.23]	2.6%
Adiku, 2015	42	108	0.39	[0.30; 0.49]	2.2%
Famoroti, 2018	124	2172	0.06	[0.05; 0.07]	2.9%
Johnson, 2008	36	419	0.09	[0.06; 0.12]	2.8%
Nunes, 2014	58	1460	0.04	[0.03; 0.05]	2.9%
Smuts, 2008	42	1055	0.04	[0.03; 0.05]	2.9%
Serengbe, 2015	34	361	0.09	[0.07; 0.13]	2.8%
Cohen, 2016	107	1410	0.08	[0.06; 0.09]	2.8%
Lagare, 2015	32	160	0.20	[0.14; 0.27]	2.5%
Kwofie, 2012	4	128	0.03	[0.01; 0.08]	2.8%
Feikin, 2012	122	1216	0.10	[0.08; 0.12]	2.8%
Feikin, 2013	8	204	0.04	[0.02; 0.08]	2.8%
Nakouné, 2013	11	329	0.03	[0.02; 0.06]	2.8%
Jallow, 2019	36	8209	0.00	[0.00; 0.01]	2.9%
Fall, 2016	98	5338	0.02	[0.01; 0.02]	2.9%
Lonngren, 2014	33	569	0.06	[0.04; 0.08]	2.8%
Razanajatovo, 2011	16	313	0.05	[0.03; 0.08]	2.8%
Balinandi, 2013	155	369	0.42	[0.37; 0.47]	2.6%
Kenmoe, 2016	23	347	0.07	[0.04; 0.10]	2.8%
Peterson, 2016	246	1505	0.16	[0.15; 0.18]	2.8%
Razanajatovo, 2018	31	876	0.04	[0.02; 0.05]	2.8%
Njouom, 2012	42	561	0.07	[0.05; 0.10]	2.8%
Dia, 2014 (1)	99	1427	0.07	[0.06; 0.08]	2.8%
Ahmed, 2012	591	6264	0.09	[0.09; 0.10]	2.9%
Pretorious, 2016	293	5743	0.05	[0.05; 0.06]	2.9%
Pretorious, 2012	528	8173	0.06	[0.06; 0.07]	2.9%
Thiam, 2015	126	2419	0.05	[0.04; 0.06]	2.9%
**Random effects model**	**3378**	**60805**	**0.09**	**[0.06; 0.12]**	**100.0%**

Heterogeneity: $I^2 = 99\%$, $\tau^2 = 0.0075$, $p = 0$

Graphical proportions

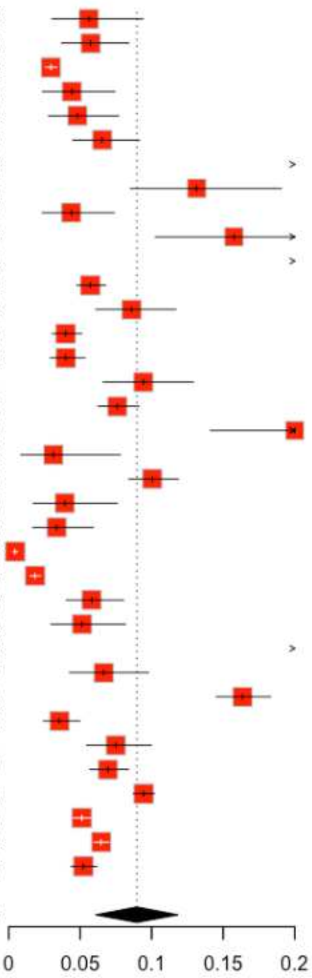

HRV (30 studies)

Study	Events	Total	Graphical proportions	Proportion	95%-CI	Weight
Dia, 2014 (2)	40	232		0.17	[0.13; 0.23]	3.3%
Cohen, 2015 (2)	1267	7052		0.18	[0.17; 0.19]	3.4%
Hoffman, 2012	38	295		0.13	[0.09; 0.17]	3.3%
O'Callaghan-Gordo, 2011 (2)	86	333		0.26	[0.21; 0.31]	3.3%
O'Callaghan-Gordo, 2011 (1)	196	475		0.41	[0.37; 0.46]	3.3%
Akinloye, 2011	7	246		0.03	[0.01; 0.06]	3.4%
Ghani, 2012	76	175		0.43	[0.36; 0.51]	3.1%
Simusika, 2015	34	297		0.11	[0.08; 0.16]	3.3%
Kabego, 2018	24	146		0.16	[0.11; 0.23]	3.2%
Famoroti, 2018	152	2172		0.07	[0.06; 0.08]	3.4%
Nunes, 2014	446	1460		0.31	[0.28; 0.33]	3.4%
Ouedraogo, 2014	88	153		0.58	[0.49; 0.65]	3.1%
Serengbe, 2015	16	361		0.04	[0.03; 0.07]	3.4%
Cohen, 2016	426	1410		0.30	[0.28; 0.33]	3.4%
Lagare, 2015	47	160		0.29	[0.22; 0.37]	3.1%
Cohen, 2015 (1)	3228	8723		0.37	[0.36; 0.38]	3.4%
Mazur, 2017	575	2322		0.25	[0.23; 0.27]	3.4%
Feikin, 2012	147	1216		0.12	[0.10; 0.14]	3.4%
Enan, 2013	14	334		0.04	[0.02; 0.07]	3.4%
Fall, 2016	152	5338		0.03	[0.02; 0.03]	3.4%
Razanajatovo, 2011	77	313		0.25	[0.20; 0.30]	3.3%
Balinandi, 2013	81	369		0.22	[0.18; 0.27]	3.3%
Kenmoe, 2016	40	347		0.12	[0.08; 0.15]	3.3%
Peterson, 2016	301	1505		0.20	[0.18; 0.22]	3.4%
Razanajatovo, 2018	125	876		0.14	[0.12; 0.17]	3.4%
Njouom, 2012	100	561		0.18	[0.15; 0.21]	3.4%
Dia, 2014 (1)	311	1427		0.22	[0.20; 0.24]	3.4%
Pretorious, 2016	1731	5743		0.30	[0.29; 0.31]	3.4%
Pretorious, 2012	2034	8173		0.25	[0.24; 0.26]	3.4%
Thiam, 2015	649	2419		0.27	[0.25; 0.29]	3.4%
**Random effects model**	**12508**	**54633**		**0.21**	**[0.17; 0.26]**	**100.0%**

Heterogeneity: $I^2 = 100\%$, $\tau^2 = 0.0154$, $p = 0$

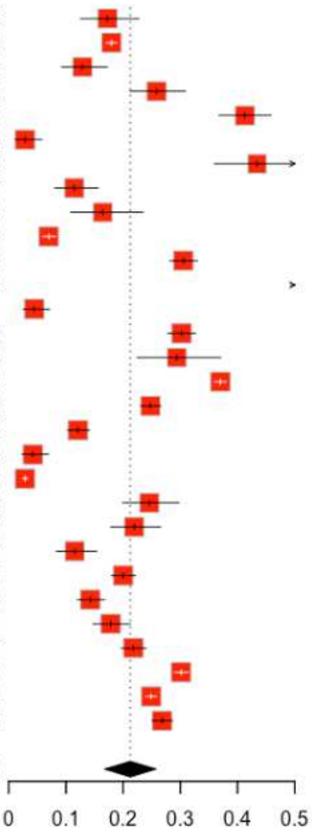

Enterovirus (22 studies)

Study	Events	Total	Graphical proportions	Proportion	95%-CI	Weight
Cohen, 2015 (2)	119	7052		0.02	[0.01; 0.02]	4.7%
Hoffman, 2012	1	295		0.00	[0.00; 0.02]	4.7%
O'Callaghan-Gordo, 2011 (2)	9	333		0.03	[0.01; 0.05]	4.6%
O'Callaghan-Gordo, 2011 (1)	18	475		0.04	[0.02; 0.06]	4.6%
Akinloye, 2011	7	246		0.03	[0.01; 0.06]	4.6%
Kabego, 2018	2	146		0.01	[0.00; 0.05]	4.6%
Famoroti, 2018	38	2172		0.02	[0.01; 0.02]	4.7%
Ouedraogo, 2014	38	153		0.25	[0.18; 0.32]	3.7%
Serengbe, 2015	16	361		0.04	[0.03; 0.07]	4.6%
Cohen, 2016	102	1410		0.07	[0.06; 0.09]	4.6%
Lagare, 2015	18	160		0.11	[0.07; 0.17]	4.1%
Cohen, 2015 (1)	872	8723		0.10	[0.09; 0.11]	4.7%
Howie, 2014	1	55		0.02	[0.00; 0.10]	4.4%
Feikin, 2012	109	1216		0.09	[0.07; 0.11]	4.6%
Fall, 2016	147	5338		0.03	[0.02; 0.03]	4.7%
Kenmoe, 2016	40	347		0.12	[0.08; 0.15]	4.4%
Peterson, 2016	50	1505		0.03	[0.02; 0.04]	4.7%
Njouom, 2012	33	561		0.06	[0.04; 0.08]	4.6%
Dia, 2014 (1)	257	1427		0.18	[0.16; 0.20]	4.6%
Pretorious, 2016	208	5743		0.04	[0.03; 0.04]	4.7%
Pretorious, 2012	515	8173		0.06	[0.06; 0.07]	4.7%
Thiam, 2015	584	2419		0.24	[0.22; 0.26]	4.6%
**Random effects model**	**3184**	**48310**		**0.07**	**[0.04; 0.10]**	**100.0%**

Heterogeneity: $I^2 = 99\%$, $\tau^2 = 0.0044$, $p = 0$

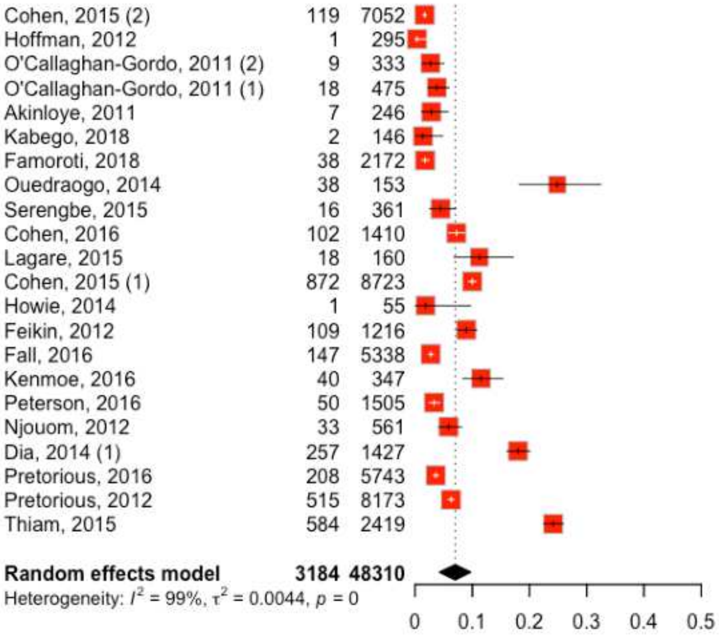

AdV (37 studies)

Study	Events	Total	Proportion	95%-CI	Weight
Cohen, 2015 (2)	613	7052	0.09	[0.08; 0.09]	2.7%
Hoffman, 2012	9	295	0.03	[0.01; 0.06]	2.7%
O'Callaghan-Gordo, 2011 (2)	47	333	0.14	[0.11; 0.18]	2.7%
O'Callaghan-Gordo, 2011 (1)	102	475	0.21	[0.18; 0.25]	2.7%
Akinloye, 2011	12	246	0.05	[0.03; 0.08]	2.7%
Ghani, 2012	30	175	0.17	[0.12; 0.24]	2.6%
Simusika, 2015	27	297	0.09	[0.06; 0.13]	2.7%
Kabego, 2018	7	146	0.05	[0.02; 0.10]	2.7%
Adiku, 2015	10	108	0.09	[0.05; 0.16]	2.6%
Famoroti, 2018	215	2172	0.10	[0.09; 0.11]	2.7%
Johnson, 2008	9	419	0.02	[0.01; 0.04]	2.7%
Nunes, 2014	29	1460	0.02	[0.01; 0.03]	2.7%
Smuts, 2008	65	1055	0.06	[0.05; 0.08]	2.7%
Serengbe, 2015	10	361	0.03	[0.01; 0.05]	2.7%
Cohen, 2016	165	1410	0.12	[0.10; 0.13]	2.7%
Lagare, 2015	105	160	0.66	[0.58; 0.73]	2.5%
Kwofie, 2012	13	128	0.10	[0.06; 0.17]	2.6%
Cohen, 2015 (1)	2268	8723	0.26	[0.25; 0.27]	2.7%
Howie, 2014	2	55	0.04	[0.00; 0.13]	2.6%
Mazur, 2017	347	2322	0.15	[0.14; 0.16]	2.7%
Feikin, 2012	113	1216	0.09	[0.08; 0.11]	2.7%
Feikin, 2013	33	204	0.16	[0.11; 0.22]	2.6%
Enan, 2013	2	334	0.01	[0.00; 0.02]	2.7%
Jallow, 2019	9	8209	0.00	[0.00; 0.00]	2.7%
Fall, 2016	215	5338	0.04	[0.04; 0.05]	2.7%
Kim, 2011	679	2331	0.29	[0.27; 0.31]	2.7%
Razanajatovo, 2011	19	313	0.06	[0.04; 0.09]	2.7%
Balinandi, 2013	170	369	0.46	[0.41; 0.51]	2.6%
Kenmoe, 2016	95	347	0.27	[0.23; 0.32]	2.7%
Peterson, 2016	136	1505	0.09	[0.08; 0.11]	2.7%
Razanajatovo, 2018	77	876	0.09	[0.07; 0.11]	2.7%
Niang, 2017	1967	6381	0.31	[0.30; 0.32]	2.7%
Dia, 2014 (1)	363	1427	0.25	[0.23; 0.28]	2.7%
Ahmed, 2012	1361	6264	0.22	[0.21; 0.23]	2.7%
Pretorious, 2016	813	5743	0.14	[0.13; 0.15]	2.7%
Pretorious, 2012	1063	8173	0.13	[0.12; 0.14]	2.7%
Thiam, 2015	633	2419	0.26	[0.24; 0.28]	2.7%
**Random effects model**	**11833**	**78841**	**0.14**	**[0.10; 0.19]**	**100.0%**

Heterogeneity: $I^2 = 100\%$, $\tau^2 = 0.0169$, $p = 0$

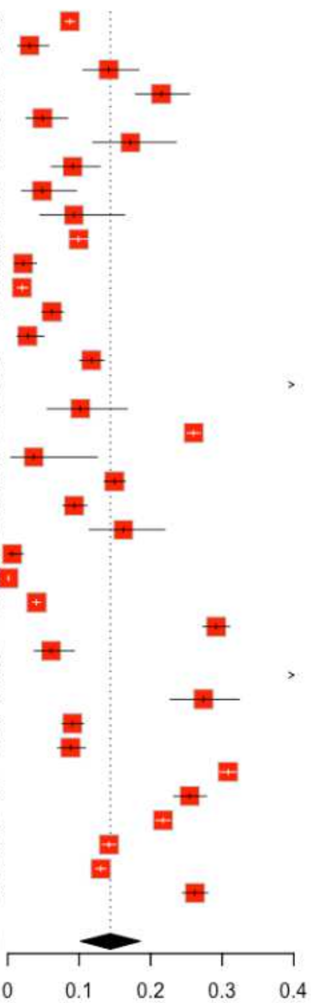

HBoV (12 studies)

Study	Events	Total	Proportion	95%-CI	Weight
Hoffman, 2012	1	295	0.00	[0.00; 0.02]	8.9%
Akinloye, 2011	6	246	0.02	[0.01; 0.05]	8.3%
Kabego, 2018	2	146	0.01	[0.00; 0.05]	8.4%
Famoroti, 2018	37	2172	0.02	[0.01; 0.02]	8.9%
Nunes, 2014	174	1460	0.12	[0.10; 0.14]	8.5%
Serengbe, 2015	5	361	0.01	[0.00; 0.03]	8.7%
Howie, 2014	2	55	0.04	[0.00; 0.13]	6.1%
Razanajatovo, 2011	2	313	0.01	[0.00; 0.02]	8.8%
Kenmoe, 2016	37	347	0.11	[0.08; 0.14]	7.4%
Peterson, 2016	102	1505	0.07	[0.06; 0.08]	8.7%
Razanajatovo, 2018	40	876	0.05	[0.03; 0.06]	8.6%
Dia, 2014 (1)	79	1427	0.06	[0.04; 0.07]	8.7%
**Random effects model**	**487**	**9203**	**0.04**	**[0.02; 0.06]**	**100.0%**

Heterogeneity: $I^2 = 96\%$, $\tau^2 = 0.0014$, $p < 0.01$

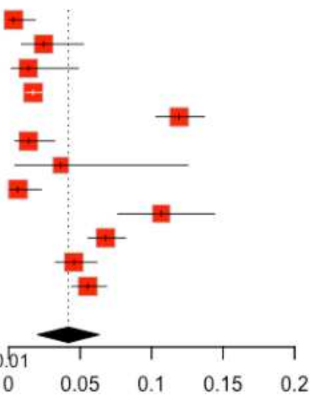

HCoV (14 studies)

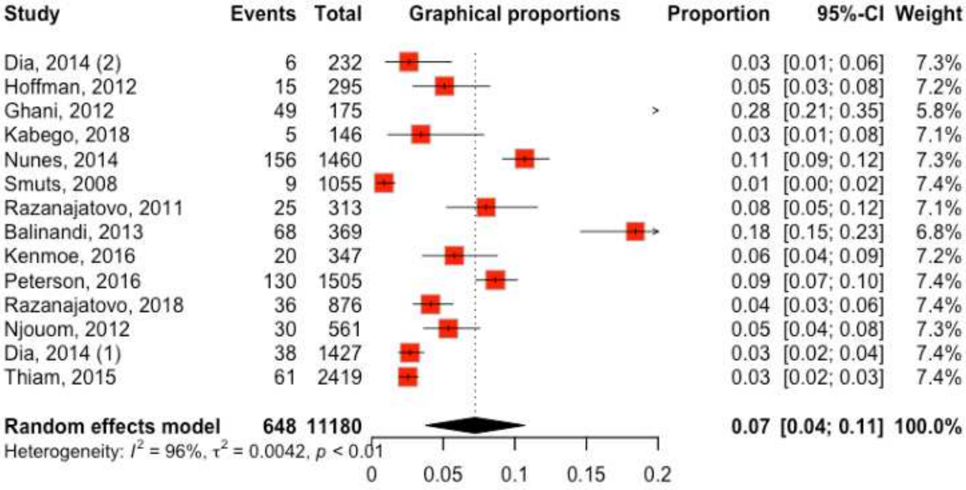

Supplement: Supplementary file 1 — Supplementary Material 1 [file 12879_2025_12122_MOESM1_ESM.zip › Fig. S1.pdf]
