# Supplementary material for: Viral etiology of acute respiratory infections in Sub-Saharan Africa during the pre-COVID-19 period (2006–2019): a systematic review and meta-analysis
Source: BMC Infect Dis. 2025 Nov 23;25:1799. doi: 10.1186/s12879-025-12122-8 (PMC12750592; doi:10.1186/s12879-025-12122-8)
Supplement: Supplementary file 1 — Supplementary Material 1 [file 12879_2025_12122_MOESM1_ESM.zip › Table S2.pdf]

Table S2: Search Strategy

Pubmed

Search	Search terms
#1	epidemiology/
#2	epidemiology*
#3	etiology
#4	causality/
#5	caus*
#6	((enabling or predisposing or reinforcing) adj2 factor*)
#7	Prevalence
#8	prevalence*
#9	Pathogenesis
#10	influenza, Human
#11	Influenza
#12	Grippes
#13	human adj2 flu
#14	respiratory tract infections
#15	(respiratory adj2 (disease or infection))
#16	Coinfection
#17	(co infection or co-infection or coinfection)
#18	((mixed or secondary or polymicrobial) adj2 infection)
#19	concomittant infection
#20	concurrent infection
#21	"Africa South of the Sahara"
#22	((sub-saharan or subsaharan) adj1 africa)
#23	"africa south of the sahara"
#24	#1 OR #2 OR #3 OR #4 OR #5 OR #6 OR #7 OR #8 OR #9

#25	#10 OR #11 OR #12 OR #13 OR #14 OR #15 OR #16 OR #17 OR #18
#26	#24 AND #25
#27	(#26 AND (#21 OR #22 OR #23))
#28	Filters: from 2006-2019

EMBASE

Search	Search terms
#1	'africa south of the sahara'/exp OR 'africa south of the sahara'
#2	'epidemiology'/exp
#3	'etiology'/exp
#4	'causality'/exp
#5	'enabling factor'
#6	'reinforcing factor'
#7	'predisposing factor'/exp
#8	'prevalence'/exp
#9	'pathogenesis'/exp
#10	'influenza'/ exp'epidemiology'/exp OR 'etiology'/exp OR 'causality'/exp OR 'enabling factor' OR 'reinforcing factor' OR 'predisposing factor'/exp OR 'prevalence'/exp OR 'pathogenesis'/exp
#11	'influenza'/exp
#12	'respiratory tract disease'/exp
#13	'respiratory tract infection'/exp
#14	'coinfection'/exp
#15	'secondary infection'/exp
#16	'concomittant infection'
#17	'influenza'/exp OR 'respiratory tract disease'/exp OR 'respiratory tract infection'/exp OR 'coinfection'/exp OR 'co infection'/exp OR 'secondary infection'/exp OR 'concomittant infection'

#18	(#1 AND #2 AND #3 AND #4 AND #5 AND #6 AND #7 AND #8 AND #9 AND #10 AND #17) AND ('influenza'/exp OR 'respiratory tract disease'/exp OR 'respiratory tract infection'/exp OR 'coinfection'/exp OR 'co infection'/exp OR 'secondary infection'/exp OR 'concomittant infection')
#19	(#1 AND #2 AND #3 AND #4 AND #5 AND #6 AND #7 AND #8 AND #9 AND #10 AND #17 AND ([english]/lim OR [french]/lim))
#20	(#1 AND #2 AND #3 AND #4 AND #5 AND #6 AND #7 AND #8 AND #9 AND #10 AND #17 AND ([english]/lim OR [french]/lim) AND (2006 OR 2007 OR 2008 OR 2009 OR 2010 OR 2011 OR 2012 OR 2013 OR 2014 OR 2015 OR 2016 OR 2017 OR 2018 OR 2019)) AND ('coinfection'/dm OR 'epidemic'/dm OR 'infection'/dm OR 'influenza'/dm OR 'lower respiratory tract infection'/dm OR 'respiratory distress'/dm OR 'respiratory tract disease'/dm OR 'respiratory tract infection'/dm) AND 'article'/it AND 'epidemiology'/lnk

OVID MEDLINE

Search	Search terms
#1	epidemiology/
#2	epidemiolog*.ti,ab,kf.
#3	etiology.ti,ab,kf.
#4	Causality/
#5	caus*.ti,ab,kf.
#6	((enabling or predisposing or reinforcing) adj2 factor*).ti,ab,kf.
#7	Prevalence/
#8	prevalence*.ti,ab,kf.
#9	pathogenesis.ti,ab,kf.
#10	Influenza, Human/
#11	influenza*.ti,ab,kf.

#12	grippe.ti,ab,kf.
#13	(human adj2 flu).ti,ab,kf.
#14	Respiratory Tract Infections/
#15	(respiratory adj2 (disease* or infection*)).ti,ab,kf.
#16	Coinfection/
#17	(co infection* or co-infection* or coinfection*).ti,ab,kf.
#18	((mixed or secondary or polymicrobial) adj2 infection*).ti,ab,kf.
#19	concomittant infection*.ti,ab,kf.
#20	concurrent infection*.ti,ab,kf.
#21	"Africa South of the Sahara"/
#22	((sub-saharan or subsaharan) adj1 africa).ti,ab,kf.
#23	"africa south of the sahara".ti,ab,kf.
#24	(epidemiolog*.ti,ab,kf OR etiology.ti,ab,kf OR caus*.ti,ab,kf OR (enabling OR predisposing OR reinforcing) adj2 factor*.ti,ab,kf OR prevalence*.ti,ab,kf OR pathogenesis.ti,ab,kf)
#25	(influenza*.ti,ab,kf OR grippe.ti,ab,kf OR (human adj2 flu).ti,ab,kf)
#26	(co infection* OR co-infection* OR coinfection* OR (mixed OR secondary OR polymicrobial) adj2 infection* OR concomittant infection* OR concurrent infection*).ti,ab,kf
#27	((sub-saharan OR subsaharan) adj1 africa OR "africa south of the sahara").ti,ab,kf
#28	(epidemiolog*.ti,ab,kf OR etiology.ti,ab,kf OR caus*.ti,ab,kf OR (enabling OR predisposing OR reinforcing) adj2 factor*.ti,ab,kf OR prevalence*.ti,ab,kf OR pathogenesis.ti,ab,kf) AND (influenza*.ti,ab,kf OR grippe.ti,ab,kf OR (human adj2 flu).ti,ab,kf) AND (respiratory adj2 (disease* OR infection*)).ti,ab,kf AND (co infection* OR co-infection* OR coinfection* OR (mixed OR secondary OR polymicrobial) adj2 infection* OR concomittant infection* OR concurrent infection*).ti,ab,kf, AND ((sub-saharan OR subsaharan) adj1 africa OR "africa south of the sahara").ti,ab,kf
#29	Filtre to 2006-2019

SCOPUS

Search	Search terms
#1	TITLE-ABS-KEY (prevalence*)

#2	TITLE-ABS-KEY (pathogenesis)
#3	TITLE-ABS-KEY (enabling OR predisposing OR reinforcing) W/2 factor*)
#4	TITLE-ABS-KEY (caus*)
#5	TITLE-ABS-KEY (etiology)
#6	TITLE-ABS-KEY (epidemiolog*)
#7	TITLE-ABS-KEY (influenza*)
#8	TITLE-ABS-KEY (human W/2 flu)) TITLE-ABS-KEY (grippe)
#9	TITLE-ABS-KEY (respiratory W/2 infection*)) OR (respiratory tract disease*)
#10	TITLE-ABS-KEY (coinfection* OR co-infection* OR "co infection*")
#11	TITLE-ABS-KEY ((mixed OR polymicrobial OR secondary) W/2 infection*)
#12	TITLE-ABS-KEY ((concomitant OR concurrent) W/1 infection*)
#13	TITLE-ABS-KEY ("africa south of the sahara")
#14	TITLE-ABS-KEY ((subsaharan OR sub-saharan) W/1 africa)
#15	TITLE-ABS-KEY (prevalence* OR pathogenesis OR (enabling OR predisposing OR reinforcing) W/2 factor* OR caus* OR etiology OR epidemiolog*)
#16	TITLE-ABS-KEY (influenza* OR human W/2 flu OR grippe OR respiratory W/2 infection* OR respiratory AND disease* OR (coinfection* OR co-infection* OR "co infection*") OR (mixed OR polymicrobial OR secondary) W/2 infection* OR (concomitant OR concurrent) W/1 infection*)
#17	TITLE-ABS-KEY ("africa south of the sahara" OR subsaharan OR sub-saharan W/1 africa)
#18	TITLE-ABS-KEY ((((prevalence*) OR (pathogenesis)) OR ((enabling OR predisposing OR reinforcing) W/2 factor*)) OR ((caus*) OR (etiology) OR (epidemiolog*)) AND ((influenza*) OR (human W/2 flu) OR (grippe) OR (respiratory W/2 infection*) OR (respiratory AND desease*) OR ((coinfection* OR co-infection* OR "co infection*")) OR ((mixed OR polymicrobial OR secondary) W/2 infection*) OR ((concomitant OR concurrent) W/1 infection*) AND (("africa south of the sahara") OR (subsaharan OR sub-saharan) W/1 africa))) AND AND (LIMIT-TO (PUBYEAR , 2019) OR LIMIT-TO (PUBYEAR , 2018) OR LIMIT-TO (PUBYEAR , 2017) OR LIMIT-TO (PUBYEAR , 2016) OR LIMIT-TO (PUBYEAR , 2015) OR LIMIT-TO (PUBYEAR , 2014) OR LIMIT-TO (PUBYEAR , 2013) OR LIMIT-TO (PUBYEAR , 2012) OR LIMIT-TO (PUBYEAR , 2011) OR LIMIT-TO (PUBYEAR , 2010) OR LIMIT-TO (PUBYEAR , 2009) OR LIMIT-TO (PUBYEAR , 2008) OR LIMIT-TO (PUBYEAR , 2007) OR LIMIT-TO (PUBYEAR , 2006)) AND (LIMIT-TO (DOCTYPE , "ar"))

Search sources	Execution date	Number of results	Summary
PubMed		829	Total searched = 3098

<table>
<tr><td>Ovid/Medline</td><td rowspan="3">August 10, 2019</td><td>594</td><td rowspan="5">Duplicate removed =1228
Total screened removal of duplicates=1870
Total Full Text=137
Total extracted= 73</td></tr>
<tr><td>Embase</td><td>970</td></tr>
<tr><td>Scopus</td><td>661</td></tr>
<tr><td>Manually seach</td><td>August 12, 2019</td><td>44</td></tr>
<tr><td></td><td></td><td>Total searched =
829+594+970+661+44= 3098</td></tr>
</table>

Total searched =3098

Total screened =1870

Total Full Text =137

Total extracted =73
