# Supplementary material for: Viral etiology of acute respiratory infections in Sub-Saharan Africa during the pre-COVID-19 period (2006–2019): a systematic review and meta-analysis
Source: BMC Infect Dis. 2025 Nov 23;25:1799. doi: 10.1186/s12879-025-12122-8 (PMC12750592; doi:10.1186/s12879-025-12122-8)
Supplement: Supplementary file 1 — Supplementary Material 1 [file 12879_2025_12122_MOESM1_ESM.zip › Table S3.pdf]

Table S3: Items for risk of bias

**Items rating scale for studies**	**Yes (1)/No (0)**
**External validity**	
(a) Was the participation response rate more than 75%, or was there an analysis to show whether respondents and non-respondents were similar in sociodemographic characteristics?	**1**
(b) Was the clinical presentation clearly defined?	**1**
(c) Was the method of inclusion identical for all subjects?	**1**
(d) Was the diagnostic technique described?	**1**
(e) Was the same type of sample collected from all patients (e.g., nasopharyngeal aspirate, nasal swab, or throat swab)?	**1**
(f) Was there a standardized method for sample collection (e.g., quantity of aspirate or liquid used for the nasal wash, or any virological medium used for swabs)?	**1**
(g) Was analysis performed according to relevant subgroups (e.g., by age classes, by center, or by symptomatology)?	**1**
(h) Was the presentation of data sources clear (e.g., were counts presented, not just percentages)?	**1**
(a) Was the participation response rate more than 75%, or was there an analysis to show whether respondents and non-respondents were similar in sociodemographic characteristics?	**1**
Total score	**8**
**Risk of Bias Assessment**<br>Total Score 6-8: Low risk of bias<br>Total Score 3-5: Moderate risk of bias<br>Total Score 0-2: High risk of bias	
