# Supplementary material for: Viral etiology of acute respiratory infections in Sub-Saharan Africa during the pre-COVID-19 period (2006–2019): a systematic review and meta-analysis
Source: BMC Infect Dis. 2025 Nov 23;25:1799. doi: 10.1186/s12879-025-12122-8 (PMC12750592; doi:10.1186/s12879-025-12122-8)
Supplement: Supplementary file 1 — Supplementary Material 1 [file 12879_2025_12122_MOESM1_ESM.zip › Table S4.pdf]

Table S4-1: Characteristics of included studies

Study	Design	Setting	Period	Age group	Age group	Children %	Clinical presentation	WHO Definition Criteriaa	Country	Africa Region	Samples	Diagnostic
Kabego, 2018	Cross-sectional Prospective	Urban	Feb-Dec/2001	Children	0-5y	100	SRTI	Yes	Ghana	Western	Nasopharyngeal	rRT-PCR
Kenmoe, 2016	Cross-sectional Prospective	Urban and Rural	Sep-2007-Aug-2010	Children and Adults	na	45.4	BRTI and SRTI	Yes	Kenya	Eastern	Nasopharyngeal and Oropharyngeal	rRT-PCR
Lekana-Douki, 2014	Cross sectional Prospective	Urban and Rural	Jan/2012-Dec/2012	Children	0-5y	100	SRTI	No	Kenya	Eastern	Nasopharyngeal	rRT-PCR
Nakouné, 2013	Cross-sectional Prospective	Unclear/ Not described	Feb/2009-May/2009	Children	0-5y	100	BRTI and SRTI	No	Nigeria	Western	Throat and Nasal	rRT-PCR
Njouom, 2010	Cross sectional Prospective	Urban	Aug/2008-Dec/2008	Children and Adults	na	35	BRTI	Yes	Uganda	Eastern	Nasopharyngeal	rRT-PCR
Njouom, 2012	Cross sectional Prospective	Urban and Rural	Feb/2009-Dec/2012	Children	0-5y	100	SRTI	Yes	South Africa	Southern	Nasopharyngeal	rRT-PCR
Serengbe, 2015	Cross sectional Prospective	Urban and Rural	Feb/2009-Dec/2012	Children and Adults	>5y	8	SRTI	Yes	South Africa	Southern	Nasopharyngeal	rRT-PCR
Tamfum, 2012	Cross-sectional Prospective	Urban and Rural	Jan/2010-Dec/2013	Children	0-5y	100	SRTI	Yes	South Africa	Southern	Nasopharyngeal	rRT-PCR
Ahmed, 2012	Cross-sectional Prospective	Urban and Rural	Mar/2012-Jul/2013	Children and Adults	na	70	BRTI	Yes	Senegal	Western	Nasopharyngeal and Oropharyngeal	rRT-PCR
Agoti, 2014	Cross-sectional Prospective	Unclear/ Not described	Jan/2009-Dec/2011	Adults	>5y	0	BRTI	Yes	Sénégal	Western	Nasopharyngeal and Oropharyngeal	rRT-PCR
Balinandi, 2013	Cross sectional Prospective	Rural	Aug/2009–Jul/2012	Children and Adults	na	76	BRTI and SRTI	Yes	Kenya	Eastern	Nasopharyngeal and Oropharyngeal	rRT-PCR
Emukule, 2014	Cross-sectional Prospective	Unclear/ Not described	Jan/2010-Mar/2010	Children and Adults	na	92	BRTI	Yes	Sudan	Eastern	Throat	rRT-PCR
Enan, 2013	Cross-sectional Prospective	Unclear/ Not described	Jan/2012-Oct/2015	Children and Adults	na	52.5	BRTI	Yes	Senegal	Western	Nasopharyngeal and Oropharyngeal	rRT-PCR

Feikin, 2012	Cross-sectional Retrospective	Urban	Jan/2011-Jul/2015	Children	0-5y	100	SRTI	Yes	South Africa	Southern	Nasopharyngeal	rRT-PCR
Feikin, 2013	Cohort Prospective	Rural	Mar/2007-Feb/2010	Children and Adults	na	100	BRTI and SRTI	Yes	Kenya	Eastern	Nasopharyngeal	rRT-PCR
Ho, 2018	Randomized Controlled Trial Prospective	Rural	Jan/2009-Feb/2010	Children and Adults	na	na	BRTI and SRTI	Yes	Kenya	Eastern	Nasopharyngeal and Oropharyngeal	rRT-PCR
Hoffman, 2012	Cross-sectional Retrospective	Unclear/ Not described	Apr/2009-Dec/2009	Children	0-5y	100	SRTI	Yes	South Africa	Southern	Nasopharyngeal/Tracheal/ Broncho-alveolar lavage	rRT-PCR
Katz, 2014	Cross-sectional Prospective	Urban	Jan/2011-Dec/2013	Adults	>5y	0	SRTI	Yes	Malawi	Eastern	Nasopharyngeal	rRT-PCR
Kim, 2011	Cross-sectional Prospective	Rural	Feb/2010-Feb/2011	Children	0-5y	100	BRTI and SRTI	Yes	Madagascar	Eastern	Nasopharyngeal	rRT-PCR
Mmbaga,2012	Cross sectional Prospective	Urban and Rural	2007-2009	Children	0-5y	100	SRTI	Yes	Gambia	Western	Nasopharyngeal	rRT-PCR
Mohamed, 2015	Cross-sectional Retrospective	Unclear/ Not described	Jan/2012-Dec/2016	Children and Adults	na	49.7	BRTI	Yes	Senegal	Western	Nasopharyngeal and Oropharyngeal	rRT-PCR
Nyatanyi, 2012	Cross-sectional Prospective	Urban	Not described	Children	0-5y	100	SRTI	No	Nigeria	Western	Nasopharyngeal	rRT-PCR
Nyawanda, 2016	Cross-sectional Prospective	Urban	Aug-Dec/2016	Children	0-5y	100	BRTI and SRTI	Yes	Democratic Republic of Congo	Central	Nasopharyngeal	rRT-PCR
Onyango, 2012	Cross sectional Prospective	Urban and Rural	Jan/2003-Dec/2010	Children and Adults	na	52	BRTI	Yes	Côte d'Ivoire	Western	Nasopharyngeal	rRT-PCR
Owor, 2016	Cross-sectional Prospective	Urban and Rural	Jul 2007-Jun/2013	Children and Adults	na	88.5	BRTI and SRTI	Yes	Kenya	Eastern	Nasopharyngeal and Oropharyngeal	rRT-PCR
Peterson, 2016	Cross-sectional Prospective	Urban	Sep/2011-Sep/2013	Children	na	100	SRTI	No	Cameroon	Central	Nasopharyngeal	rRT-PCR
Razanajatovo, 2011	Cross-sectional Prospective	Unclear/ Not described	Jun/2009-Aug/2010	Children and Adults	na	81.6	BRTI and SRTI	Yes	Kenya	Eastern	Nasopharyngeal and Oropharyngeal	rRT-PCR
Razanajatovo, 2018	Cross sectional Prospective	Urban and Rural	Jan/2008-Dec/2008	Children	0-5y	100	SRTI	Yes	Ghana	Western	Nasopharyngeal	rRT-PCR

Wabwire-Mangen, 2016	Cross-sectional Retrospective	Urban and Rural	Jan/2010-Dec/2012	Children	0-5y	100	SRTI	Yes	Niger	Western	Nasopharyngeal	rRT-PCR
Woyessa, 2018	Cross-sectional Prospective	Urban and Rural	Mar/2010-Jun/2011	Children and Adults	na	75.5	BRTI	Yes	Gabon	Central	Nasal	rRT-PCR
Cohen, 2015 (1)	Cross-sectional Prospective	Unclear/ Not described	Jul/2009-Jul/2011	Children	na	100	BRTI and SRTI	No	South Africa	Southern	Nasopharyngeal and Broncho-alveolar lavage	rRT-PCR
Cohen, 2015 (2)	Cohort Prospective	Rural	Feb-Dec/2015	Children	0-5y	100	SRTI	Yes	Gambia	Western	Nasopharyngeal and Oropharyngeal	rRT-PCR
Cohen, 2016	Cross-sectional Prospective	Urban	Apr/2009-2013	Children and Adults	na	36.2	BRTI and SRTI	Yes	Niger	Western	Nasopharyngeal	rRT-PCR
Famoroti, 2018	Cross-sectional Prospective	Urban	Apr./2010-Dec/2012	Children and Adults	na	66	BRTI	Yes	Togo	Western	Nasopharyngeal	rRT-PCR
Ghani, 2012	Cross-sectional Prospective	Urban and Rural	Feb/2009-Dec/2013	Children	0-5y	100	SRTI	No	South Africa	Southern	Nasopharyngeal	rRT-PCR
Lonngren, 2014	Cross-sectional Prospective	Urban and Rural	May/2008-Nov/2010	Children and Adults	na	38.6	BRTI and SRTI	Yes	Tanzania	Eastern	Nasopharyngeal and Oropharyngeal	rRT-PCR
Mazur, 2017	Cross-sectional Prospective	Unclear/ Not described	Jan/2010-Dec/2012	Children and Adults	>5y	0	BRTI and SRTI	Yes	Kenya	Eastern	Nasopharyngeal and Oropharyngeal	rRT-PCR
Moyes, 2013	Cross sectional Prospective	Urban and Rural	Jan/2010-Dec/2011	Children	0-5y	100	SRTI	No	South Africa	Southern	Nasopharyngeal	rRT-PCR
Moyes, 2017	Cross sectional Prospective	Urban and Rural	Feb/2009-Dec/2013	Adults	>5y	0	SRTI	Yes	South Africa	Southern	Nasopharyngeal and Oropharyngeal	rRT-PCR
Nunes, 2014	Cross-sectional Prospective	Rural	Jan/2010-Dec/2010	Children	na	100	BRTI and SRTI	Yes	Central African Republic	Central	Nasopharyngeal	rRT-PCR
O'Callaghan-Gordo, 2011 (1)	Cross-sectional Prospective	Urban	Jan/1996-Dec/2009	Children and Adults	na	64	BRTI	Yes	Senegal	Western	Nasal	Conventional RT-PCR
O'Callaghan-Gordo, 2011 (2)	Cross-sectional Prospective	Urban and Rural	Jan/2010-Dec/2015	Children and Adults	na	51.7	BRTI	Yes	Sénégal	Western	Nasopharyngeal and Oropharyngeal	rRT-PCR
Pale, 2017	Cross-sectional Prospective	Urban	Nov/2007-Oct/2008	Children and Adults	na	34	BRTI	Yes	Cameroon	Central	Nasopharyngeal	rRT-PCR

Pretorious, 2012	Cross-sectional Prospective	Urban and Rural	Jan/2009-Dec/2009	Children and Adults	na	74.2	BRTI	Yes	Cameroon	Central	Throat and Nasopharyngeal	rRT-PCR
Pretorious, 2013	Randomized controlled trial Retrospective	Urban	Mar/1998-Oct/2000	Children	0-5y	100	SRTI	Yes	South Africa	Southern	Nasopharyngeal	rRT-PCR
Pretorious, 2016	Cross-sectional Prospective	Urban and Rural	Jul/2008-May/2009	Children and Adults	na	14.2	BRTI and SRTI	Yes	Rwanda	Eastern	Nasopharyngeal and Oropharyngeal	rRT-PCR
Simusika, 2015	Cross-sectional Prospective	Rural	Sep/2009-Aug/2013	Children	0-5y	100	BRTI and SRTI	Yes	Kenya	Eastern	Nasopharyngeal and Oropharyngeal	rRT-PCR
Smuts, 2008	Cross-sectional Prospective	Urban	Feb/2006-Nov/2006	Children	0-5y	100	SRTI	No	Ghana	Western	Nasopharyngeal	Conventional RT-PCR
Theo, 2012	Cross-sectional Prospective	Urban	2006 and 2013-2014	Children	0-5y	100	SRTI	Yes	Ghana	Western	Nasopharyngeal	rRT-PCR
Adiku, 2015	Cross sectional Prospective	Rural	Feb/1999-May-2000	Children	0-5y	100	SRTI	Yes	Mozambique	Southern	Nasopharyngeal	rRT-PCR
Akinloye, 2011	Cross-sectional Prospective	Rural	Sep/2006-Sep/2007	Children	0-5y	100	SRTI	Yes	Mozambique	Southern	Nasopharyngeal	Conventional RT-PCR
Dia, 2014 (1)	Cross-sectional Prospective	Urban	Not described	Children	0-5y	100	SRTI	Yes	Nigeria	Western	Nasal	rRT-PCR
Dia, 2014 (2)	Cross-sectional Prospective	Rural	Jan/2007-Dec/2010	Children	na	100	SRTI	No	Kenya	Eastern	Nasopharyngeal	rRT-PCR
Fall, 2016	Cross-sectional Prospective	Urban	Jul/2010-Jun/2011	Children	0-5y	100	SRTI	Yes	Burkina Faso	Western	Nasopharyngeal	rRT-PCR
Howie, 2014	Cross-sectional Prospective	Urban	Jul/2010-Jun/2011	Children	0-5y	100	SRTI	Yes	Burkina Faso	Western	Nasopharyngeal	rRT-PCR
Jallow, 2019	Cross-sectional Prospective	Urban	Jan/2007-Dec/2011	Children	0-5y	100	SRTI	Yes	Kenya	Eastern	Nasopharyngeal	rRT-PCR
Johnson, 2008	Cross-sectional Prospective	Urban	Jan/15-Jan 16	Children	0-5y	100	SRTI	Yes	Mozambique	Southern	Nasopharyngeal	rRT-PCR
Kadjo, 2012	Cross-sectional Prospective	Urban	Jan/2011-Dec/2014	Children	na	100	SRTI	Yes	Malawi	Eastern	Nasopharyngeal	rRT-PCR
Kwofie, 2012	Cross-sectional Prospective	Urban and Rural	Feb/2009-Mar/2012	Children and Adults	na	na	SRTI	Yes	South Africa	Southern	Nasopharyngeal and Oropharyngeal	rRT-PCR

Lagare, 2015	Cross-sectional Retrospective	Urban and Rural	Feb/2009-May/2012	Children and Adults	na	27	SRTI	Yes	South Africa	Southern	Nasopharyngeal and Oropharyngeal	rRT-PCR
Mackenzie, 2019	Cross-sectional Prospective	Urban and Rural	Jan/2011-Dec/2014	Children	na	100	SRTI	Yes	South Africa	Southern	Nasopharyngeal and Oropharyngeal	rRT-PCR
Maïnassara, 2015	Cross sectional Retrospective	Urban	Aug/2009-Feb/2010	Children and Adults	na	49.2	BRTI	Yes	Madagascar	Eastern	Nasopharyngeal and Oropharyngeal	rRT-PCR
Maman, 2014	Cross-sectional Prospective	Urban	Nov/2010-Jul/2013	Children and Adults	na	81.1	SRTI	Yes	Madagascar	Eastern	Nasopharyngeal	rRT-PCR
Niang, 2012	Cross-sectional Prospective	Urban	Jan/2014-Dec/2015	Children	0-5y	100	BRTI and SRTI	Yes	Burkina Faso	Western	Nasopharyngeal	rRT-PCR
Niang, 2017	Cross-sectional Prospective	Urban	Sep2012-Aug/2013	Children	0-5y	100	BRTI and SRTI	No	Central African Republic	Central	Nasopharyngeal	rRT-PCR
Obodai, 2014	Cross-sectional Prospective	Urban	Jan/2011-Dec/2012	Children	0-5y	100	SRTI	Yes	Zambia	Southern	Nasopharyngeal	rRT-PCR
Obodai, 2018	Cross-sectional Retrospective	Urban	2003-2004	Children	0-5y	100	SRTI	No	South Africa	Southern	Nasopharyngeal/Tracheal/Broncho-alveolar lavage	rRT-PCR
Oladele, 2019	Cross-sectional Prospective	Urban	Jan/2009-Apr/2011	Children and Adults	na	39.3	BRTI and SRTI	Yes	Democratic Republic of Congo	Central	Not described	rRT-PCR
Ouédraogo Yugbaré, 2016	Cross-sectional Prospective	Urban	2010-2012	Children and Adults	na	29.6	BRTI	Yes	Burkina Faso	Western	Oropharyngeal	rRT-PCR
Ouedraogo, 2014	Cross-sectional Prospective	Urban	Jun/2008-Dec/2009	Children and Adults	na	83.3	BRTI and SRTI	Yes	Zambia	Southern	Nasopharyngeal and Oropharyngeal	rRT-PCR
Sanou, 2018	Cross-sectional Prospective	Urban and Rural	2012-2013	Children and Adults	na	65	BRTI and SRTI	No	Senegal	Western	Not described	rRT-PCR
Tarnagda, 2014	Cross-sectional Prospective	Urban	2008-2014	Children and Adults	na	55.2	BRTI	Yes	Uganda	Eastern	Nasopharyngeal and Oropharyngeal	rRT-PCR
Thiam, 2015	Cross-sectional Prospective	Urban	Jan/2009-Dec/2015	Children and Adults	na	15.3	BRTI and SRTI	Yes	Ethiopia	Eastern	Oropharyngeal	rRT-PCR

Abbreviations: BRTI-Benign respiratory tract infection ; SRTI-Sever respiratory tract infection ; rRT-PCR-Reel-time retro-transcriptase polymersase chain reaction; na-non applicable

Table S4-2 : Characteristics of included studies

Study	Total	IFV	RSV	HMPV	PIV	HRV	EV	AdV	HBoV	HCoV
Kabego, 2018	108	1	20		42			10		
Kenmoe, 2016	6264	34	781	359	591			1361		
Lekana-Douki, 2014	834		240							
Nakouné, 2013	246	28	1	7	77	7	7	12	6	
Njouom, 2010	369	70	124	19	155	81		170		68
Njouom, 2012	8723	158	2268			3228	872	2268		
Serengbe, 2015	7052	58	321	124	208	1267	119	613		
Tamfum, 2012	1410	605	469	72	107	426	102	165		
Ahmed, 2012	1427	768	158	56	99	311	257	363	79	38
Agoti, 2014	232		7		13	40				6
Balinandi, 2013	5898	119	437							
Emukule, 2014	334	348	27			14		2		
Enan, 2013	5338	66	316	60	98	152	147	215		
Feikin, 2012	2172	319	316	14	124	152	38	215	37	
Feikin, 2013	1216	90	92	60	122	147	109	113		
Ho, 2018	204	163	17	15	8			33		
Hoffman, 2012	175	20	54	12	23	76		30		49
Katz, 2014	1126	6674								
Kim, 2011	295	321	24	31	13	38	1	9	1	15
Mmbaga,2012	55	140	2				1	2	2	
Mohamed, 2015	8209	66	28		36			9		
Nyatanyi, 2012	419	377	28		36			9		
Nyawanda, 2016	146		31		23	24	2	7	2	5
Onyango, 2012	5074	71								
Owor, 2016	38775									
Peterson, 2016	347	242	46	14	23	40	40	95	37	20
Razanajatovo, 2011	2331	97	328	201				679		

Razanajatovo, 2018	128	222	18		4			13		
Wabwire-Mangen, 2016	160	687	51	40	32	201	18	105		
Woyessa, 2018	1041	988	119							
Cohen, 2015 (1)	569		244		33					
Cohen, 2015 (2)	532	621								
Cohen, 2016	2128	56								
Famoroti, 2018	955	73								
Ghani, 2012	2322	26	1330			575		347		
Lonngren, 2014	1778	39								
Mazur, 2017	419	30	16	14	24					
Moyes, 2013	4293		1157							
Moyes, 2017	7796		329							
Nunes, 2014	329	61	10		11					
O'Callaghan-Gordo, 2011 (1)	9176	39	266							
O'Callaghan-Gordo, 2011 (2)	6381	51						1967		
Pale, 2017	238	11								
Pretorious, 2012	561	703	32	28	42	100	33			30
Pretorious, 2013	1460		237	107	58	446		29	174	156
Pretorious, 2016	1916	688								
Simusika, 2015	3810		470							
Smuts, 2008	53	9	32							
Theo, 2012	552	57	127							
Adiku, 2015	475		50	39	31	196	18	102		
Akinloye, 2011	333	17		22	16	86	9	47		
Dia, 2014 (1)	120	317	41							
Dia, 2014 (2)	1404	67								
Fall, 2016	208	83	24							
Howie, 2014	153		24	1		88	38			
Jallow, 2019	3320	19		160						

Johnson, 2008	450	19	113							
Kadjo, 2012	1505	969	164	25	246	301	50	136	102	130
Kwofie, 2012	8173	1	1169	303	528	2034	515	1063		
Lagare, 2015	13432	40	2182							
Mackenzie, 2019	5743	200	616	227	293	1731	208	813		
Maïnassara, 2015	313	200	65	3	16	77		19	2	25
Maman, 2014	876	236	348		31	125		77	40	36
Niang, 2012	924	1233								
Niang, 2017	361		21		34	16	16	10	5	
Obodai, 2014	297		24	11	13	34		27		
Obodai, 2018	1055		42	28	42			65		9
Oladele, 2019	4156									
Ouédraogo Yugbaré, 2016	881									
Ouedraogo, 2014	1234	13								
Sanou, 2018	2419	124		95	126	649	584	633		61
Tarnagda, 2014	6628	58								
Thiam, 2015	4799	847								

Abbreviations : IFV-Influenza virus, RSV-Respiratory syncytial virus, HMPV-Human metapneumovirus, HPIV-Human parainfluenza virus, HRV-Human rhinovirus, EV-Enterovirus, AdV-Adenovirus, HBoV-Human Bocavirus, HCoV-Human Coronavirus.
