# Supplementary material for: Viral etiology of acute respiratory infections in Sub-Saharan Africa during the pre-COVID-19 period (2006–2019): a systematic review and meta-analysis
Source: BMC Infect Dis. 2025 Nov 23;25:1799. doi: 10.1186/s12879-025-12122-8 (PMC12750592; doi:10.1186/s12879-025-12122-8)
Supplement: Supplementary file 1 — Supplementary Material 1 [file 12879_2025_12122_MOESM1_ESM.zip › Table S5.pdf]

Table S5: Overall prevalence and comparison statistics of Human Respiratory Viruses Prevalence among ARI patients in Sub-Sahara Africa

	N studies	N participants	Cases (n)	Prevalence, % (95% confidence interval)	I^2	Cochrane's Q statistics	heterogeneity p-value	p-value
**Influenza**								
Overall	57	153612	19639	13.94 [11.27;16.62]	99.7%	18228.80	<0.0001	
Age group						12.27	<0.0001	0.0022
Children (0-15y)	25	177312	15629	21.41 [07.30; 35.51]	95.2%	1320.09		
Adults	2	16695	1235	09.12 [05.90; 12.34]	98.2%	20.84		
Children and Adults	20	129141	17458	17.42 [13.66; 21.17]	99.8%	16678.79		
Age group						7.64	<0.0001	0.0219
Under 5 years	19	177312	15629	16.63 [08.62; 24.64]	96.3%	672.52		
5 years and above	4	92267	11238	08.98 [04.79; 13.17]	97.3%	80.85		
All	21	269579	26867	08.98 [04.79; 13.17]	99.8%	17015.18		
Clinical presentation						6.22	<0.0001	0.0445
SRTI	22	167974	15493	10.32 [06.52; 14.13]	98.8%	4599.73		
BRTI	14	192384	17866	18.90 [13.13; 24.67]	99.7%	2649.05		
BRTI and SRTI	21	76407	12087	14.44 [10.14; 18.74]	99.2%	1801.65		
Settings						1.21	<0.0001	0.7512
Urban	24	197183	18854	13.03 [09.23; 16.82]	99.1%	2605.27		
Rural	9	121043	12265	17.35 [07.78; 26.91]	98.7%	598.15		
Urban and Rural	15	61975	12389	14.68 [09.59; 19.77]	99.7%	4382.02		
Unclear/Not described	9	17853	704	11.80 [05.91; 17.69]	98.9%	732.67		
Africa region						25.58	<0.0001	< 0.0001
Eastern	19	204527	19639	17.89 [13.48; 22.30]	99.1%	1934.74		
Western	17	186002	17196	15.05 [09.37; 20.73]	99.7%	5225.6		
Southern	14	174245	16213	06.54 [04.22; 08.86]	98.8%	1070.50		
Central	7	131060	13811	15.14 [07.45; 22.82]	98.5%	399.45		

**RSV**								
Overall	53	119634	15456	16.05[12.51 ; 19.59]	99.7%	15760.46	<0.0001	
Age group						46.40	<0.0001	< 0.0001
Children (0-15y)	32	38865	8294	19.39 [14.42; 24.36]	99.4%	5250.53		
Adults	2	8047	336	04.12 [03.47; 04.77]	9.2%	1.10		
Children and Adults	19	72633	6826	11.91 [07.45; 16.36]	99.7%	6134.36		
Age group						46.85	<0.0001	<0.0001
Under 5 years	27	30372	7194	20.01 [14.59; 25.44]	99.5%	4828.28		
5 years and above	4	15518	692	04.33 [04.01; 04.65]	0.0%	2.70		
All	31	45890	7886	13.57 [08.90; 18.23]	99.7%	6999.04		
Clinical presentation						8.20	<0.0001	0.0166
SRTI	30	70438	11822	20.19 [15.18; 25.20]	99.6%	954.03		
BRTI	10	27000	1142	10.10 [04.05; 16.16]	99.1%	1012.17		
BRTI and SRTI	13	22088	2472	11.22 [05.38; 17.05]	98.7%	6715.77		
Settings						17.36	<0.0001	0.0006
Urban	20	20032	2073	18.76 [13.03; 24.49]	98.7%	1512.55		
Rural	7	12227	1100	08.18 [05.91; 10.46]	93.9%	98.54		
Urban and Rural	17	69414	11242	18.35 [11.67; 25.04]	99.7%	6039.17		
Unclear/Not described	9	17853	1021	11.99 [02.34; 21.64]	99.3%	1165.00		
Africa region						6.42	<0.0001	0.0930
Eastern	14	181773	15349	15.34 [09.60; 21.07]	98.3%	767.80		
Western	16	148069	10712	14.53 [07.32; 21.75]	98.6%	1074.42		
Southern	17	176697	14290	20.27 [13.27; 27.27]	99.7%	6335.20		
Central	6	131060	9660	09.61 [04.72; 14.50]	92.8%	69.57		
**HMPV**								
Overall	29	55232	2147	04.66 [03.53; 05.78]	96.6%	816.79	< 0.0001	
Age group						0.95	<0.0001	0.3287
Children (0-15y)	16	19146	810	05.37 [03.21; 07.53]	96.0%	373.56		

Adults	--	--	--	--	--	--		
Children and Adults	13	36086	1337	04.14 [02.91; 05.36]	97.3%	442.57		
Age group						6.70	<0.0001	0.0350
Under 5 years	13	11551	544	06.02 [03.26; 08.79]	96.3%	326.68		
5 years and above	2	7490	157	02.31 [00.83; 03.79]	68.3%	3.15		
All	15	19041	701	04.16 [03.03; 05.29]	97.0%	437.04		
Clinical presentation						4.36	<0.0001	0.1129
SRTI	16	33830	1199	04.83 [02.85; 06.81]	96.3%	408.76		
BRTI	5	8027	185	03.07 [01.28; 04.87]	92.6%	54.23		
BRTI and SRTI	9	13413	801	0.5.84 [02.30; 09.38]	96.6%	67.10		
Settings						12.01	<0.0001	0.0073
Urban	10	10991	349	03.08 [01.67; 04.49]	95.5%	201.4		
Rural	5	2542	186	07.18 [05.28; 09.08]	69.3%	13.01		
Urban and Rural	9	33209	1304	05.84 [02.30; 09.38]	96.6%	232.85		
Unclear/Not described	5	8509	294	04.45 [01.63; 07.26]	97.6%	169.54		
Africa region						0.52	<0.0001	0.9138
Eastern	10	16236	841	05.09 [03.33; 06.85]	95.5%	199.54		
Western	6	9743	1788	05.79 [00.00; 12.51]	95.7%	116.22		
Southern	11	28345	1667	04.36 [02.93; 05.79]	96.8%	308.23		
Central	2	908	182	04.58 [03.22; 05.94]	0.0%	0.47		
**HPIV**								
Overall	36	60805	3378	08.96 [06.08; 11.83]	98.6%	2465.15	<0.001	
Age group						3.26	<0.0001	0.1957
Children (0-15y)	21	17733	1281	09.91 [06.27; 13.55]	94.2%	343.21		
Adults	1	237	13	05.60 [02.64; 08.56]	--	0.00		
Children and Adults	14	42840	2084	07.78 [02.71; 12.86]	99.2%	1601.0		
Age group						7.24	<0.0001	0.0268
Under 5 years	16	9240	675	10.83 [06.12; 15.55]	92.8%	208.45		

5 years and above	3	7722	264	04.41 [02.38; 06.45]	76.9%	208.45		
All	19	16962	939	08.07 [03.82; 12.32]	99.2%	1958.03		
Clinical presentation						0.13	<0.0001	0.9373
SRTI	18	31888	1857	08.34 [05.05; 11.63]	95.7%	172.31		
BRTI	7	16449	459	09.78 [00.00; 20.24]	98.7%	454.89		
BRTI and SRTI	11	12468	1062	09.17 [04.75; 13.58]	94.2%	172.31		
Settings						4.00	<0.0001	0.2617
Urban	13	9428	843	12.30 [05.49; 19.12]	97.2%	427.79		
Rural	6	2871	220	05.58 [03.50; 07.66]	85.5%	34.47		
Urban and Rural	10	33337	2030	06.83 [04.79; 08.87]	97.5%	323.84		
Unclear/Not described	7	15188	304	08.85 [01.29; 16.41]	97.2%	239.17		
Africa region						6.09	<0.0001	0.1072
Eastern	9	11480	1225	11.05 [03.24; 18.85]	97.7%	346.98		
Western	10	16286	456	11.74 [03.84; 19.64]	98.2%	488.19		
Southern	12	28914	1476	05/38 [04.43; 06.33]	93.0%	158.02		
Central	5	1744	133	07.94 [04.49; 11.38]	83.3%	23.96		
**HRV**								
Overall	30	54633	12508	21.26 [16.76; 25.75]	99.6%	7328.23	<0.0001	
Age group						3.17	<0.0001	0.2053
Children (0-15y)	18	26323	7877	24.08 [17.30; 30.85]	99.4%	2754.65		
Adults	1	232	40	17.24 [12.38; 22.10]	--	0.00		
Children and Adults	11	28078	2652	17.13 [12.29; 21.97]	99.7%	2860.9		
Age group						2.83	<0.0001	0.2429
Under 5 years	15	21956	8821	24.81 [16.85; 32.77]	99.4%	2531.35		
5 years and above	2	7384	23715	24.81 [16.85; 32.77]	0.00%	0.08		
All	17	29340	32536	17.89 [13.23; 22.54]	99.7%	3729.79		
Clinical presentation						10.31	<0.0001	0.0058
SRTI	17	41079	10972	26.65 [20.59; 32.71]	99.3%	2261.60		

BRTI	7	8593	794	15.62 [09.03; 22.20]	98.9%	530.84		
BRTI and SRTI	6	4683	881	12.53 [05.42; 19.64]	98.7%	392.19		
Settings						3.85	<0.0001	0.2776
Urban	11	7360	1353	19.74 [11.36; 28.13]	98.4%	608.31		
Rural	4	2338	486	22.93 [09.52; 36.35]	98.1%	160.88		
Urban and Rural	10	19632	4361	26.04 [22.27; 29.80]	99.0%	868.84		
Unclear/Not described	5	5672	166	13.84 [00.00; 28.87]	97.3%	150.60		
Africa region						12.50	<0.0001	0.0059
Eastern	7	4927	802	15.55 [10.41; 20.69]	96.0%	150.62		
Western	7	9994	1467	22.39 [08.73; 36.05]	99.5%	1158.75		
Southern	12	38335	10251	26.88 [20.71; 33.05]	99.5%	2064.89		
Central	4	1415	180	12.32 [06.19; 18.46]	94.5%	54.90		
**Enterovirus**								
Overall	22	48310	3184	07.01 [04.20; 09.81]	98.8%	1713.52	<0.001	
Age group						1.4	<0.0001	0.2366
Children (0-15y)	15	25352	4648	05.59 [02.93; 08.26]	97.8%	631.40		
Adults	--	--	--	--	--	--		
Children and Adults	7	26186	1764	09.65 [03.48; 15.81]	99.4%	1075.87		
Age group						14.94	<0.0001	0.0006
Under 5 years	12	17757	4350	05.56 [02.29; 08.84]	--	0.00		
5 years and above	1	7052	119	01.69 [01.39; 01.99]	98.2%	0.00		
All	13	24809	4469	0.0556 [02.29; 08.84]	99.1%	852.90		
Clinical presentation						0.26	<0.0001	0.8801
SRTI	13	39529	5256	06.42 [03.47; 09.37]	98.5%	794.03		
BRTI	3	7426	537	08.85 [00.00; 17.96]	99.1%	220.20		
BRTI and SRTI	6	4683	719	07.01 [00.00;14.16]	99.3%	698.31		
Settings						9.41	<0.0001	0.0243
Urban	6	4784	284	07.43 [00.70; 14.15]	97.0%	101.09		

Rural	4	2419	2419	03.91 [00.32; 07.51]	94.0%	83.46		
Urban and Rural	10	35723	2709	08.98 [04.48; 13.49]	99.4%	1398.92		
Unclear/Not described	2	5584	154	02.76 [02.33; 03.19]	0.0%	0.01		
Africa region						3.72	<0.0001	0.2934
Eastern	3	3116	260	04.16 [00.00; 09.09]	98.1%	104.26		
Western	7	9898	1152	12.08 [04.58; 19.59]	99.2%	791.71		
Southern	8	34181	1981	04.64 [02.57; 06.71]	99.0%	725.23		
Central	4	1515	191	05/65 [01.63; 09.66]	89.8%	29.29		
**AdV**								
Overall	37	78841	11833	14.35 [10.13; 18.57]	99.7%	0.0169	<0.0001	
Age group						0.58	<0.0001	0.4466
Children (0-15y)	22	27827	4506	12.98 [07.43; 18.54]	99.1%	2389.51		
Adults	--	--	--	--	--	--		
Children and Adults	15			16.33 [09.74; 22.92]	99.8%	9138.22		
Age group						8.44	0.0147	0.0147
Under 5 years	19	19285	3407	12.40 [06.12; 18.69]	99.2%	8.44		
5 years and above	1	7052	613	08.69 [08.04; 09.35]	--	0.00		
All	20	26337	4020	06.85 [10.92; 22.78]	99.8%	9676.16		
Clinical presentation						0.17	0.0147	0.9192
SRTI	21	42880	6231	14.32 [08.77; 19.86]	99.1%	2301.66		
BRTI	7	22390	2764	16.08 [02.81; 29.34]	99.8%	3811.65		
BRTI and SRTI	9	13482	2857	13.13 [06.27; 19.98]	99.2%	1017.27		
Settings						4.45	<0.0001	0.2165
Urban	13	9428	869	20.79 [12.53; 29.05]	97.8%	541.94		
Rural	5	2542	323	12.64 [06.42; 18.86]	95.7%	94.11		
Urban and Rural	13	50257	9713	20.79 [12.53; 29.05]	99.5%	2193.04		
Unclear/Not described	6	16633	947	09.23 [00.00; 18.47]	99.6%	1200.49		
Africa region						0.54	<0.0001	0.9095

Eastern	10	13707	939	14.90 [06.35; 23.44]	99.5%	1673.74		
Western	11	24890	3338	16.45 [05.08; 27.82]	99.8%	4661.52		
Southern	13	39390	5784	12.86 [09.39; 16.33]	99.4%	1864.83		
Central	3	873	131	11.54 [00.00; 26.90]	97.9%	93.68		
**HBoV**								
Overall	12	9203	487	04.17 [01.99; 06.34]	96.3%	0.0014	0.0001	
Age group						0.16	<0.0001	0.6854
Children (0-15y)	9	6587	366	04.40 [01.56; 07/23]	96.7%	243.50		
Adults	--	--	--	--	--	--		
Children and Adults	3			03.55 [00.58; 06.52]	96.0%	49.71		
Age group						1.04	<0.0001	0.3083
Under 5 years	7	4754	246	03.21 [0.019; .0.624]	96.3%	162.73		
5 years and above	--	--	--	--	--	--		
All	5	4515	323	05.45 [002.40; 08.50]	95.9%	96.93		
Clinical presentation						10.11	<0.0001	0.0064
SRTI	6	42880	6221	06.56 [03.26; 09.85]	97.3%	186.93		
BRTI	2	22390	2764	03.07 [00.00; 07.87]	97.6%	42.12		
BRTI and SRTI	4	2860	81	01.12 [00.21; 02.03]	49.0%	5.89		
Settings						60.82	<0.0001	< 0.0001
Urban	8	9428	869	04.78 [01.74; 07.82]	96.9%	228.13		
Rural	1	2542	323	00.34 [00.00; 01.00]	--	0.00		
Urban and Rural	2	4407	129	05.43 [04.28; 06.59]	0.0%	0.54		
Unclear/Not described	1	407	55	02.44 [00.51; 04.37]	--	0.00		
Africa region						0.65	<0.0001	0.8852
Eastern	4	3008	164	03.04 [00.00; 06.11]	97.0%	99.62		
Western	3	1747	106	04.05 [01.74; 06.36]	72.8%	7.36		
Southern	2	3651	230	06.78 [00.00; 16.79]	99.2%	131.05		
Central	3	873	44	04.32 [00.00; 10.23]	93.0%	28.59		

**HCoV**								
Overall	14	11180	648	07.22 [03.77; 10.67]	96.3%	0.0042	< 0.0001	
Age group						5.12	<0.0001	0.0773
Children (0-15y)	7	5002	403	08.55 [02.43; 14.67]	97.8%	270.26		
Adults	1	232	6	02.59 [00.54; 04.63]	--	0.00		
Children and Adults	8	5234	409	06.62 [02.05; 11.19]	93.7%	79.74		
Age group						5.67	<0.0001	0.0588
Under 5 years	5	3150	253	09.28 [00.17; 18.38]	98.0%	196.27		
5 years and above	1	232	6	02.59 [00.54; 04.63]	--	0.00		
All	8	3382	259	06.71 [03.38; 10.04]	94.7%	133.14		
Clinical presentation						4.05	<0.0001	0.1319
SRTI	6	5437	419	09.29 [02.13; 16.45]	98.1%	270.09		
BRTI	5	2921	186	07.22 [01.66; 12.77]	94.4%	71.11		
BRTI and SRTI	3	1048	14	03.32 [01.73; 04.91]	50.3%	4.02		
Settings						5.33	<0.0001	0.1489
Urban	8	6090	468	07.34 [03.72; 10.96]	97.5%	283.33		
Rural	1	295	15	05.08 [02.58; 07.59]	--	0.00		
Urban and Rural	3	1482	81	03.30 [01.73; 04.88]	75.1%	8.03		
Unclear/Not described	2	246	6	15.09 [00.00; 39.99]	98.0%	51.23		
Africa region						0.65	<0.0001	0.0002
Eastern	5	3377	293	08.66 [03.90; 13.43]	93.1%	58.12		
Western	3	4097	124	02.57 [02.09; 03.06]	0.0%	0.07		
Southern	3	2709	233	12.88 [00.00; 28.21]	98.9%	190.38		
Central	3	21502	55	05.08 [03.76; 06.41]	0.0%	1.59		

Abbreviations: RSV-Respiratory syncytial virus, HRV-Human rhinovirus, HPMV-Huamn metapneumovirus, HPIV-Human parainfluenzavirus, HBoV-Human bocavirus, HCoV-Human coronavirus, BRTI-Benign respiratory tract infections, SRTI-Severe respiratory tract unfections, --: Not data
