# Supplementary figures and images for: Viral etiology of acute respiratory infections in Sub-Saharan Africa during the pre-COVID-19 period (2006–2019): a systematic review and meta-analysis
Source: BMC Infect Dis. 2025 Nov 23;25:1799. doi: 10.1186/s12879-025-12122-8 (PMC12750592; doi:10.1186/s12879-025-12122-8)

Fig. S2 : Funnel plot for publication for respiratory viruses in people with ARI in Africa

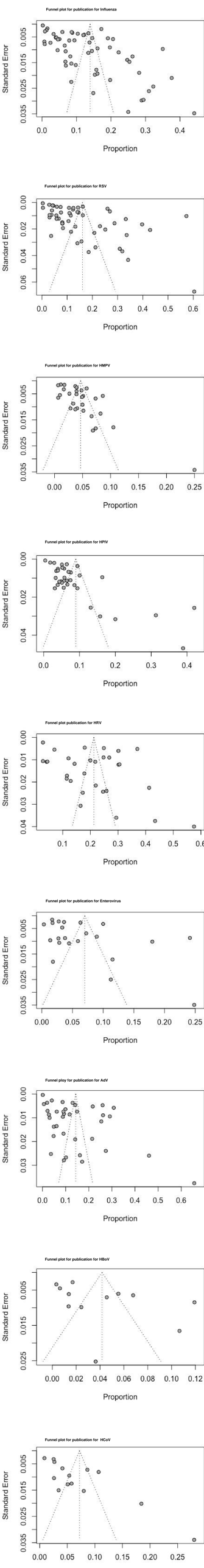

Supplement: Supplementary file 1 — Supplementary Material 1 [file 12879_2025_12122_MOESM1_ESM.zip › Fig. S2.pdf]

Fig. S1 : Forest plot for influenza virus

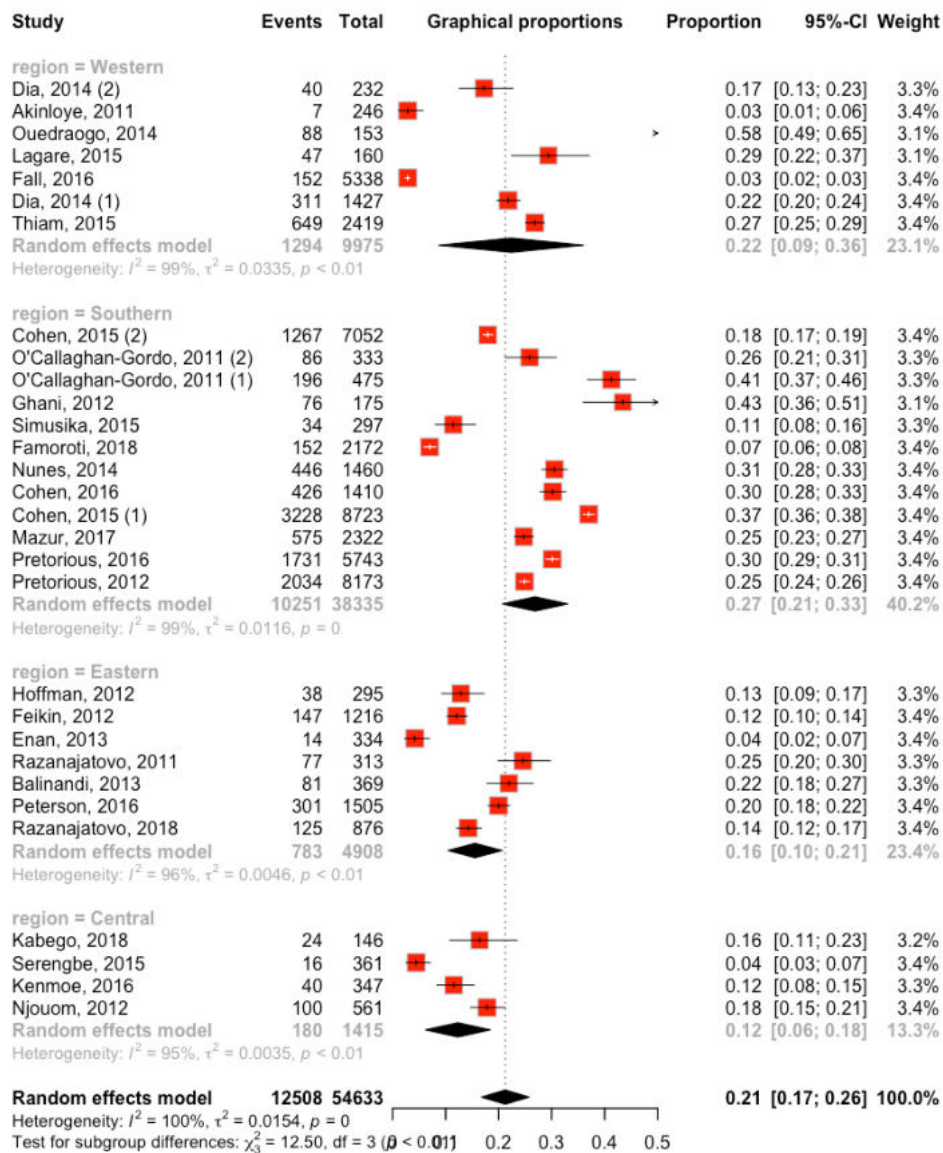

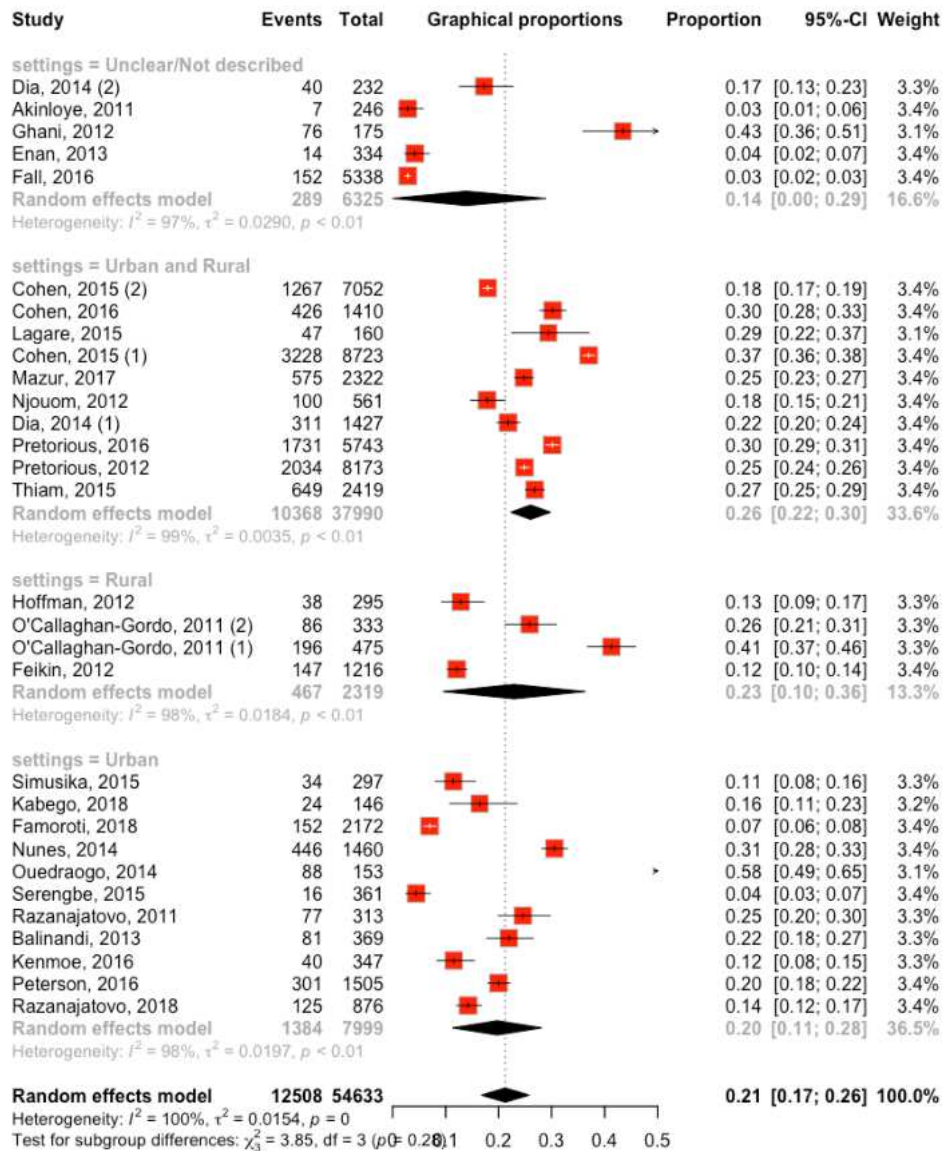

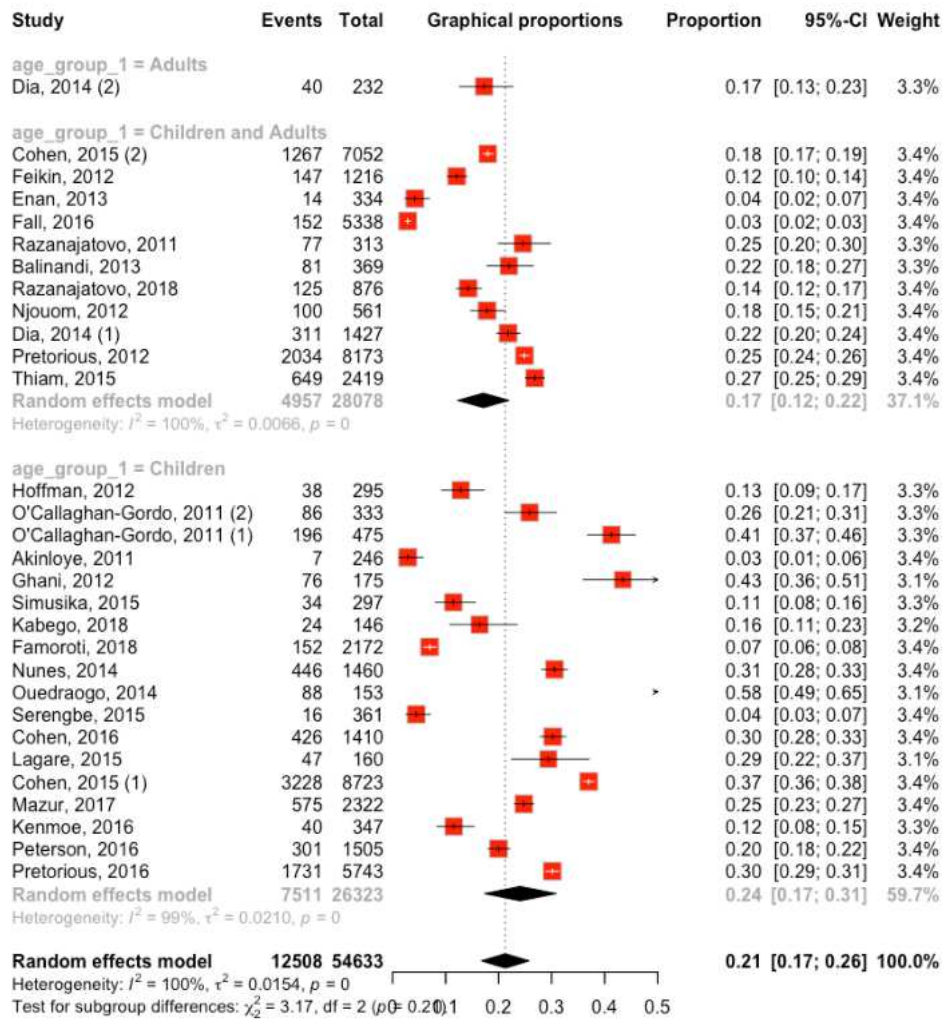

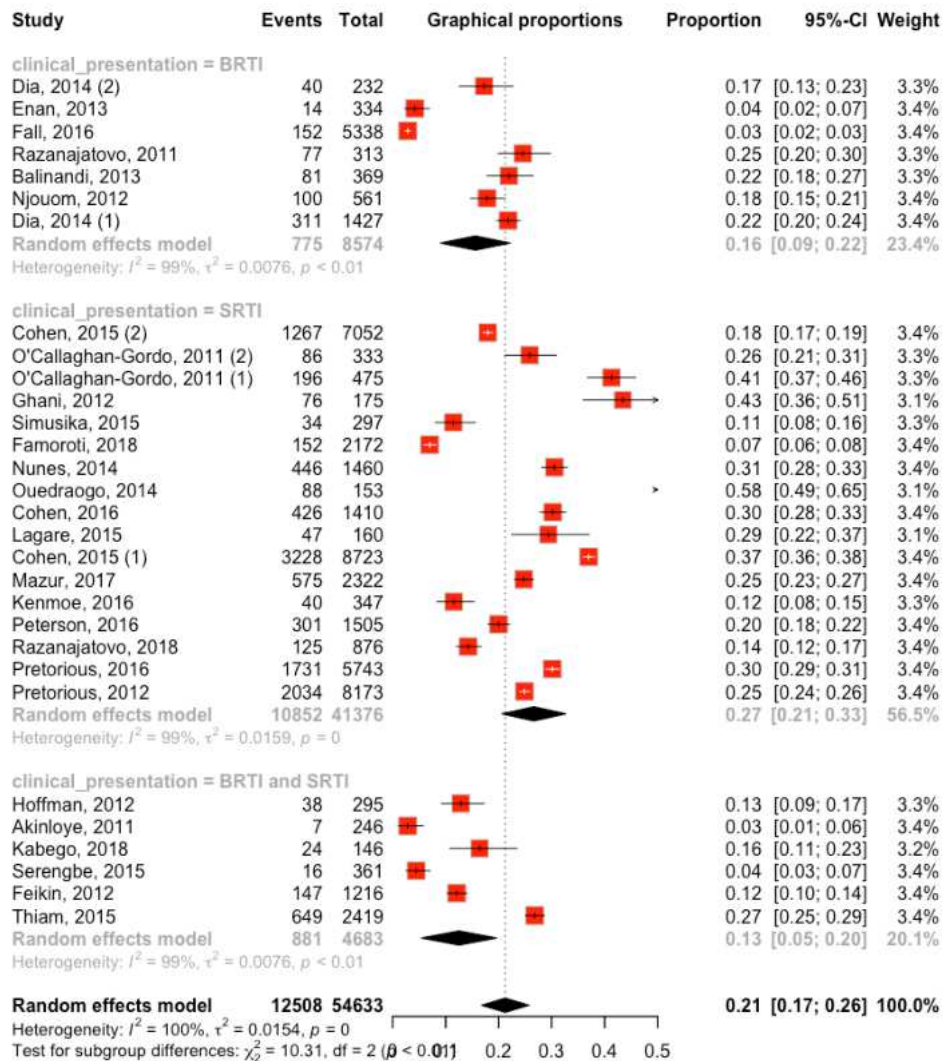

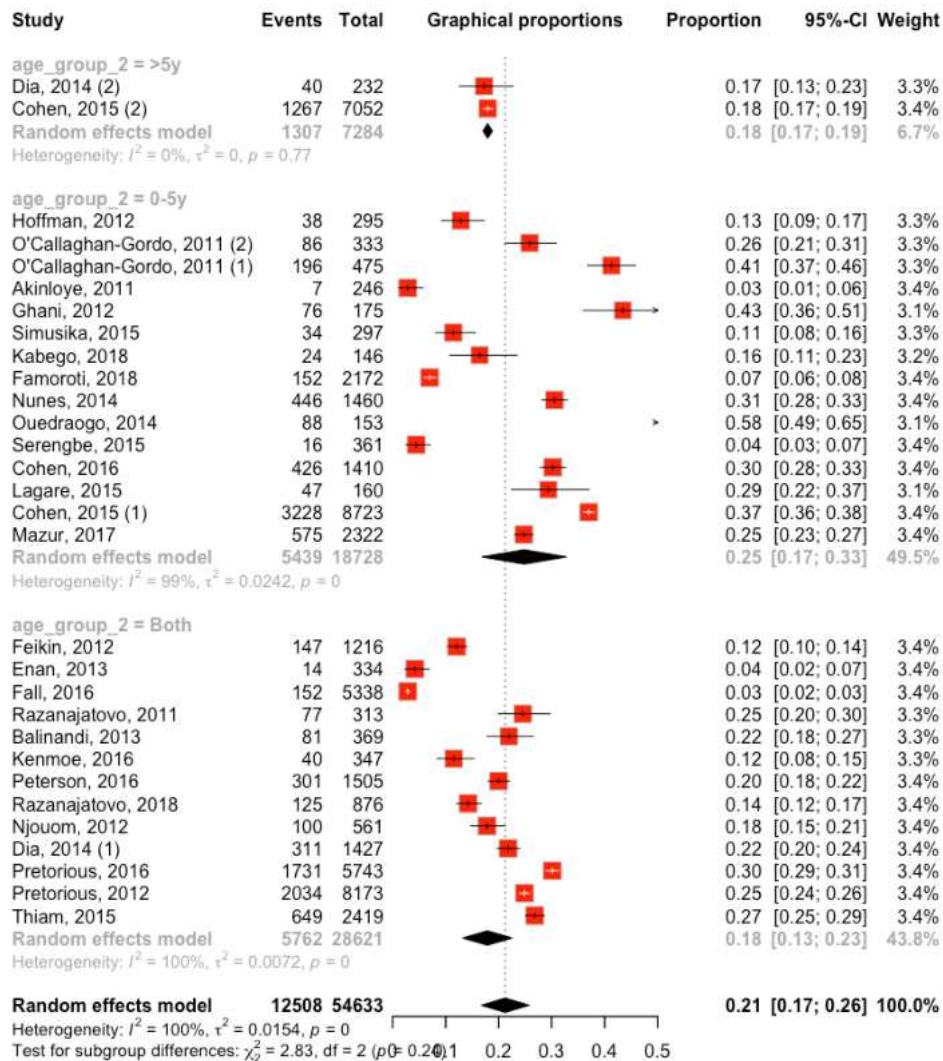

Supplement: Supplementary file 2 — Supplementary Material 2 [file 12879_2025_12122_MOESM2_ESM.zip › Fig.S1.pdf]

Fig. S2 : Forest plot for RSV

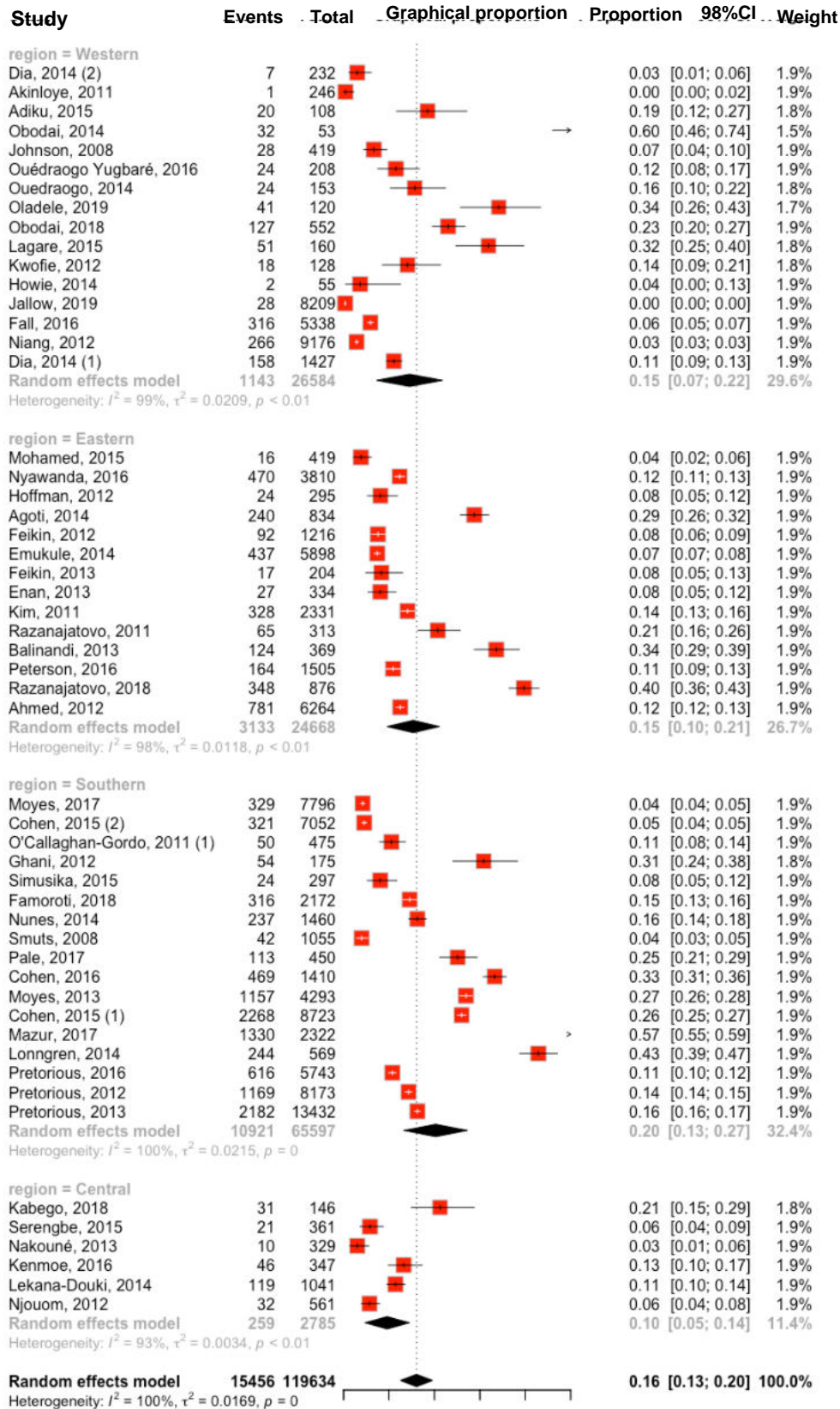

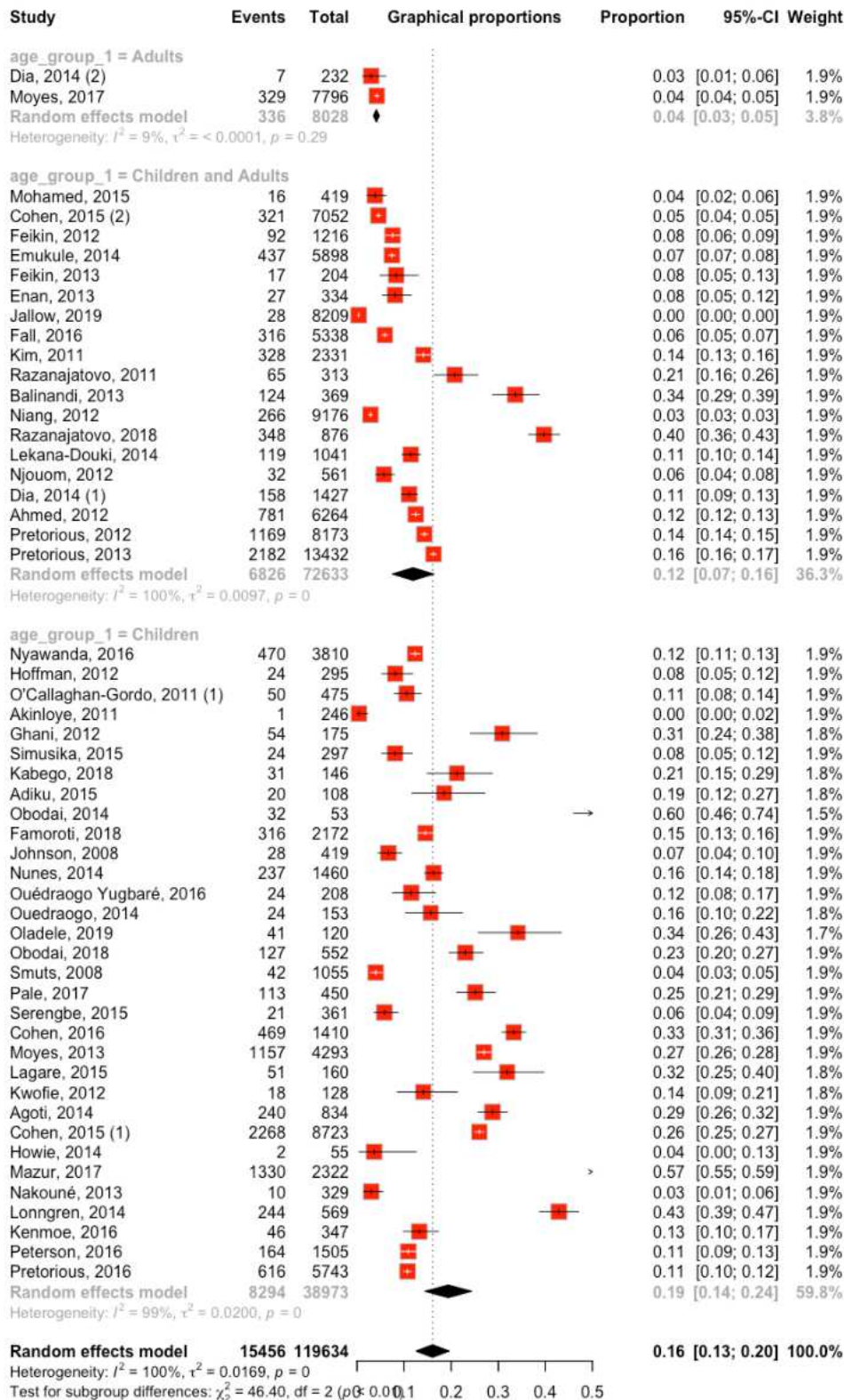

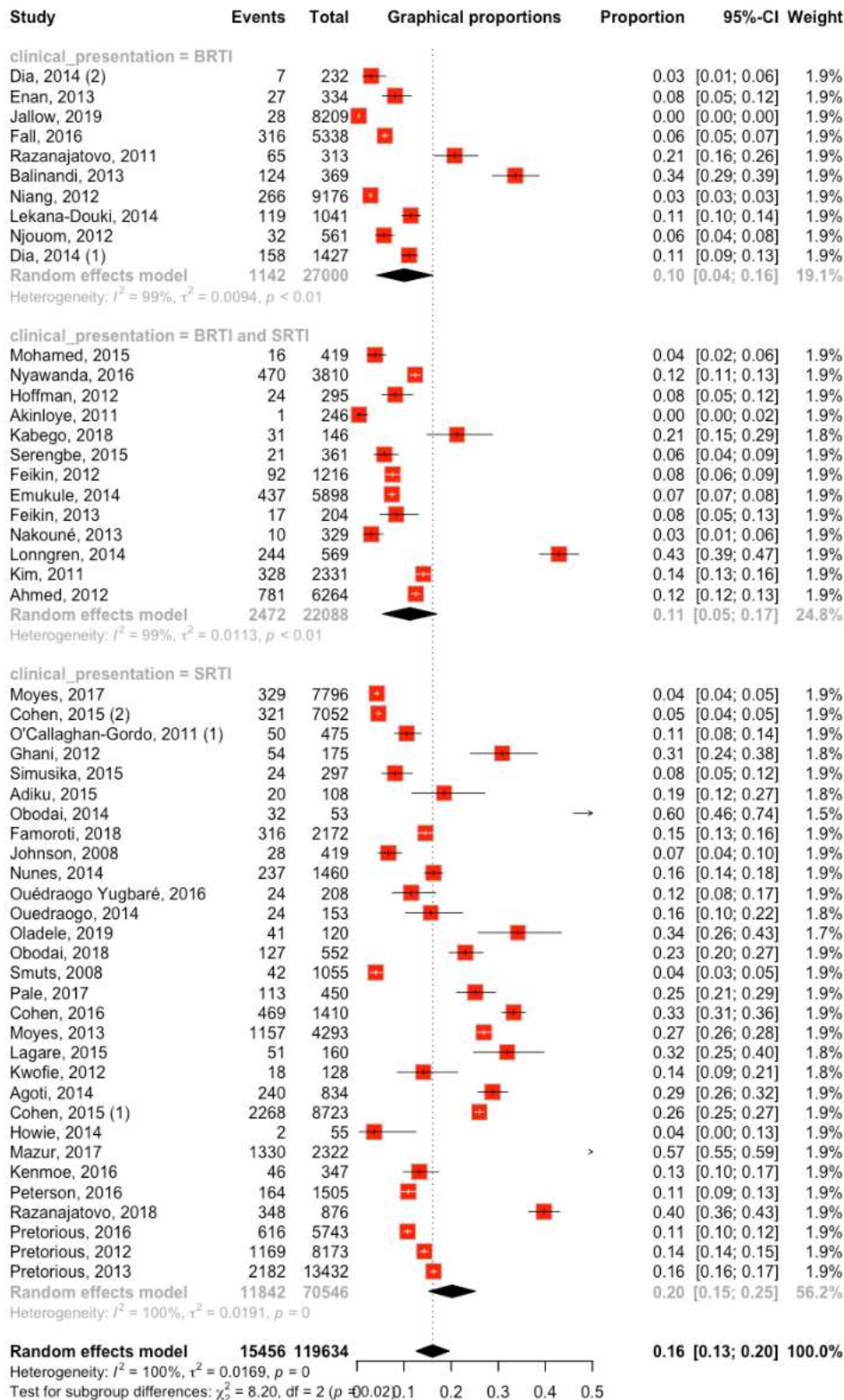

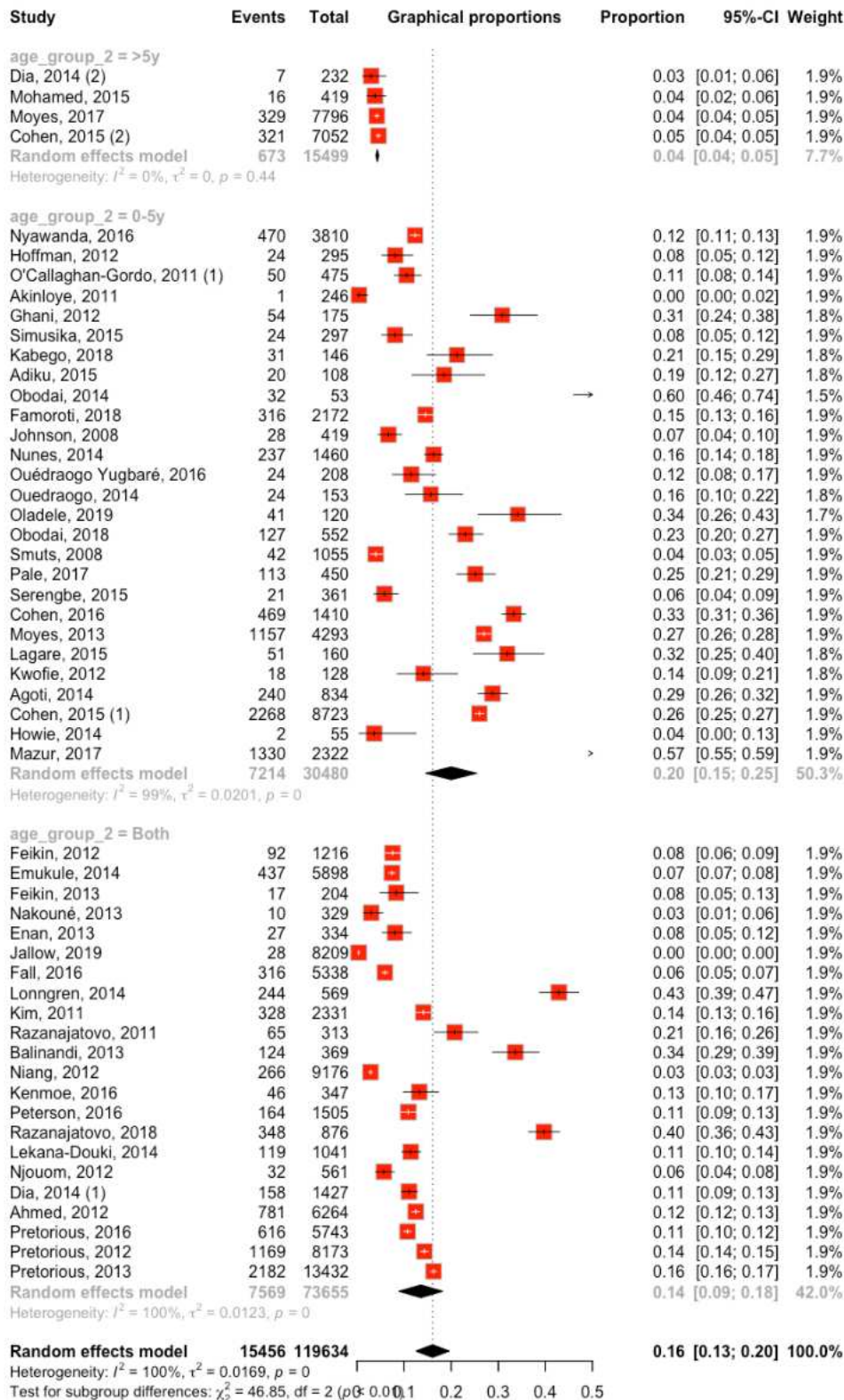

Supplement: Supplementary file 2 — Supplementary Material 2 [file 12879_2025_12122_MOESM2_ESM.zip › Fig.S2.pdf]

**Fig. S3: Forest plot for HPIV**

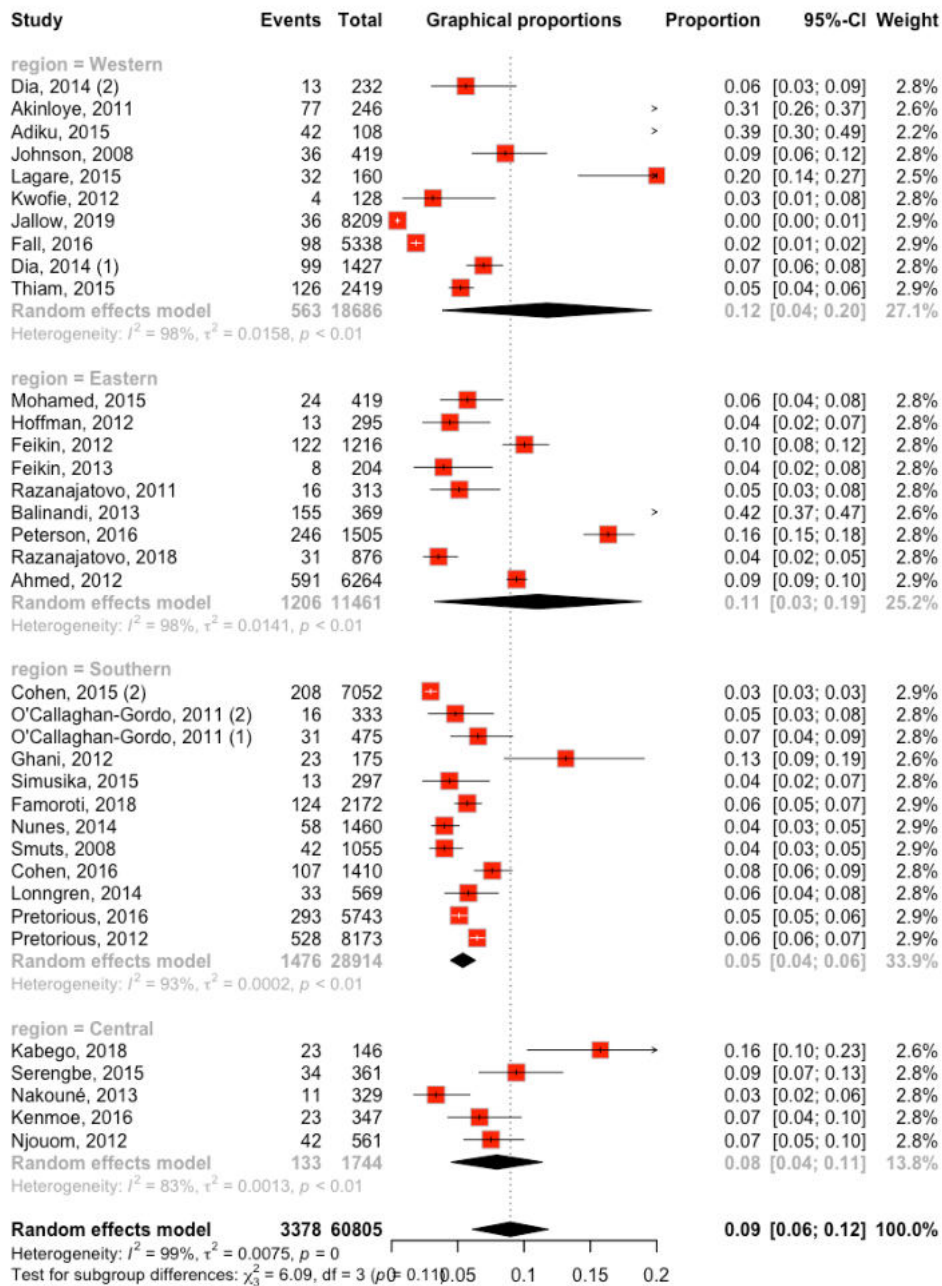

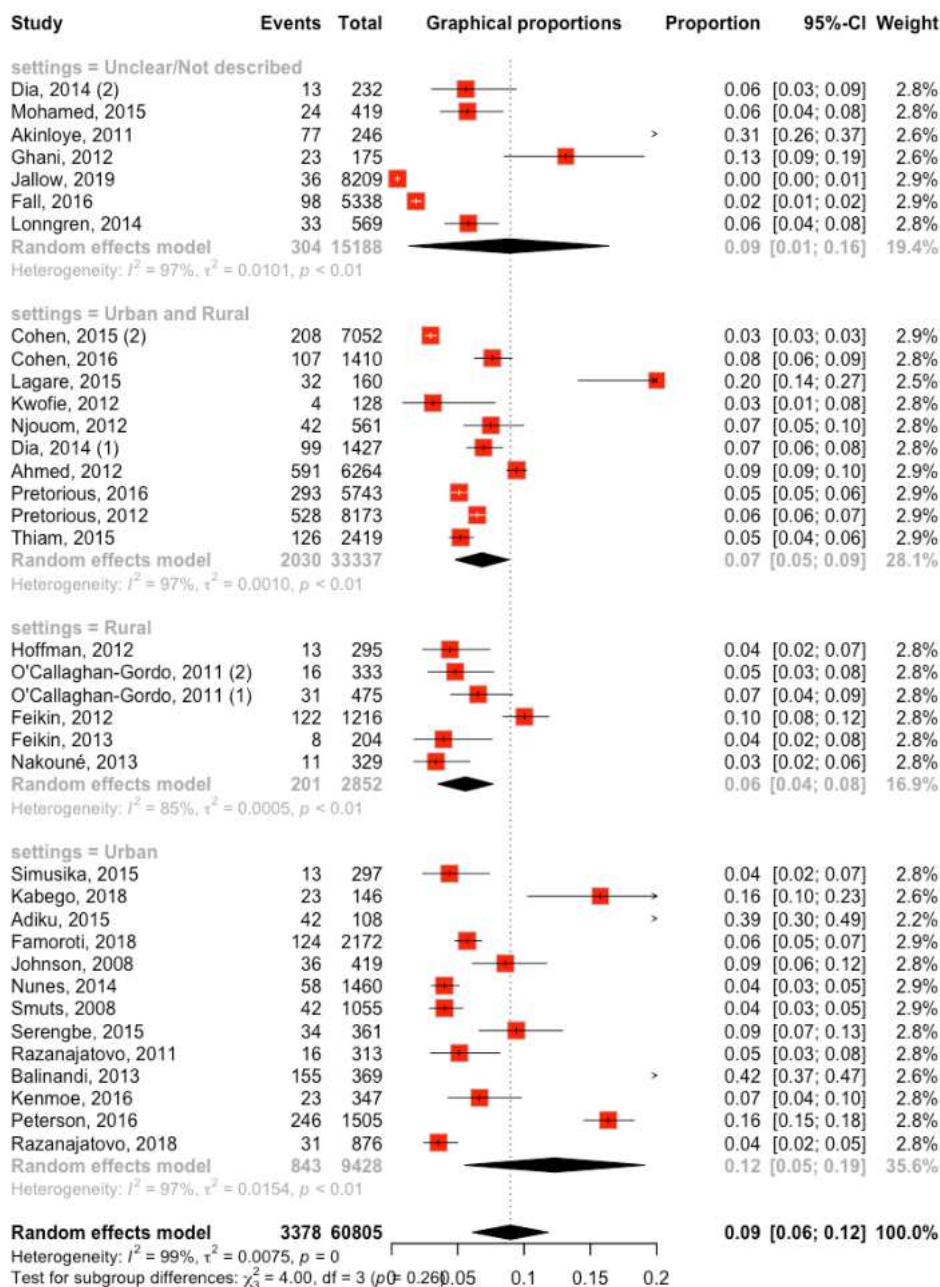

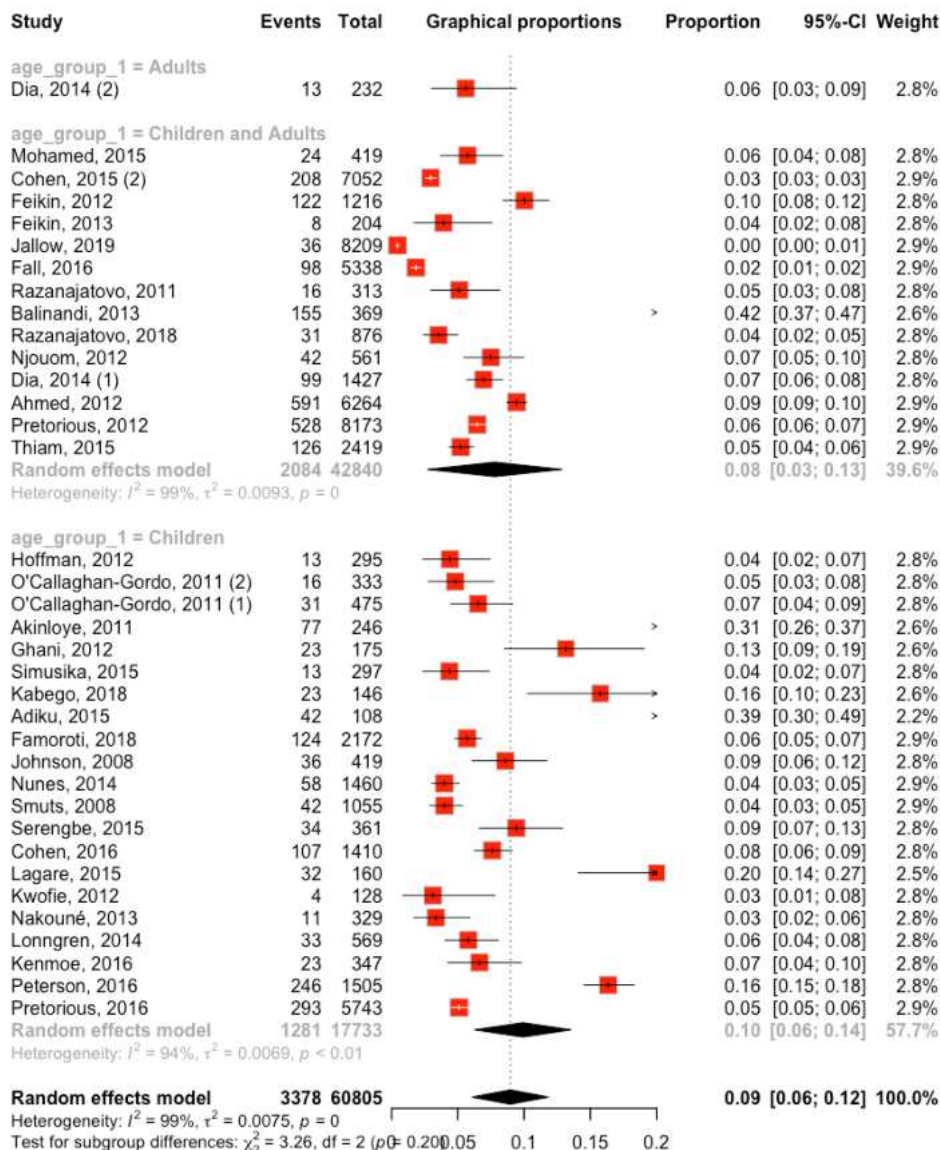

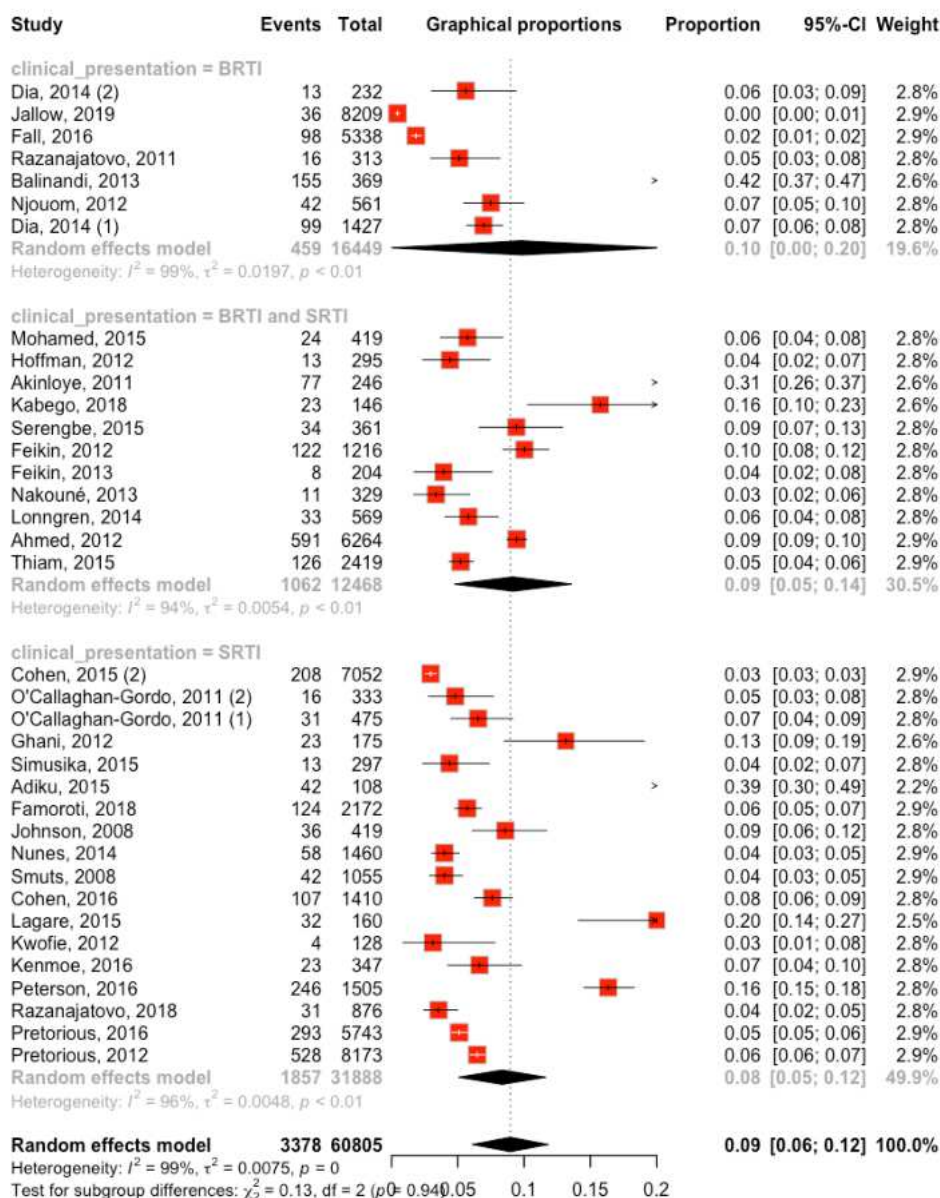

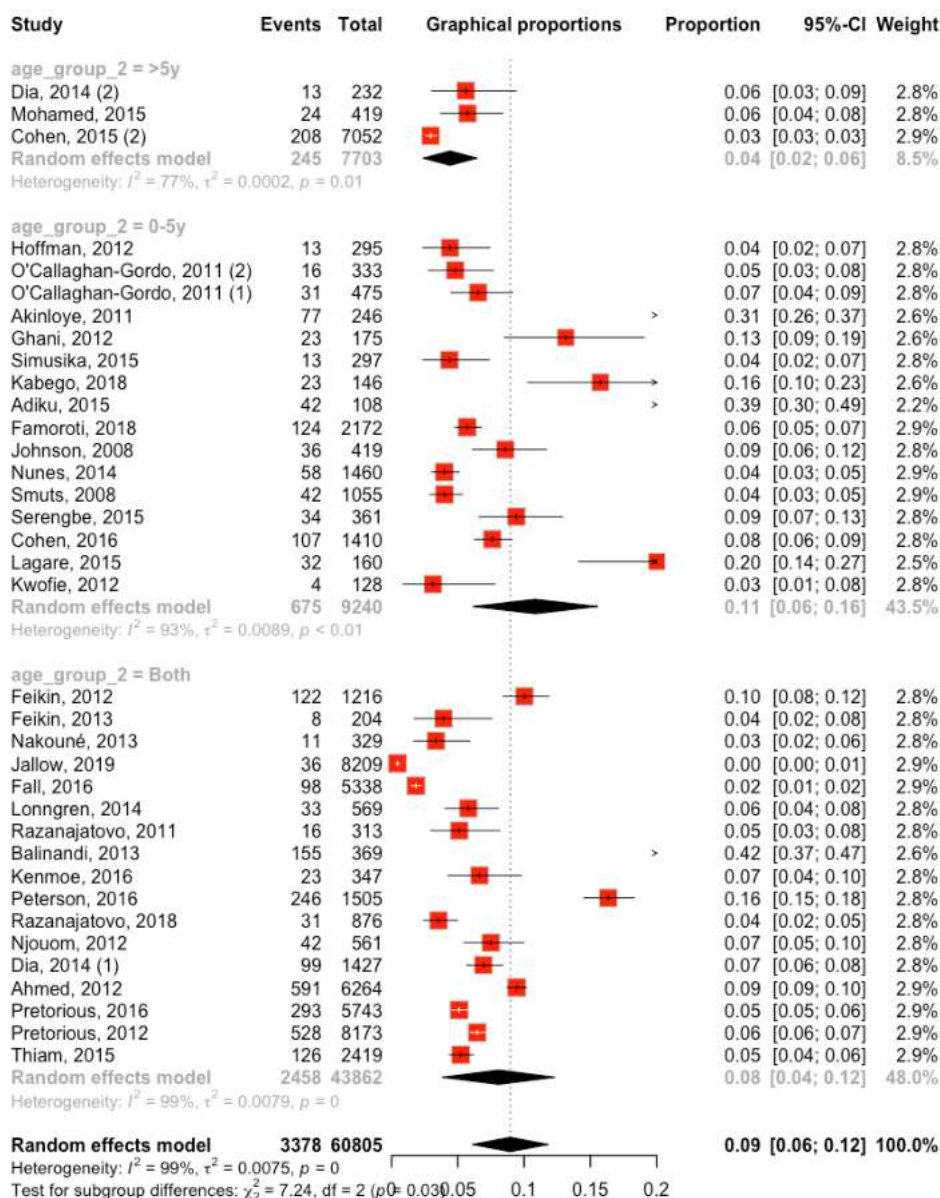

Supplement: Supplementary file 2 — Supplementary Material 2 [file 12879_2025_12122_MOESM2_ESM.zip › Fig.S3.pdf]

Fig. S4: Forest plot for HMPV

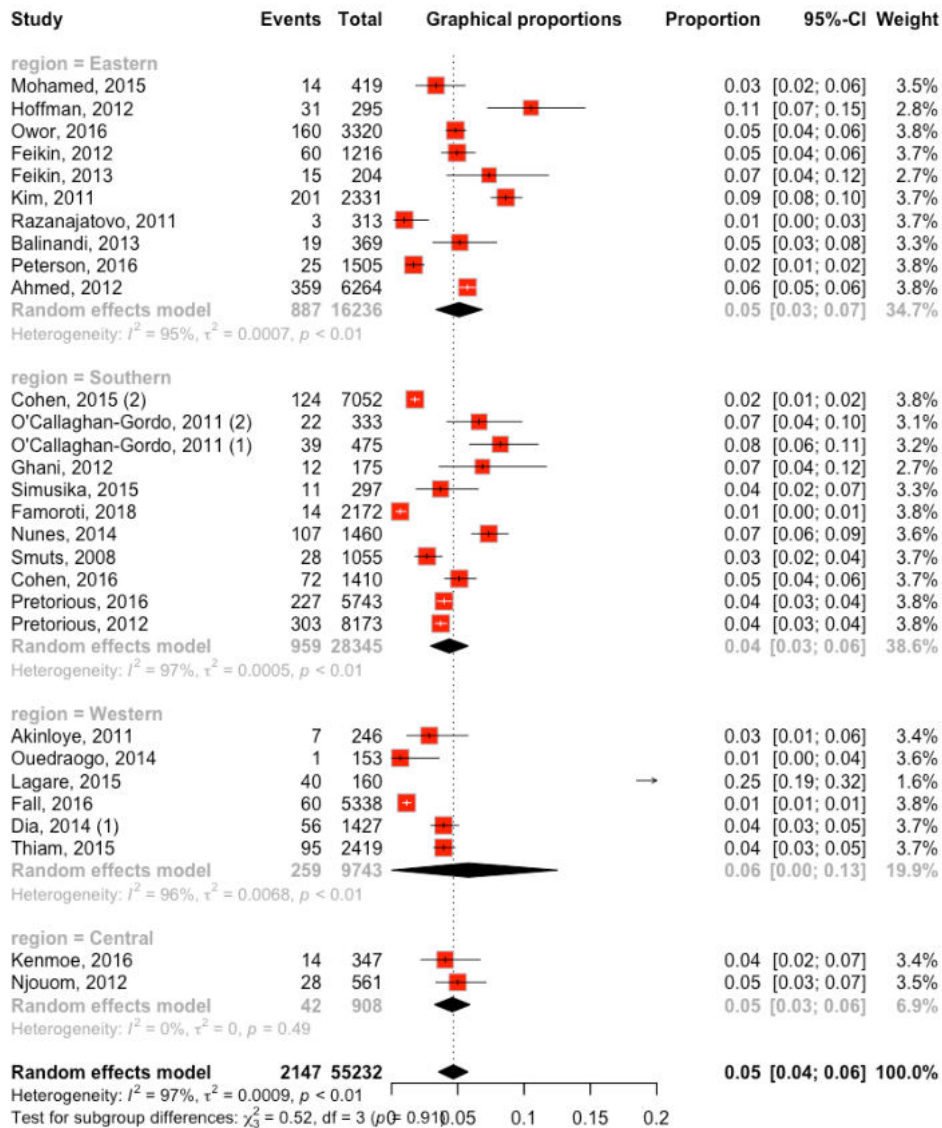

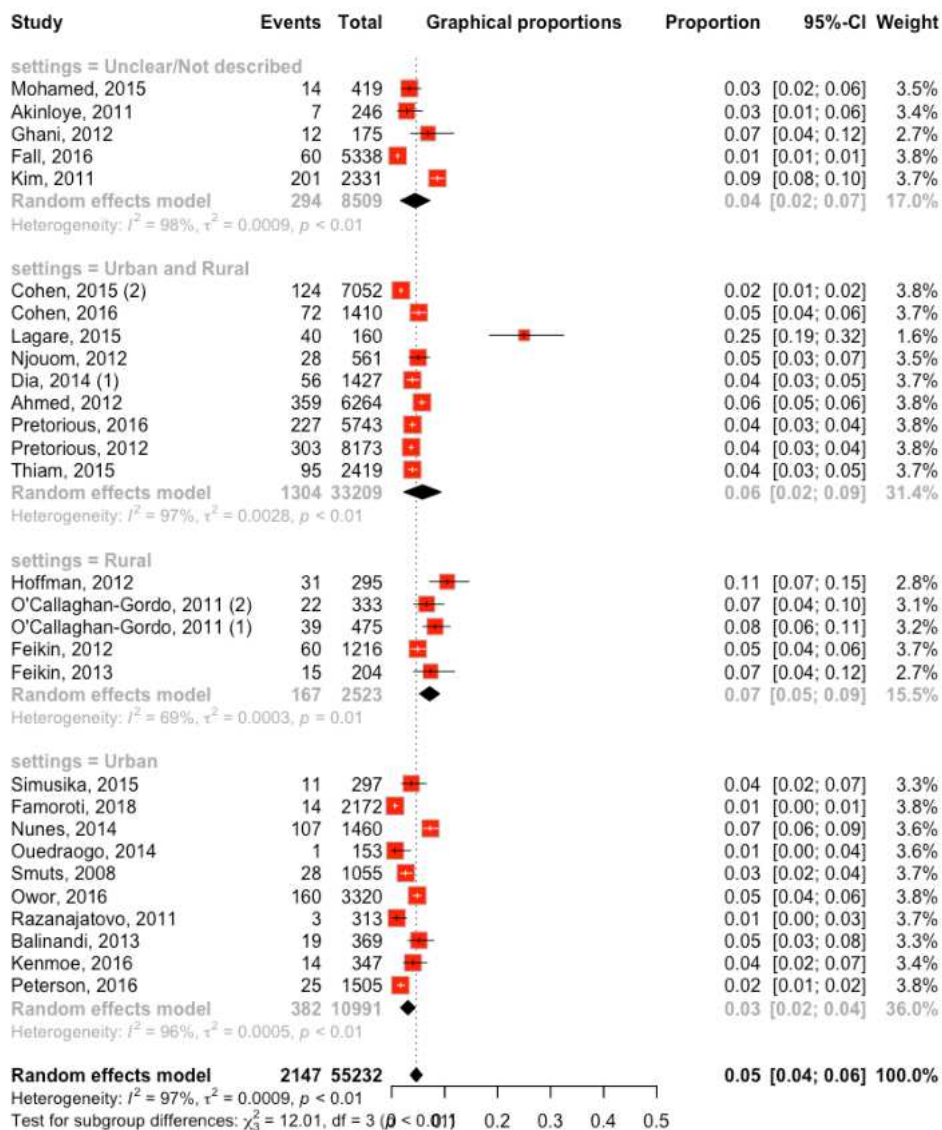

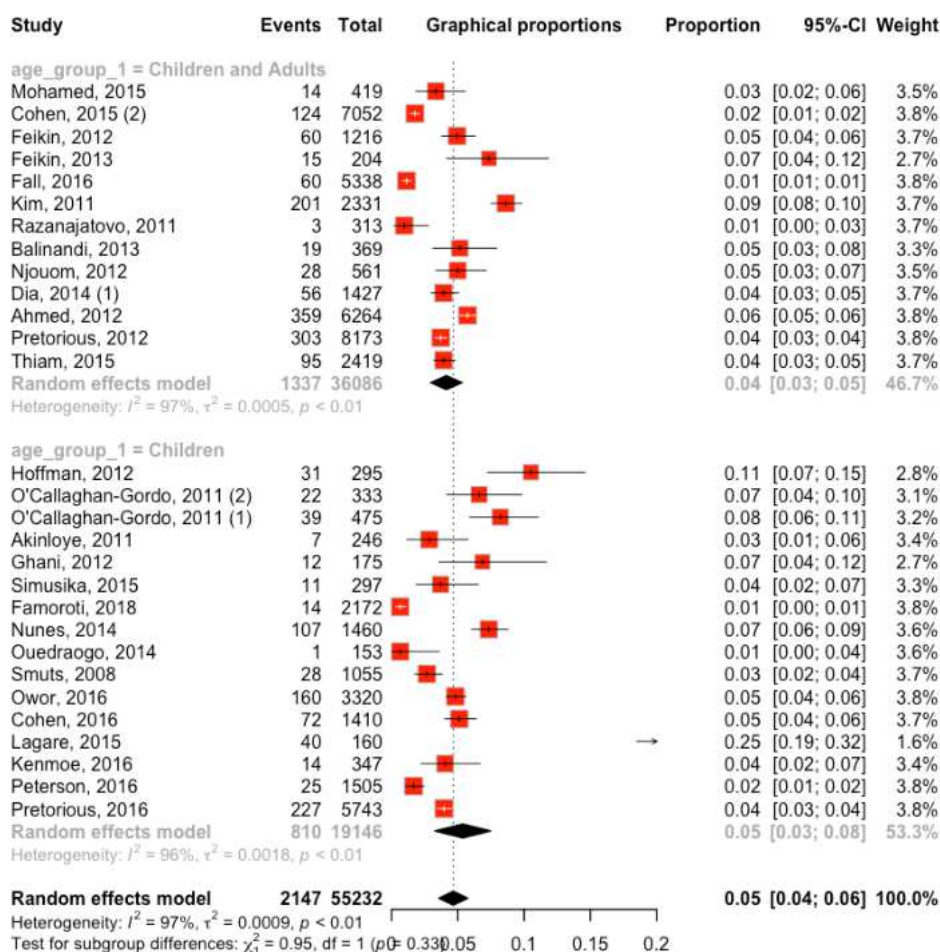

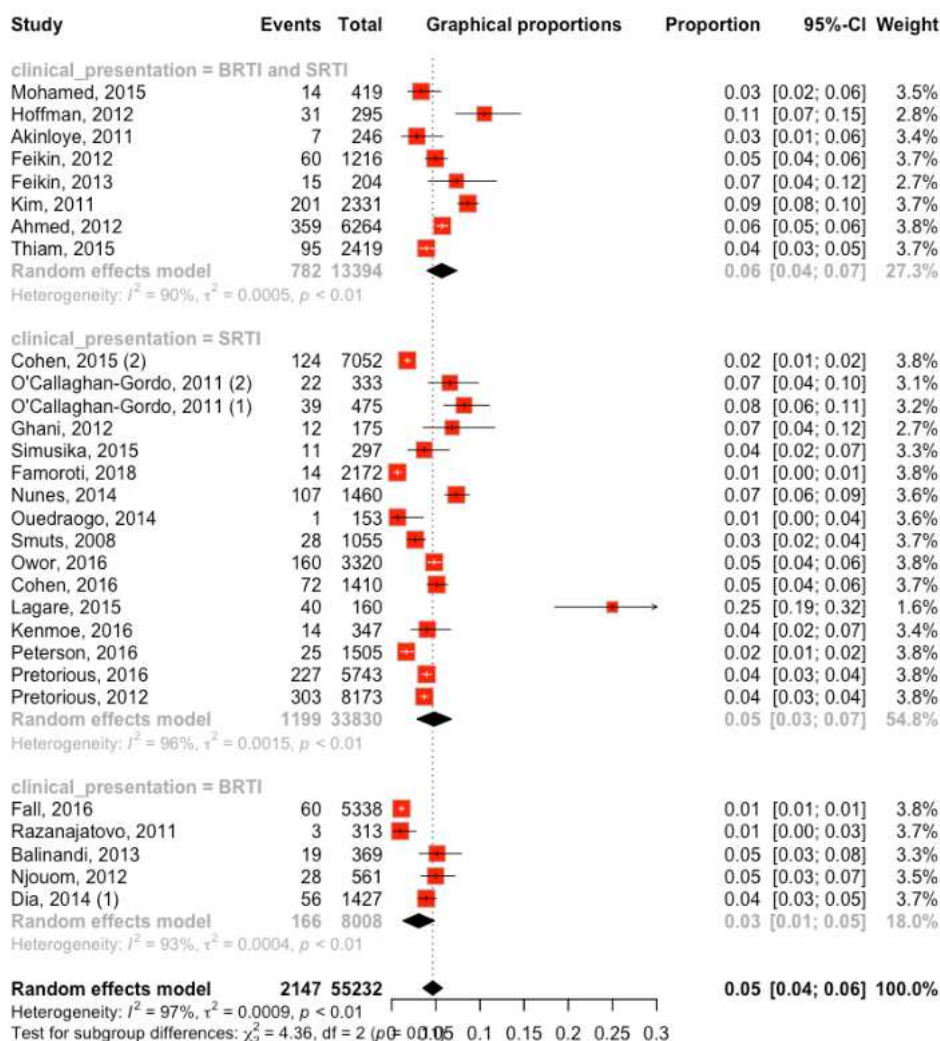

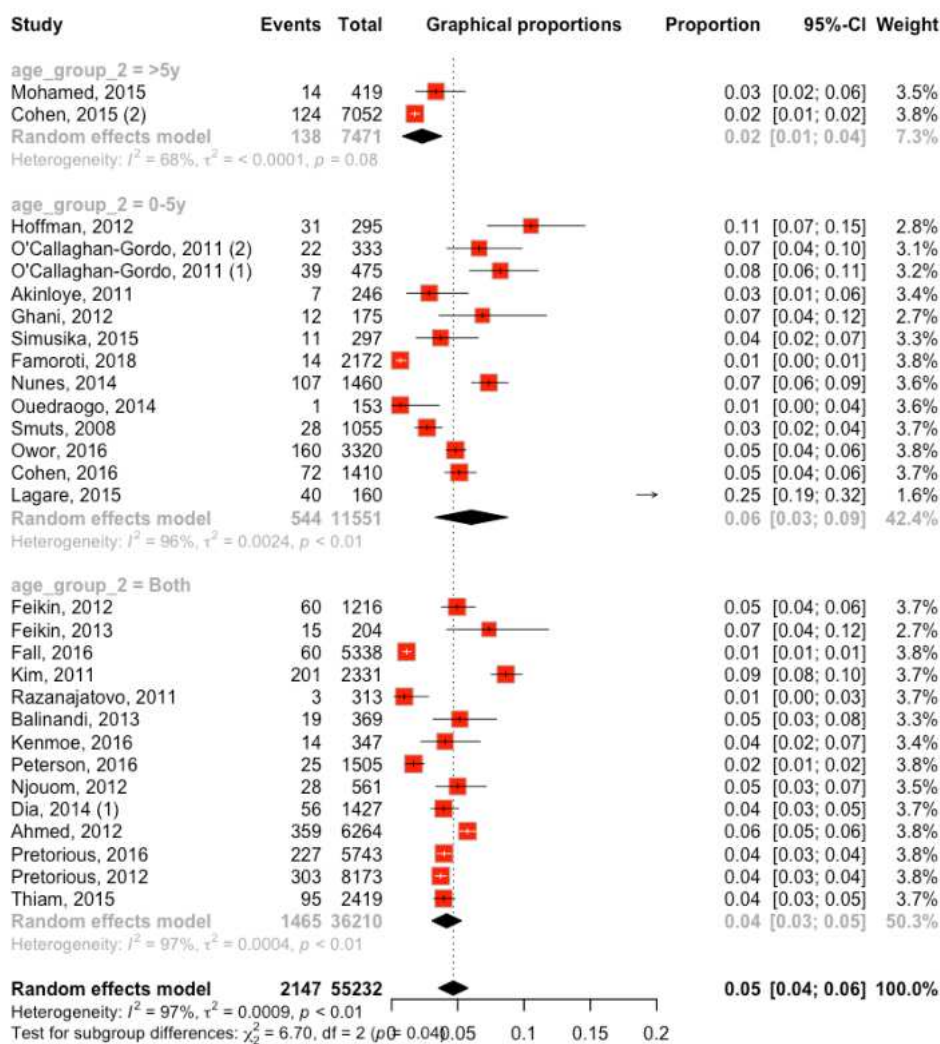

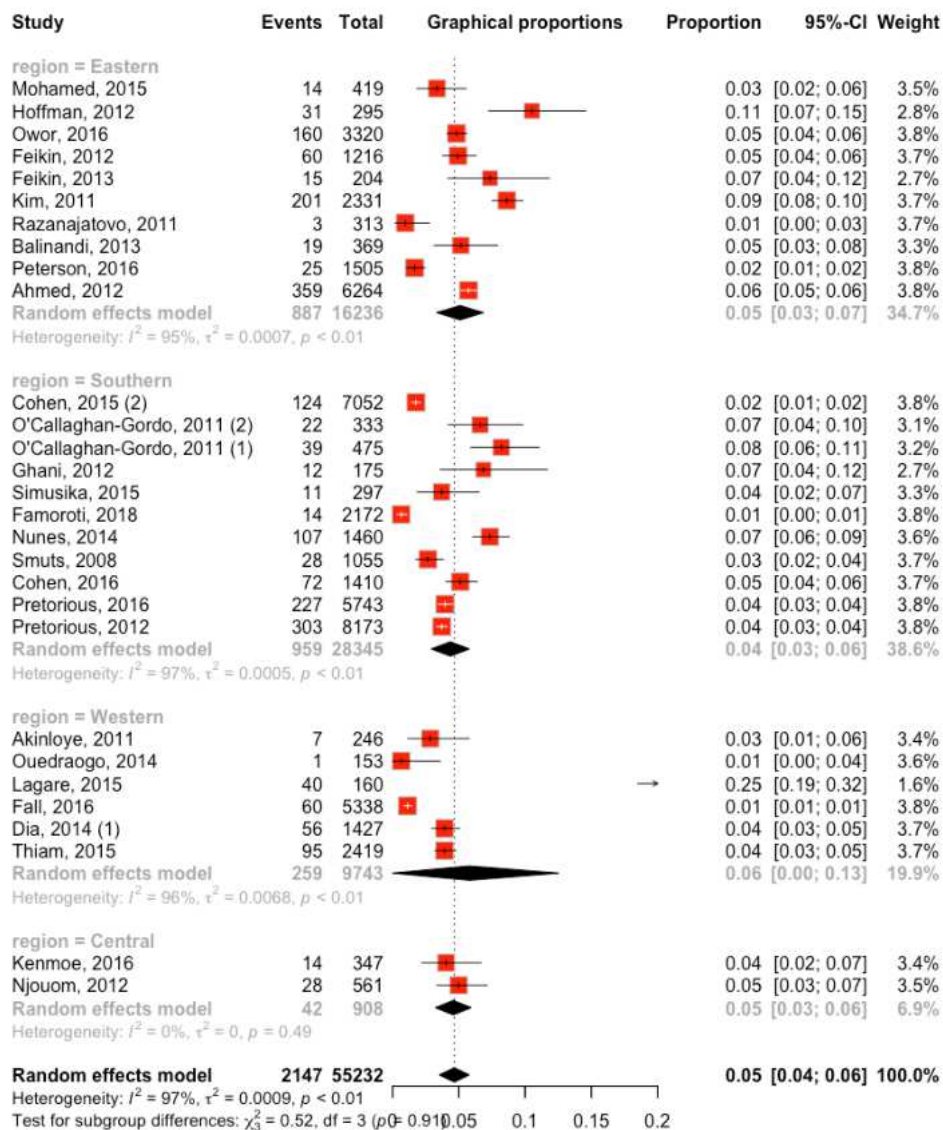

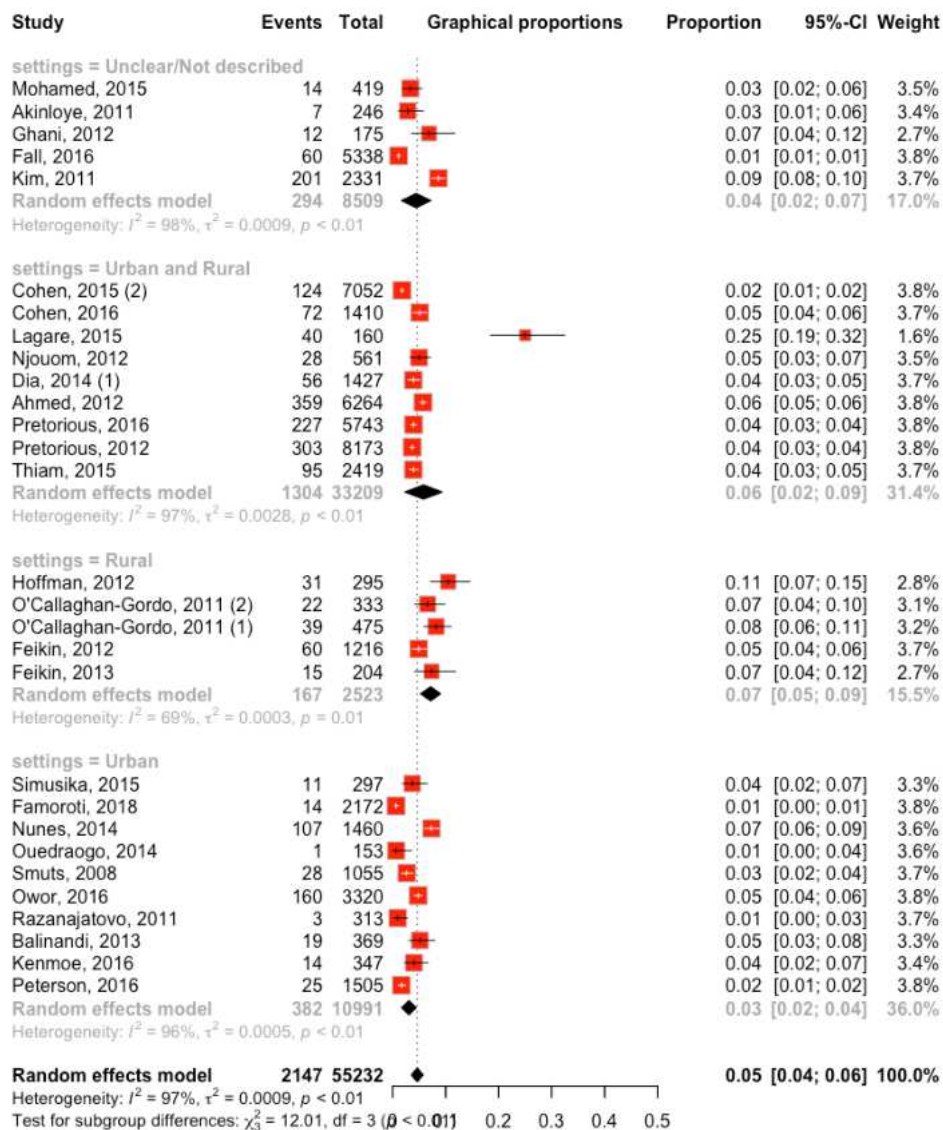

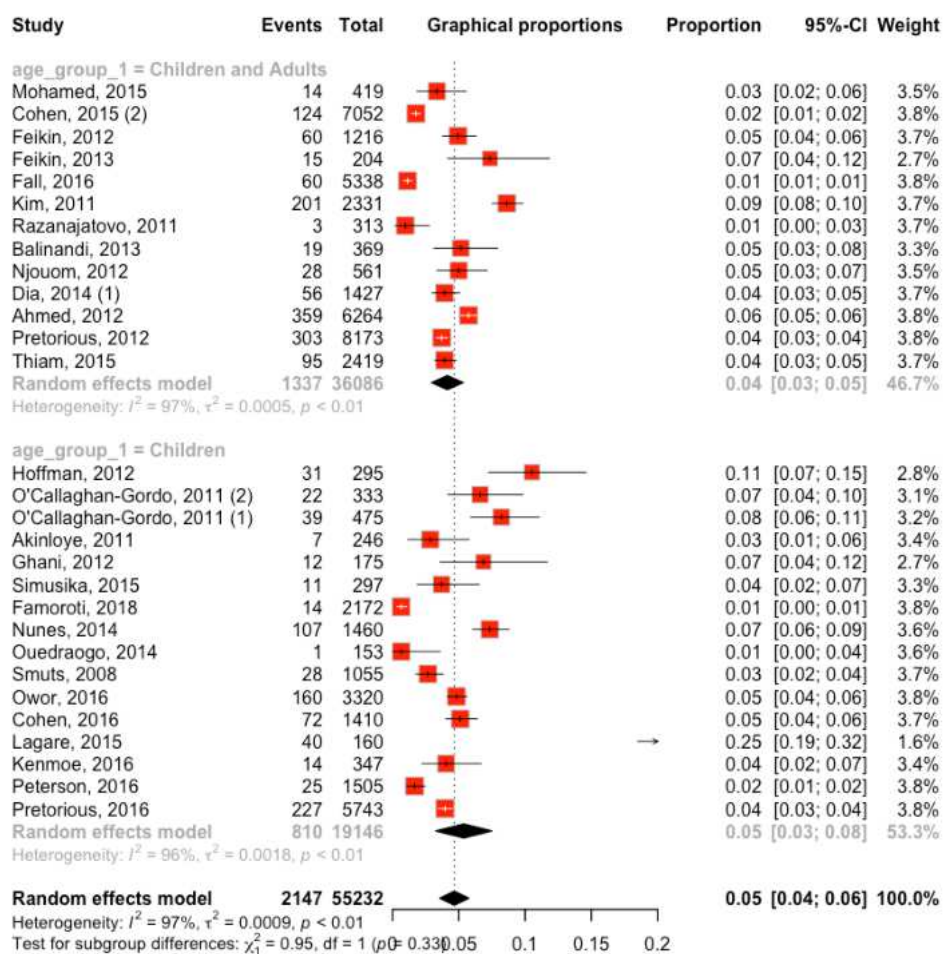

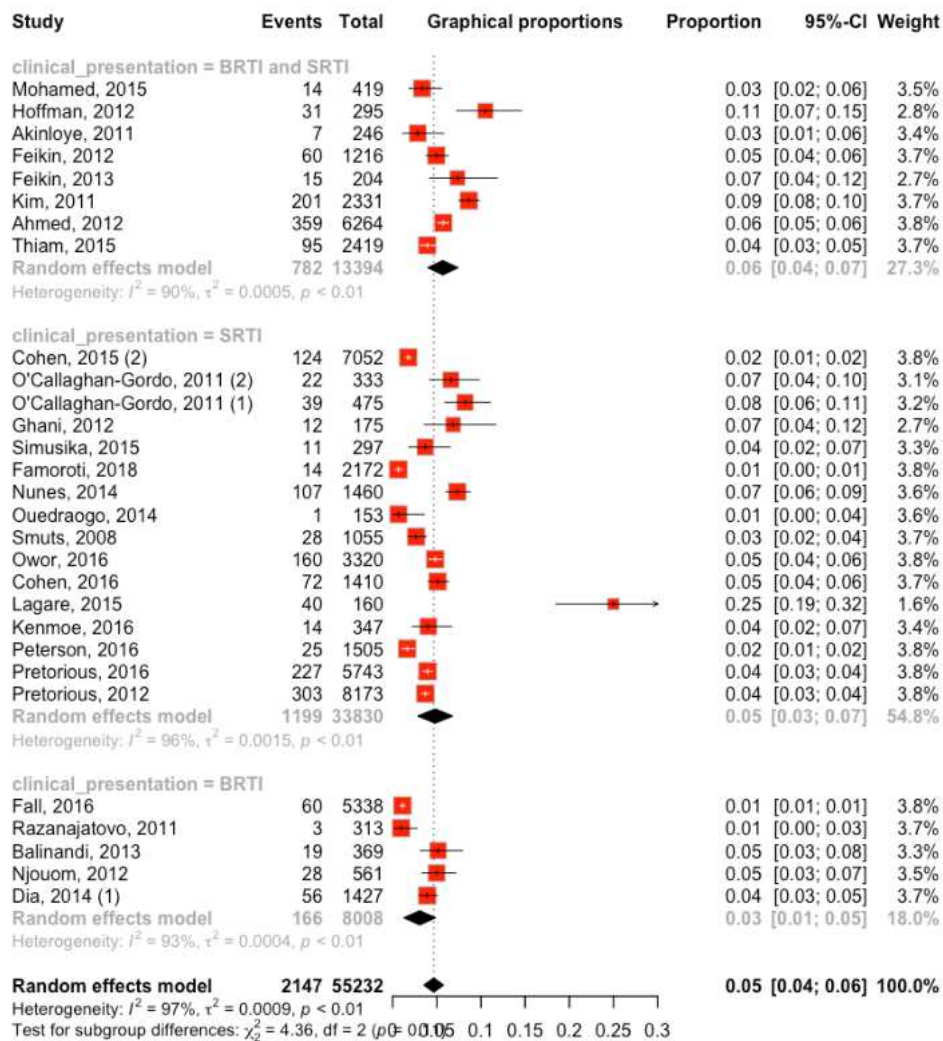

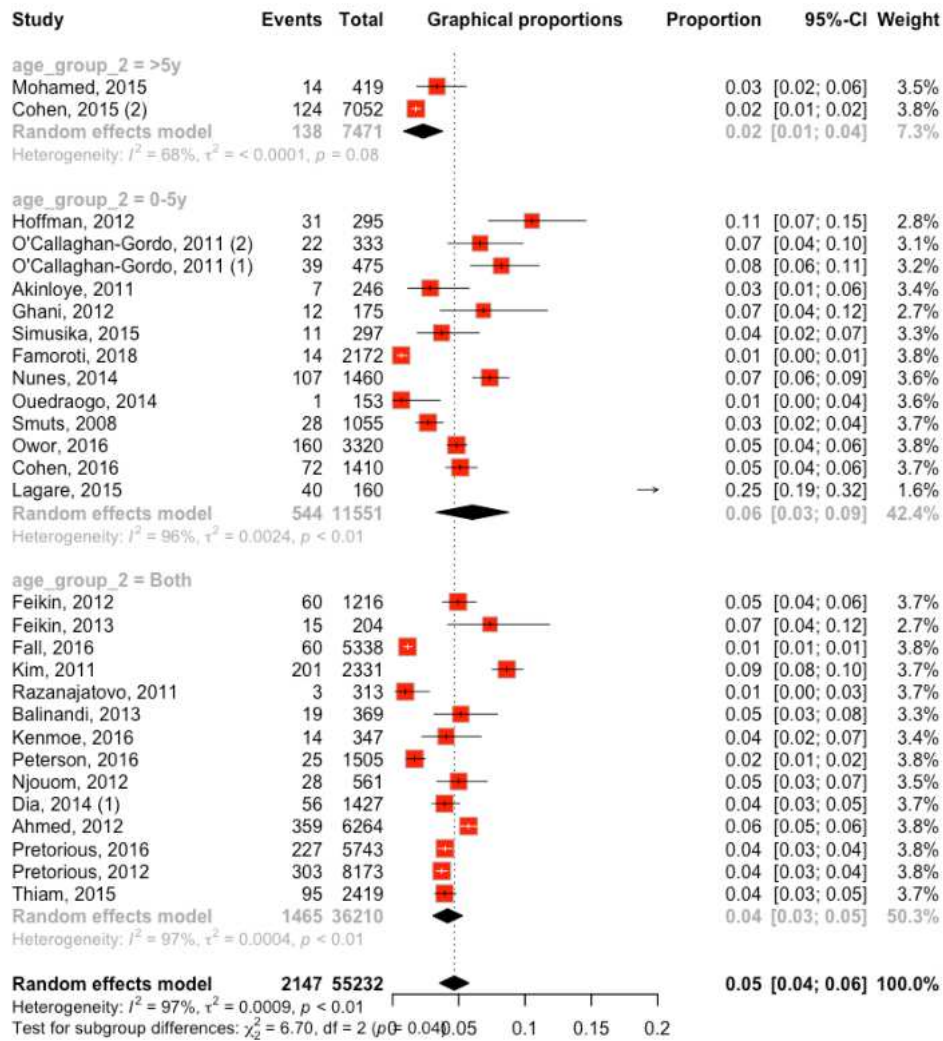

Supplement: Supplementary file 2 — Supplementary Material 2 [file 12879_2025_12122_MOESM2_ESM.zip › Fig.S4.pdf]

Fig. S5: Forest plot for HRV

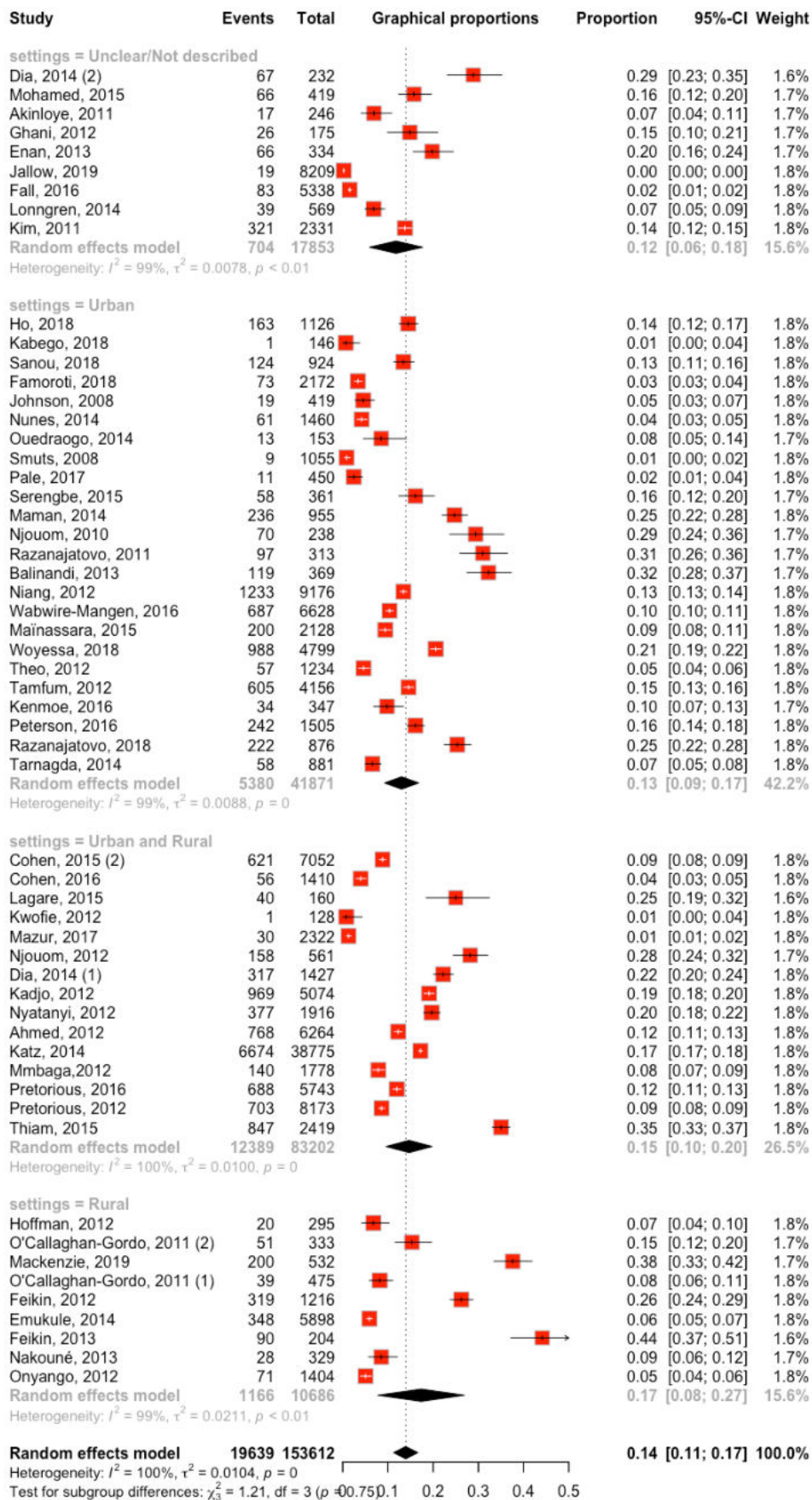

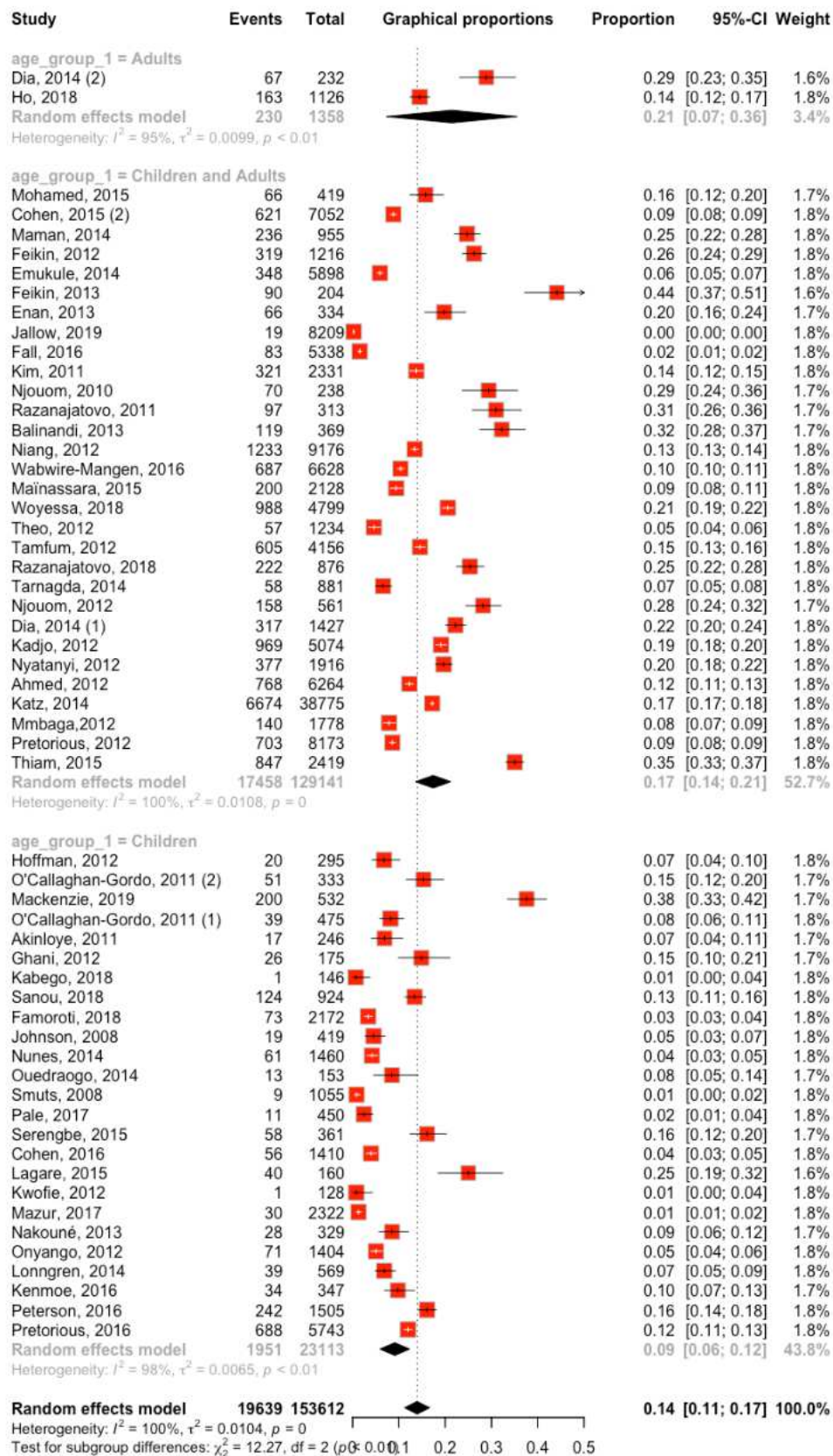

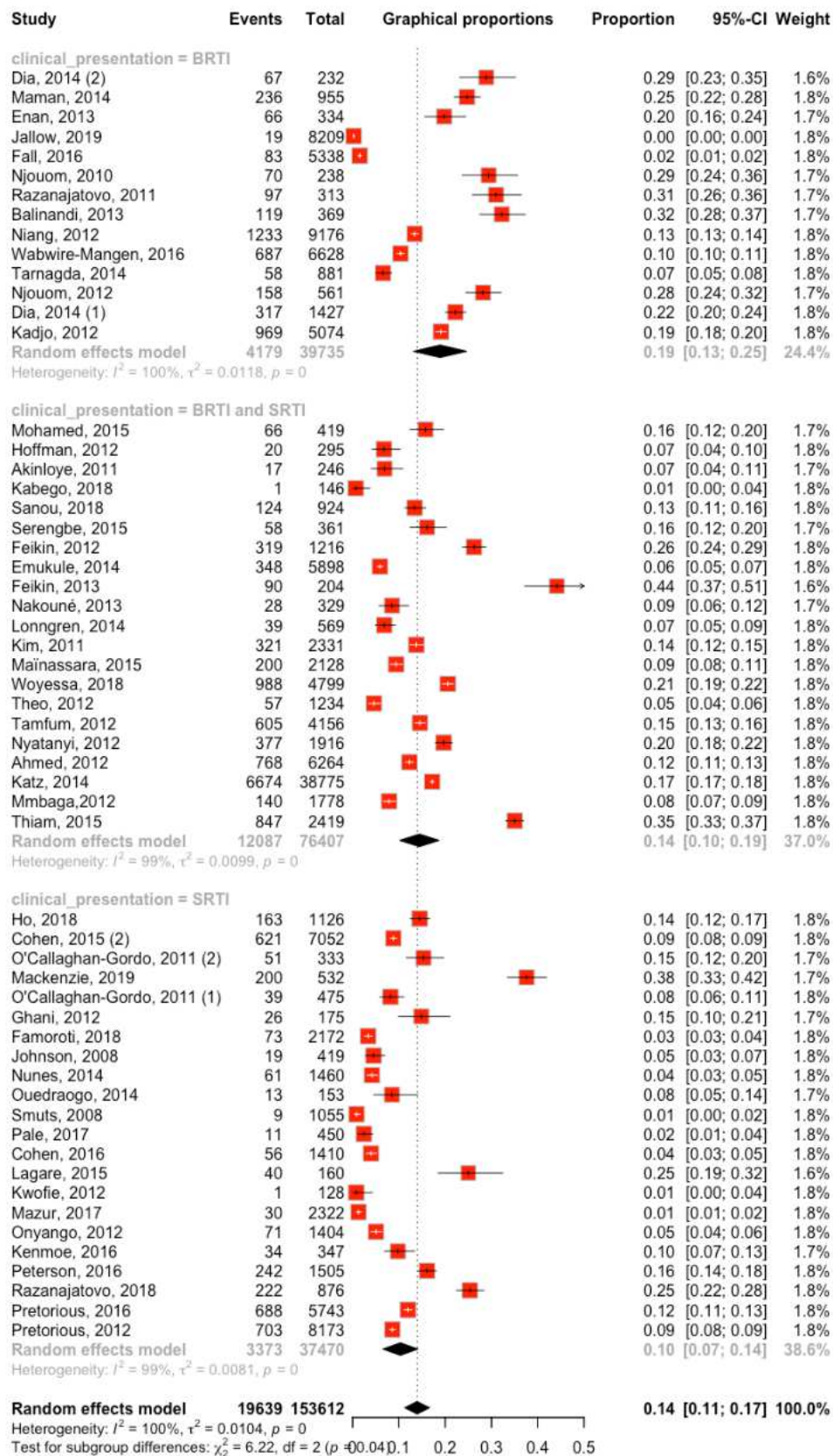

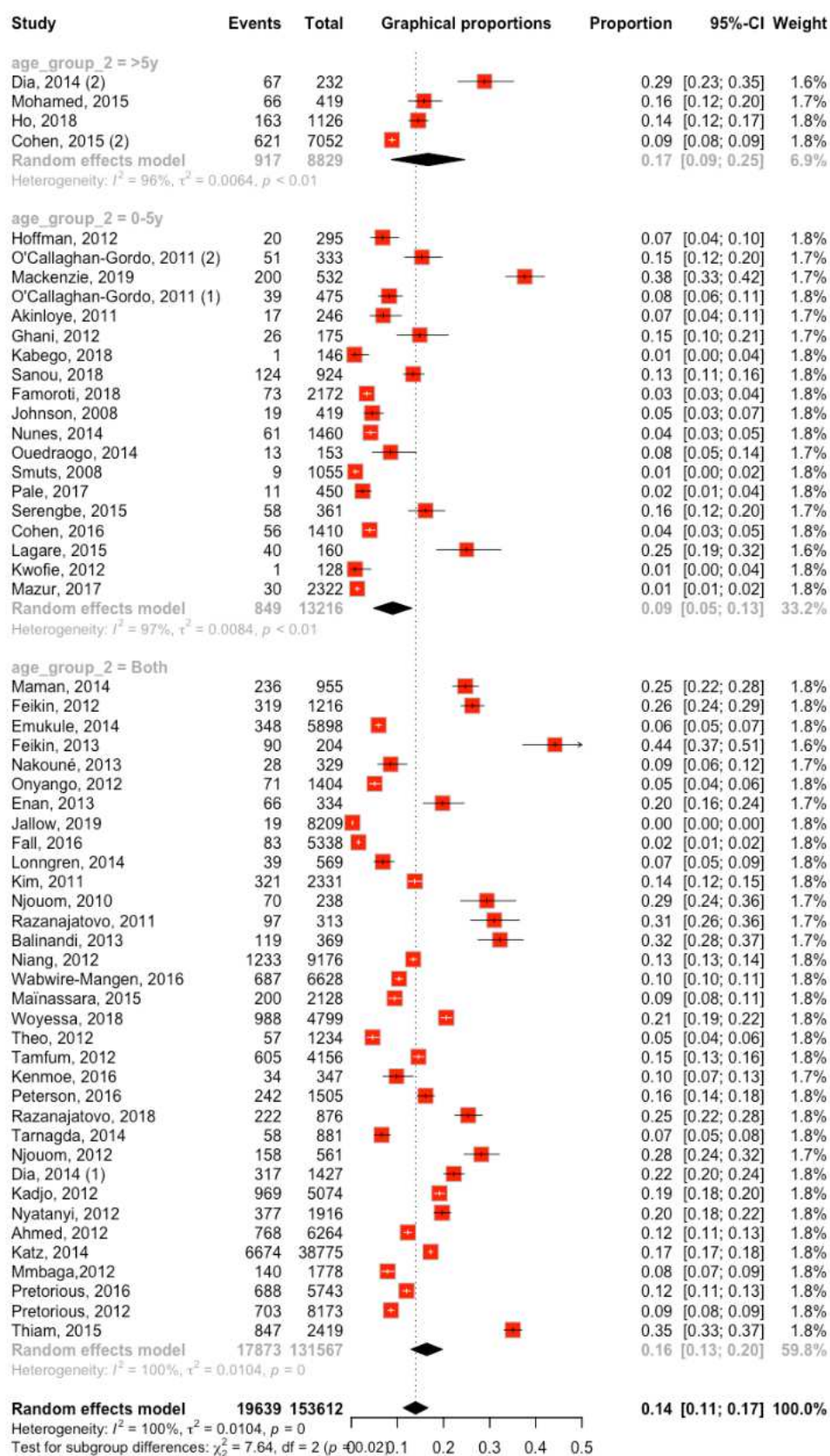

Supplement: Supplementary file 2 — Supplementary Material 2 [file 12879_2025_12122_MOESM2_ESM.zip › Fig.S5.pdf]

**Fig. S6: Forest plot for Enterovirus**

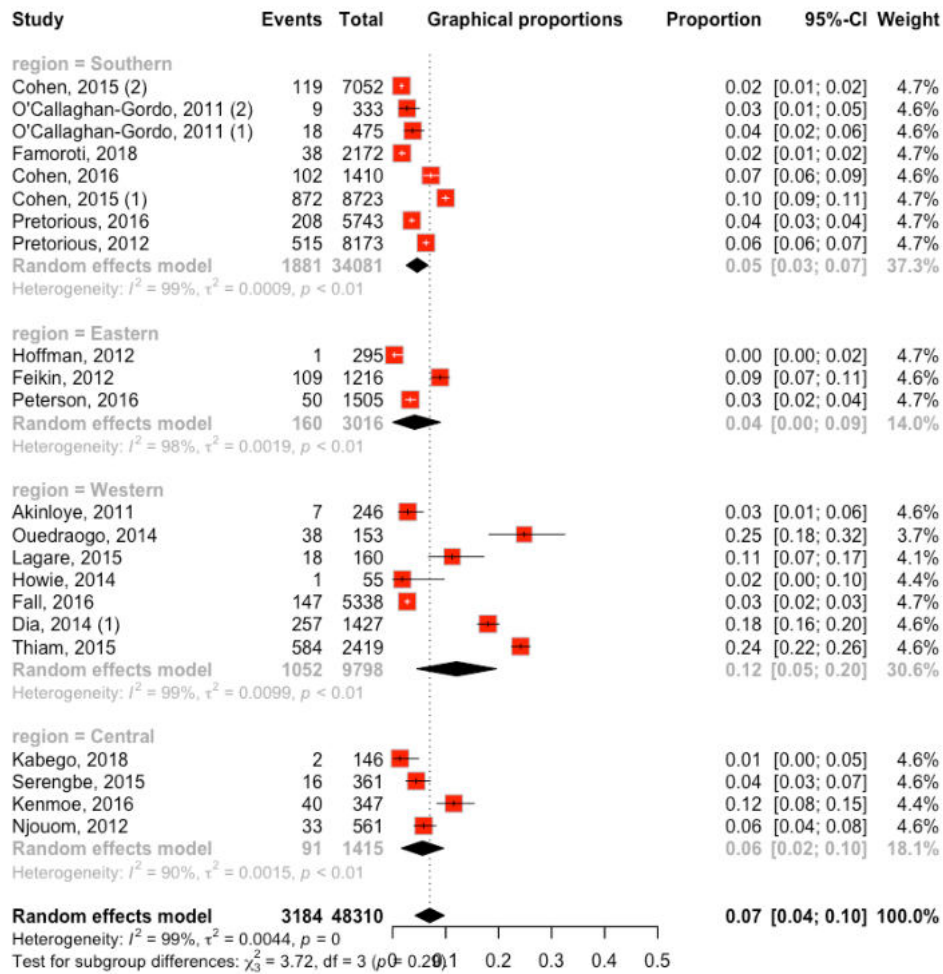

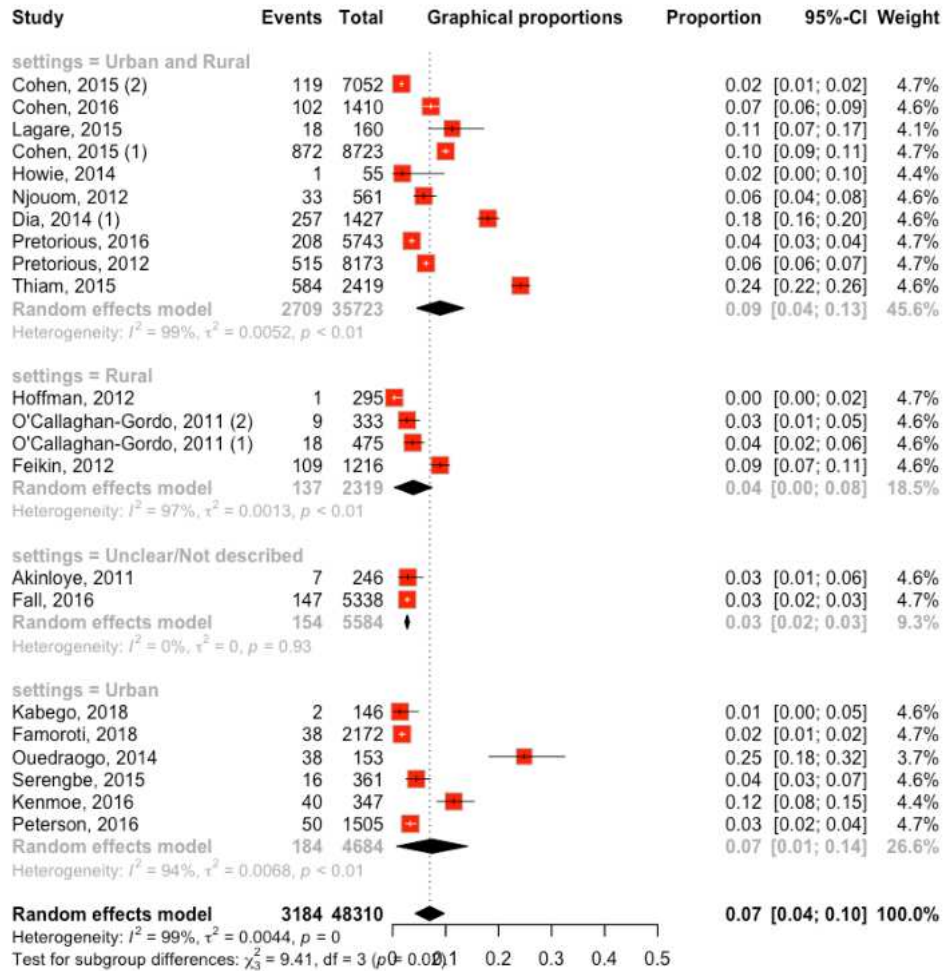

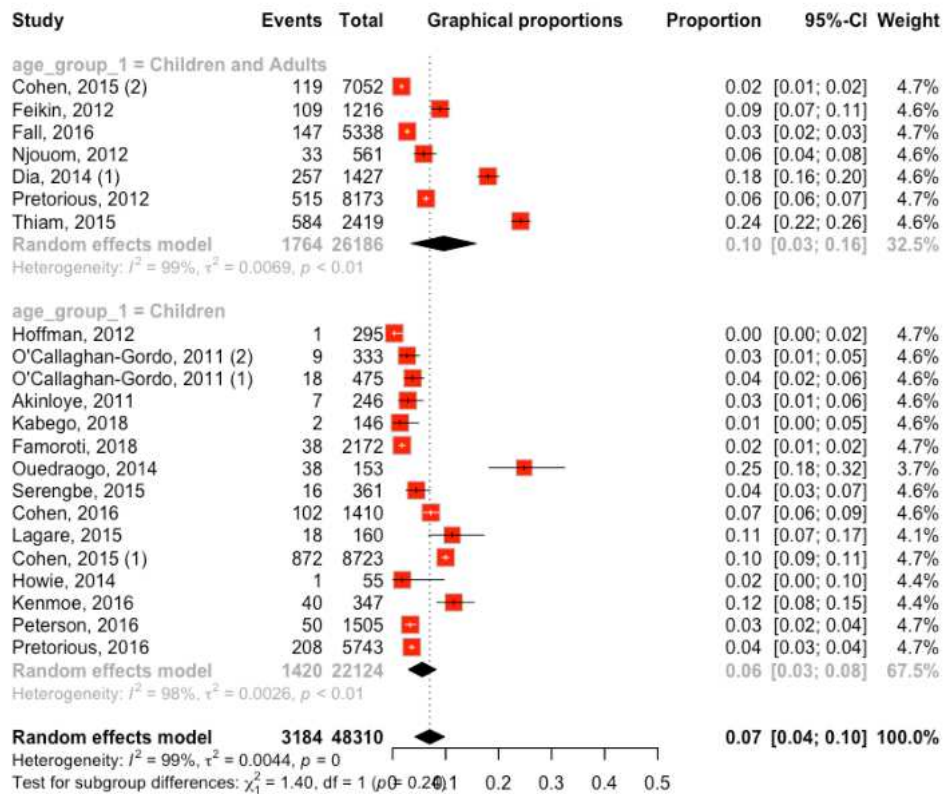

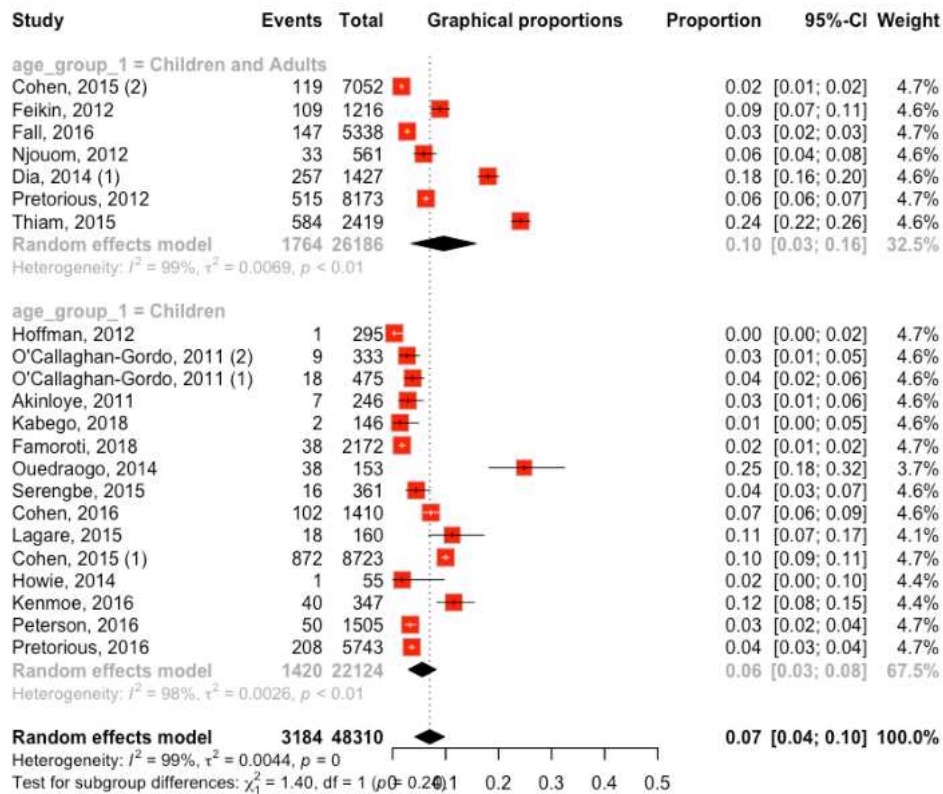

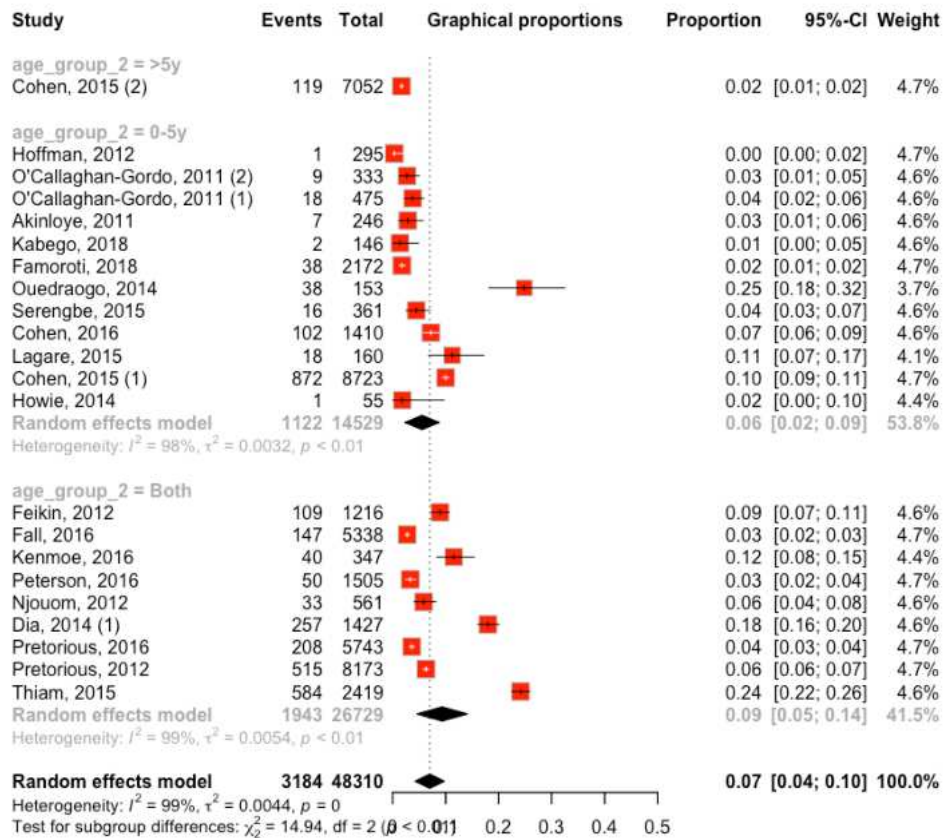

Supplement: Supplementary file 2 — Supplementary Material 2 [file 12879_2025_12122_MOESM2_ESM.zip › Fig.S6.pdf]

Fig. S7: Forest plot for Adv

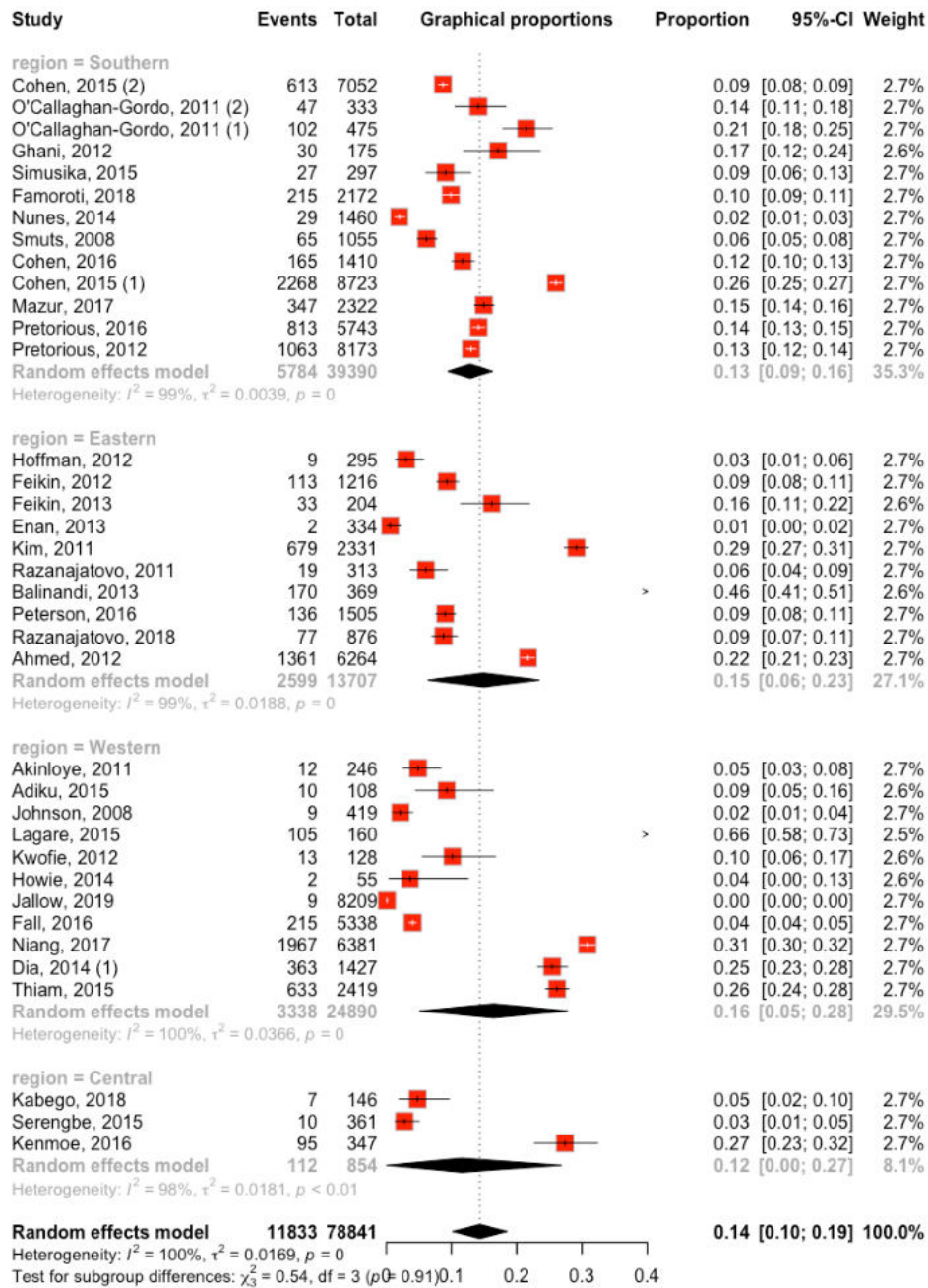

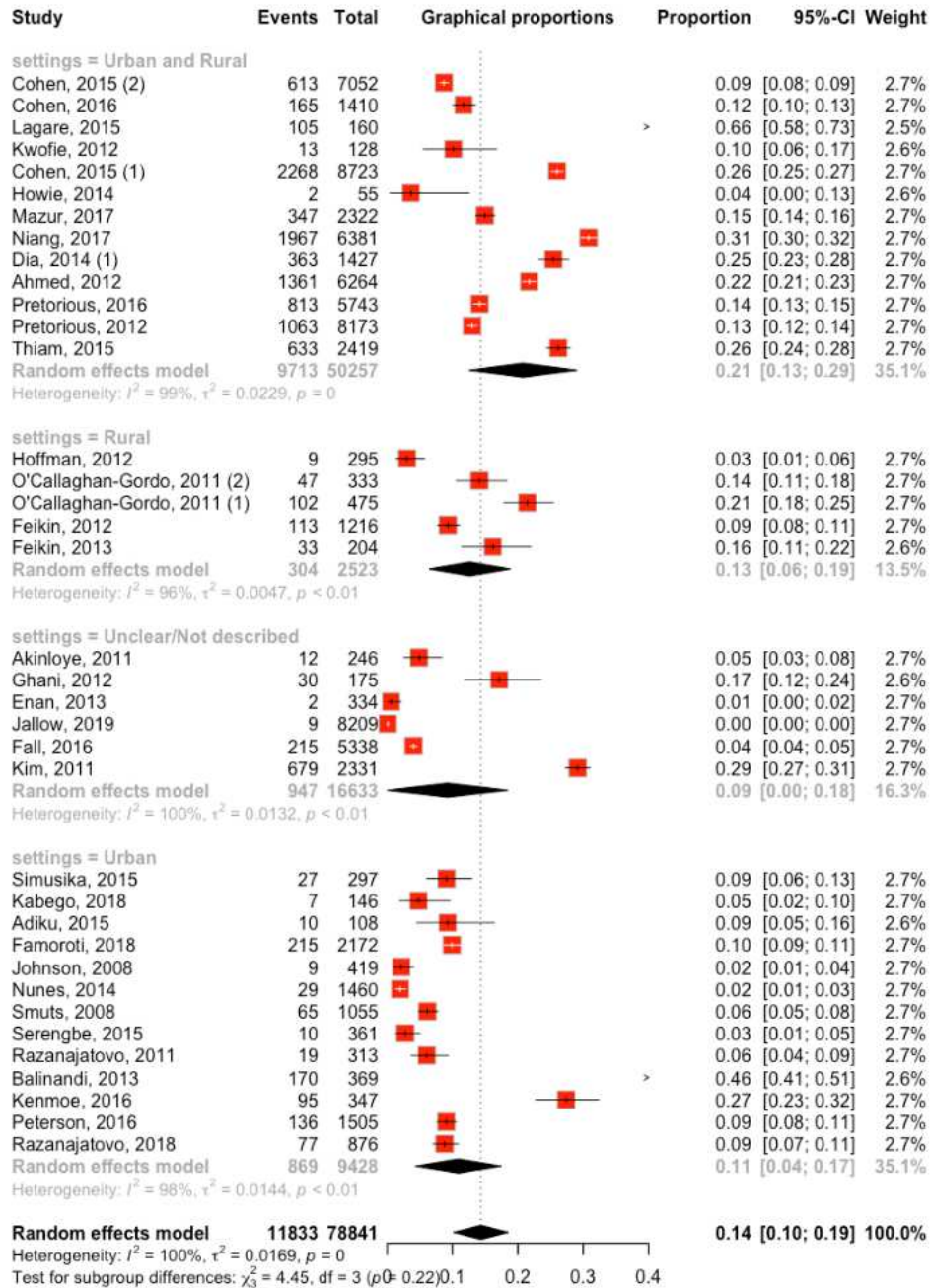

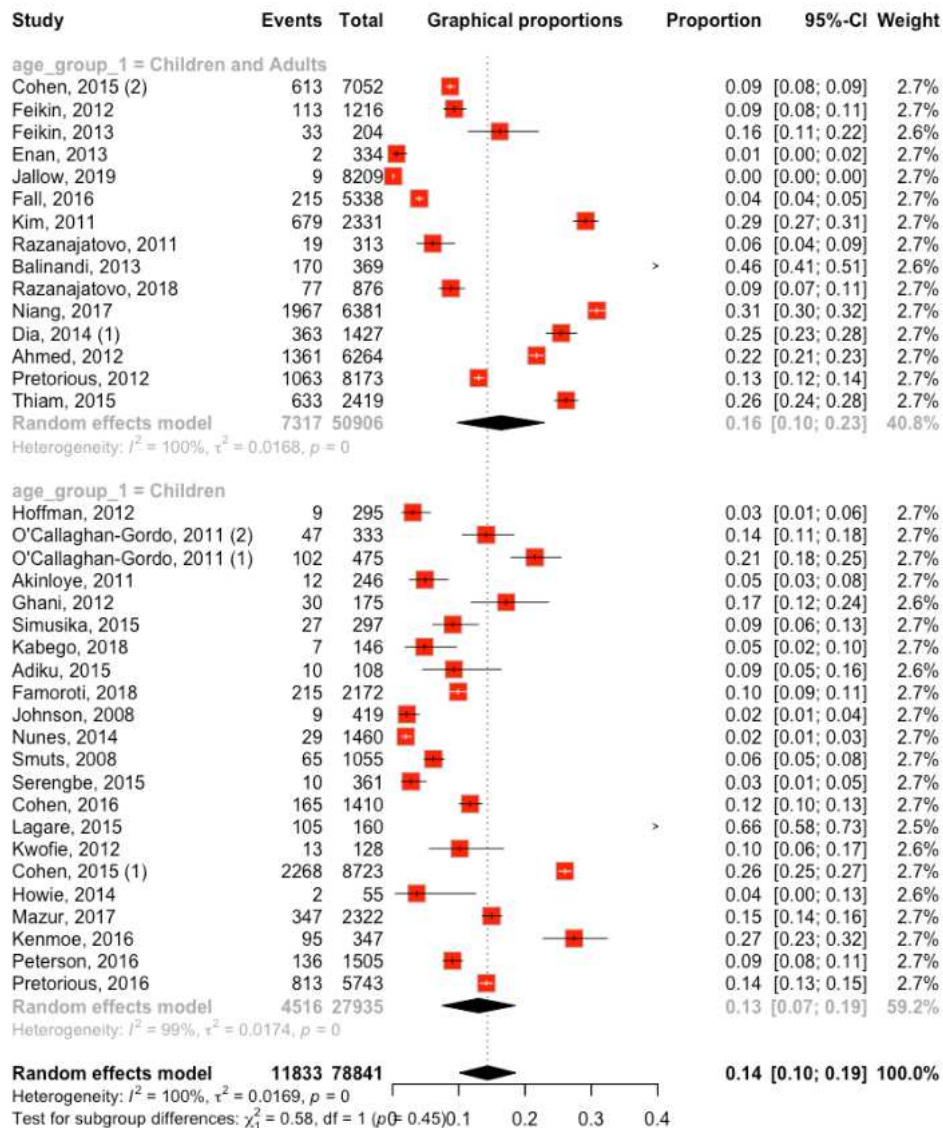

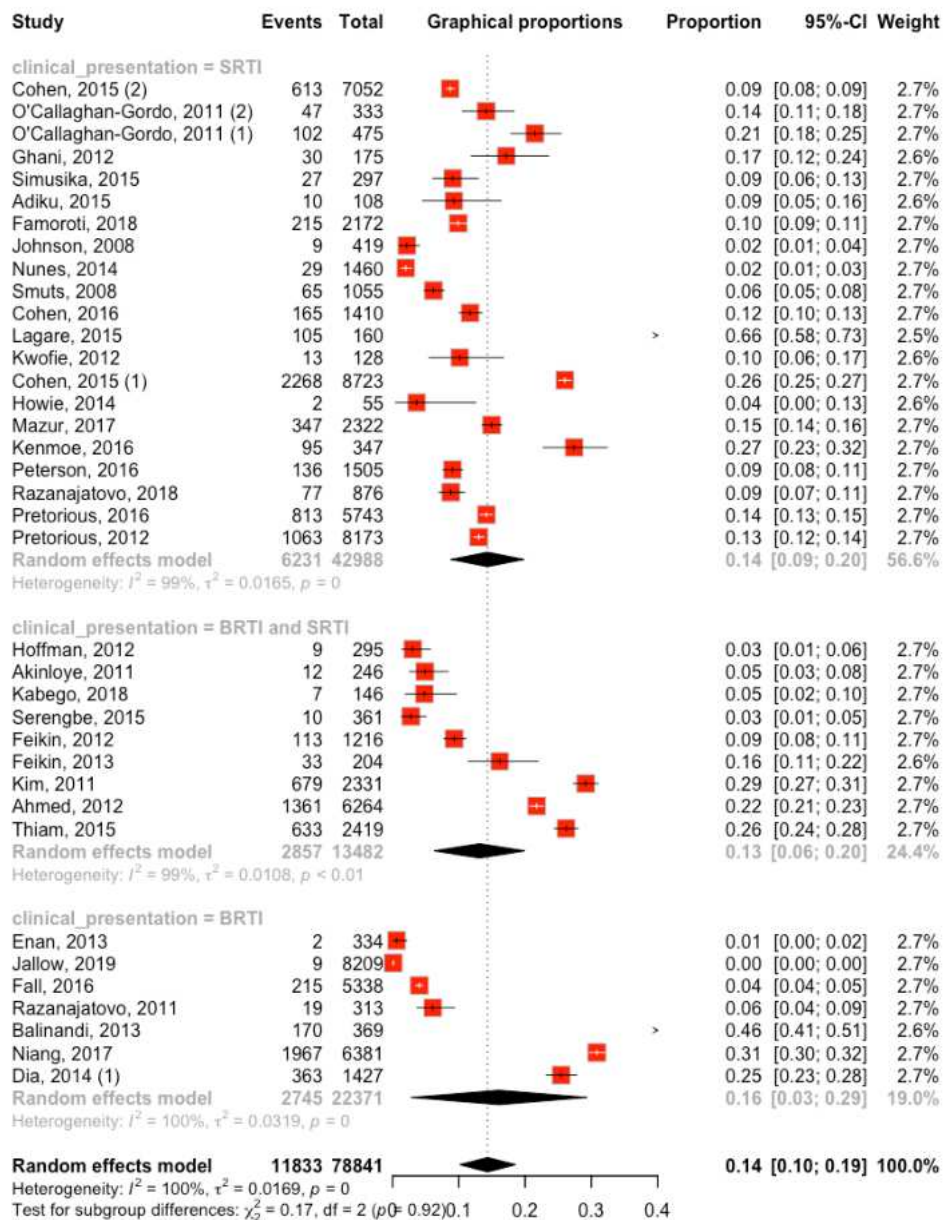

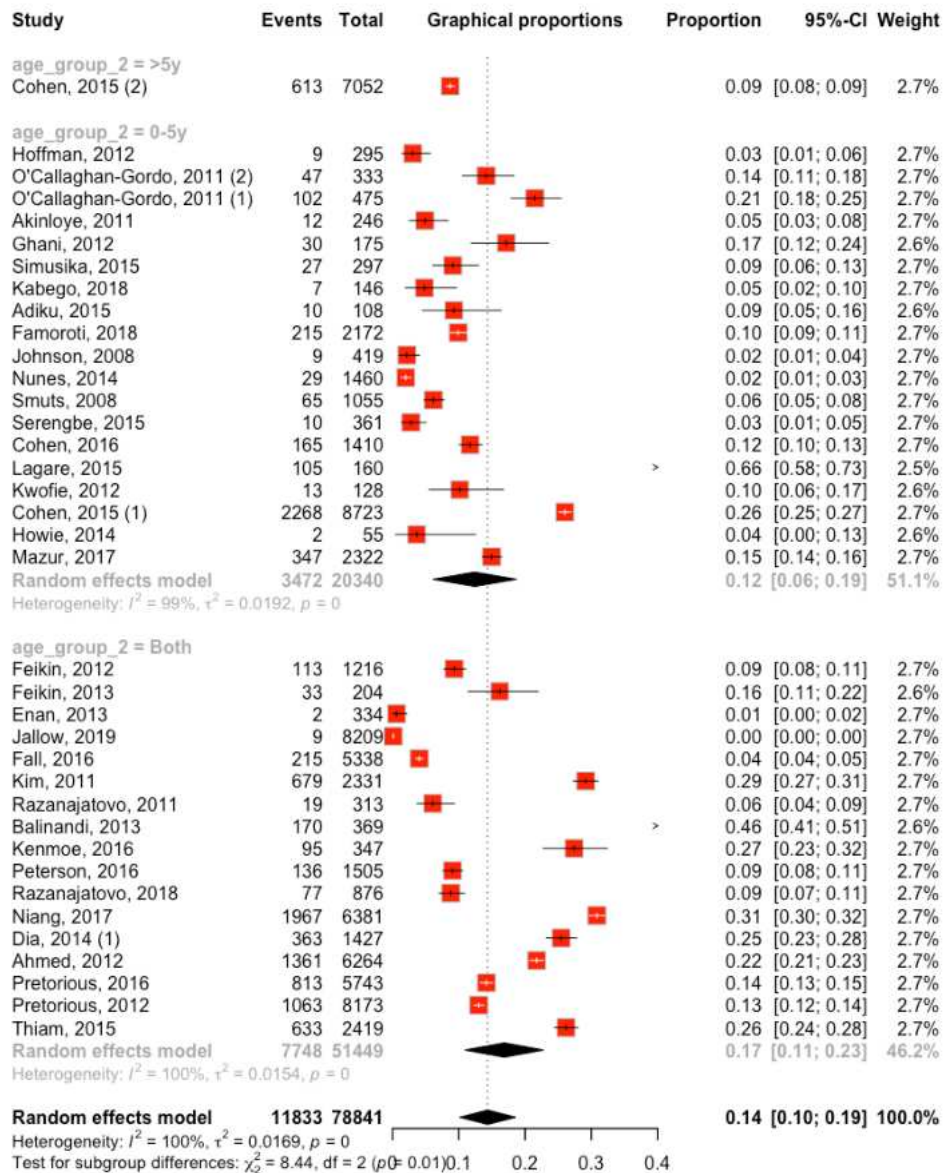

Supplement: Supplementary file 2 — Supplementary Material 2 [file 12879_2025_12122_MOESM2_ESM.zip › Fig.S7.pdf]

Fig. S8: Forest plot for HBoV

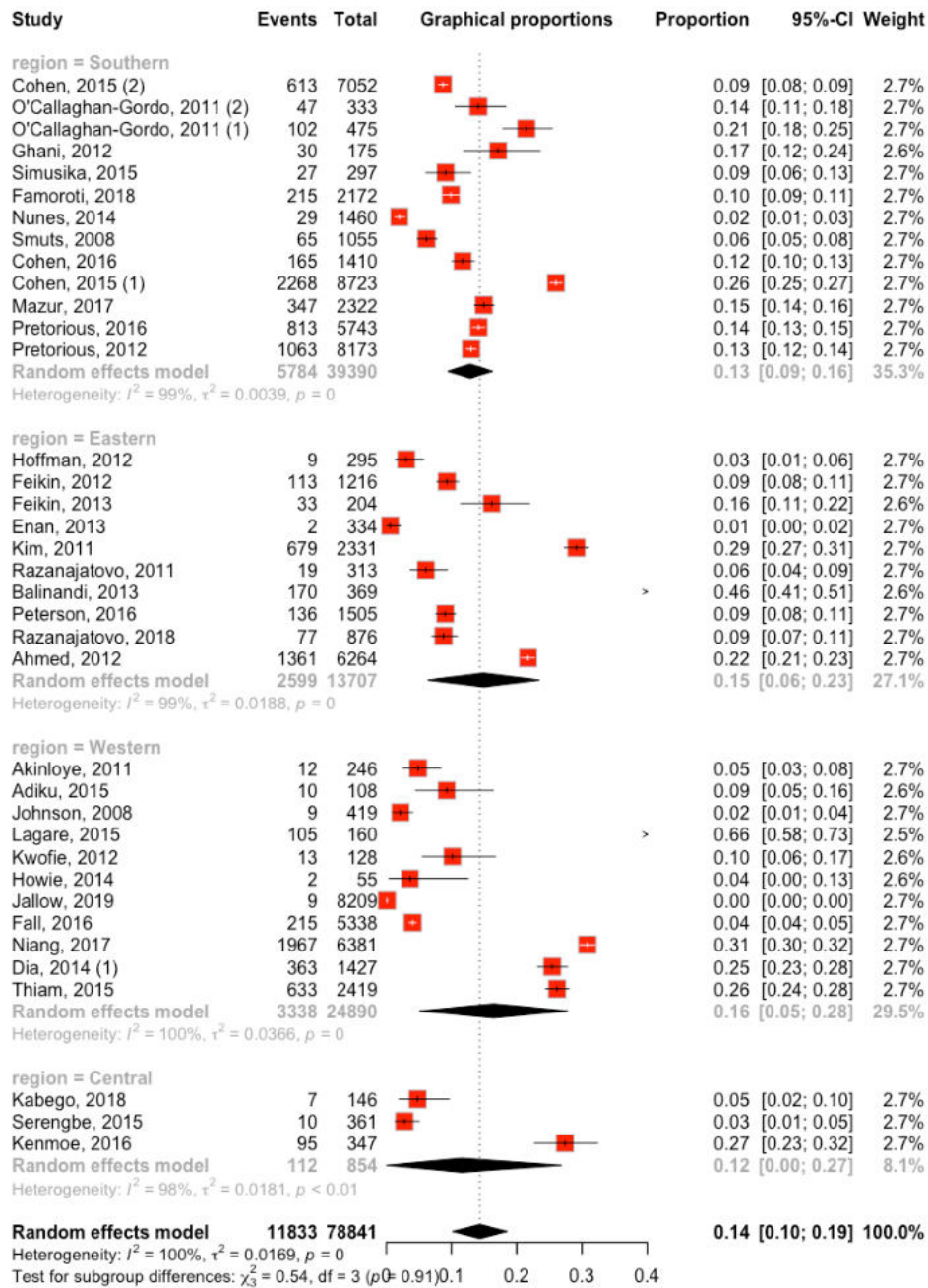

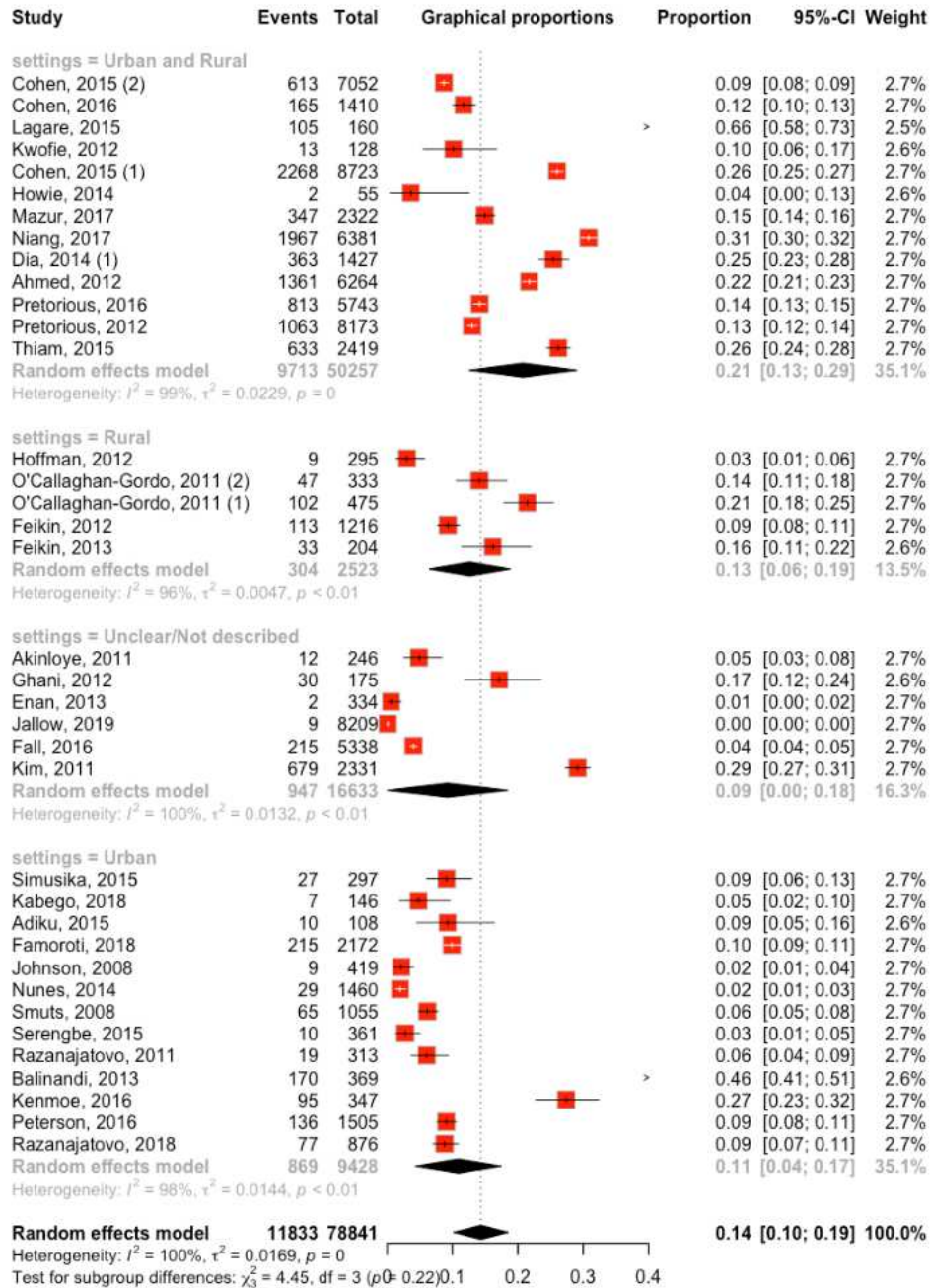

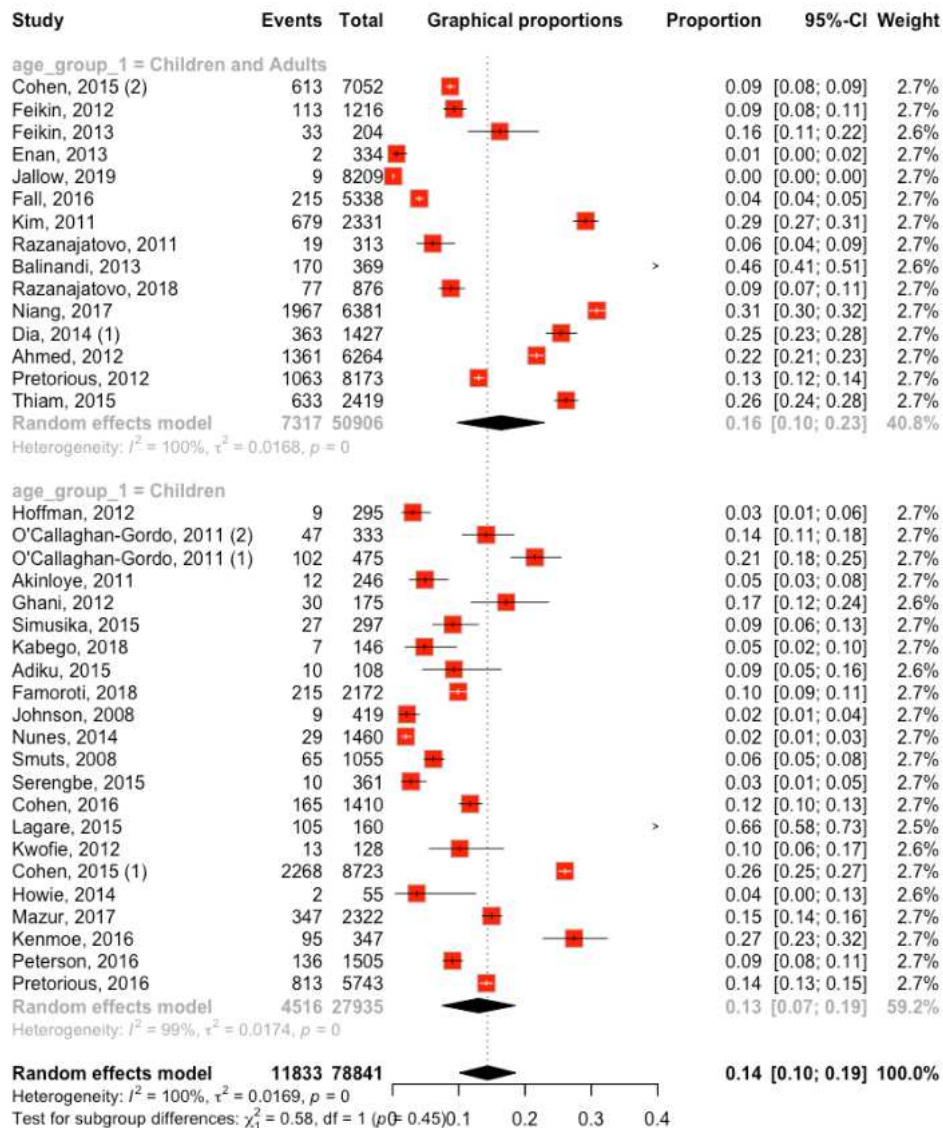

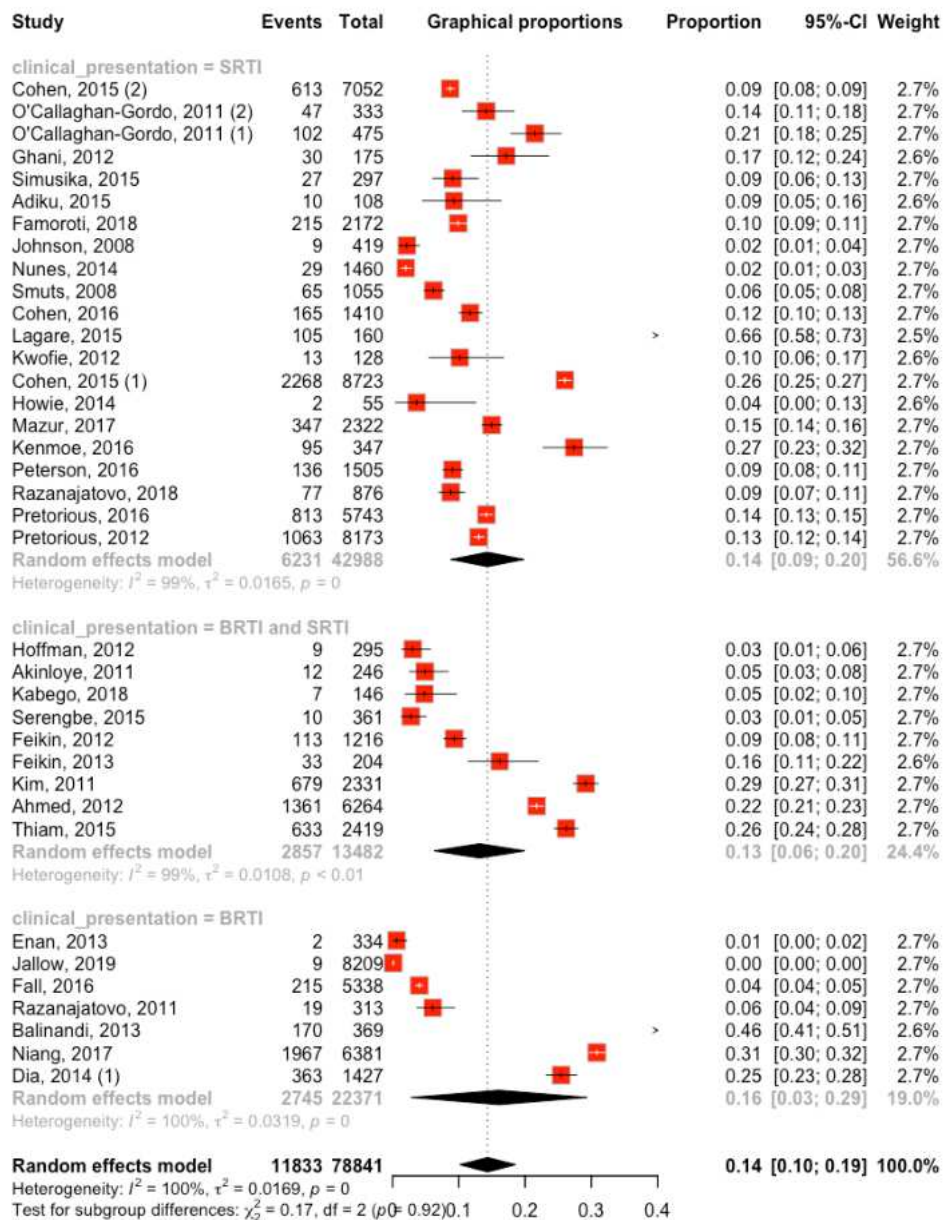

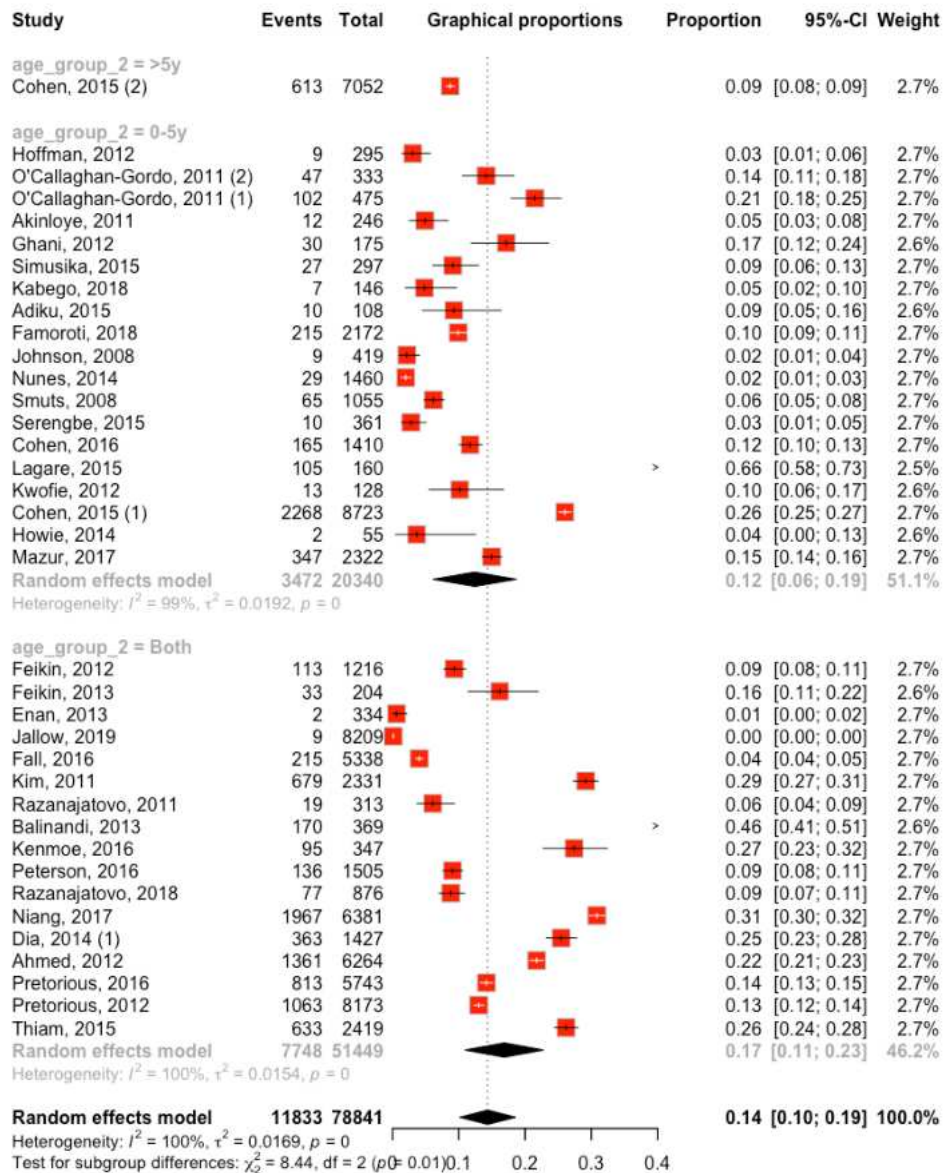

Supplement: Supplementary file 2 — Supplementary Material 2 [file 12879_2025_12122_MOESM2_ESM.zip › Fig.S8.pdf]

Fig. S9: Forest plot for HCoV

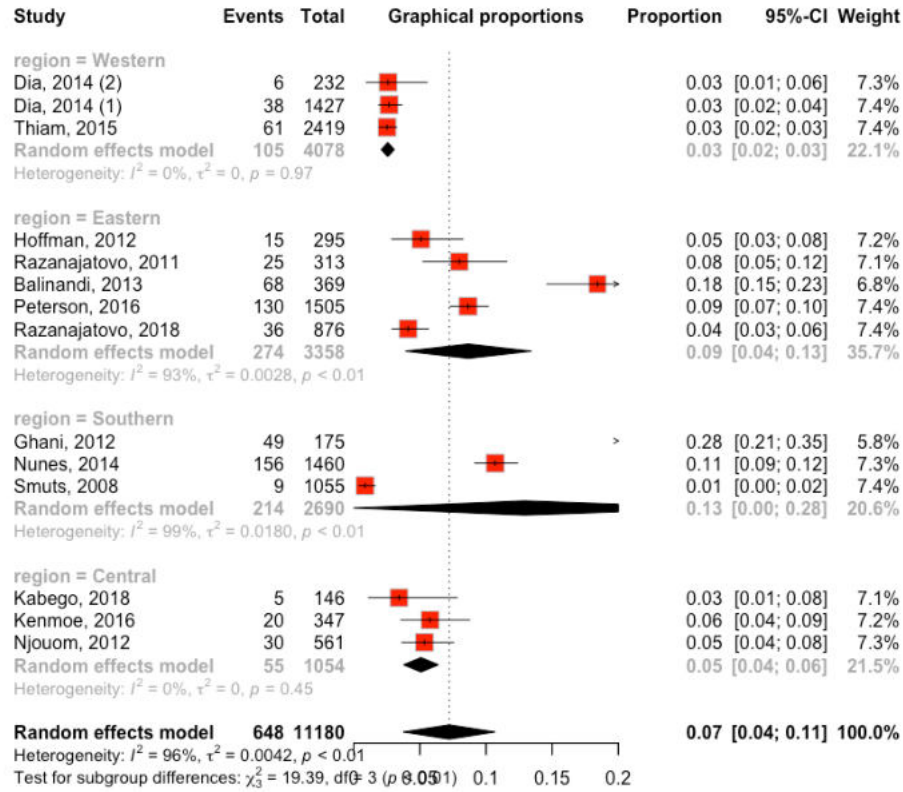

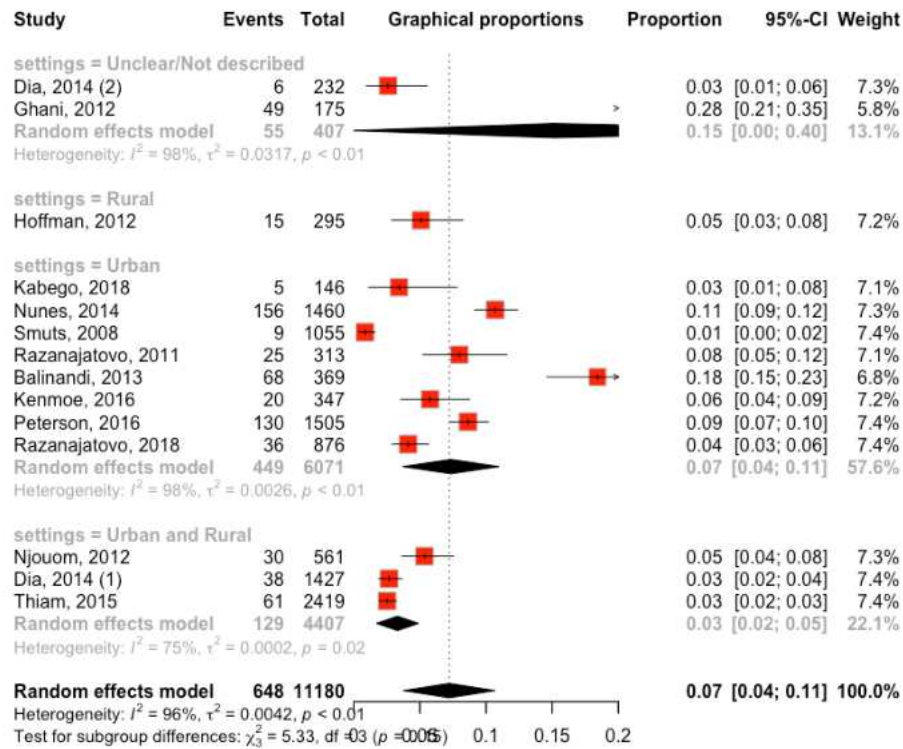

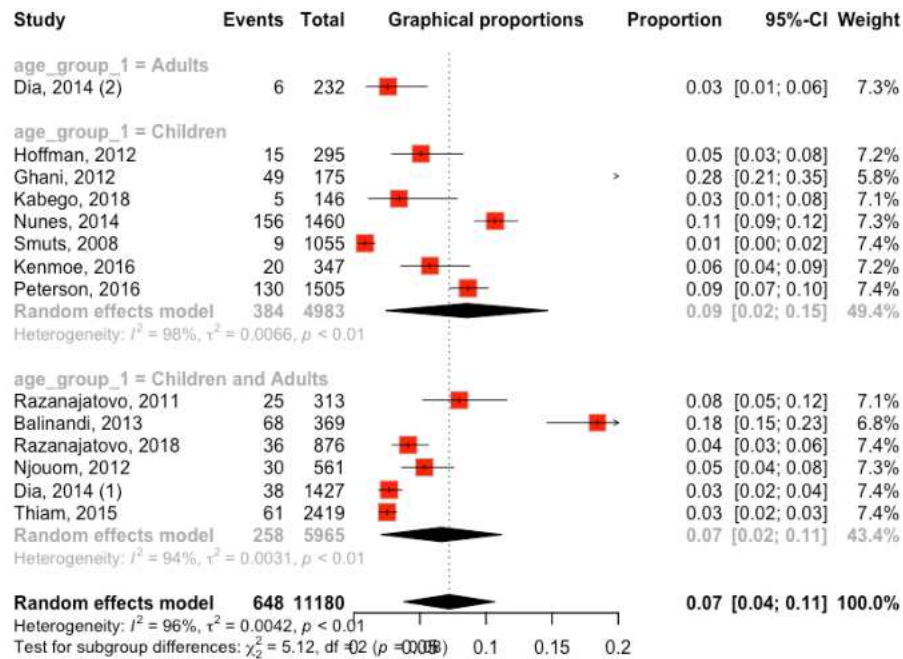

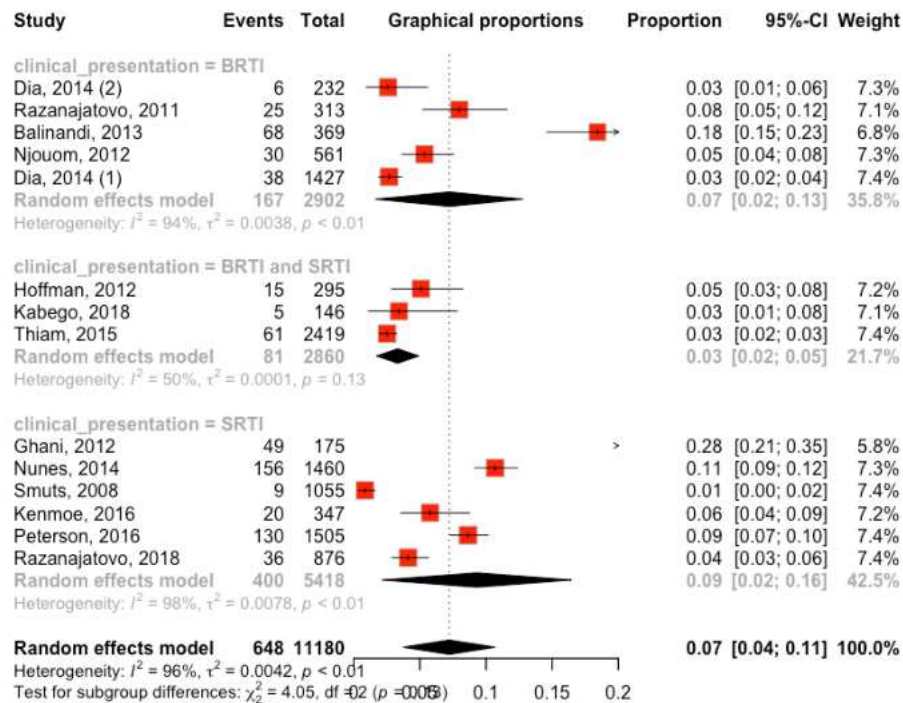

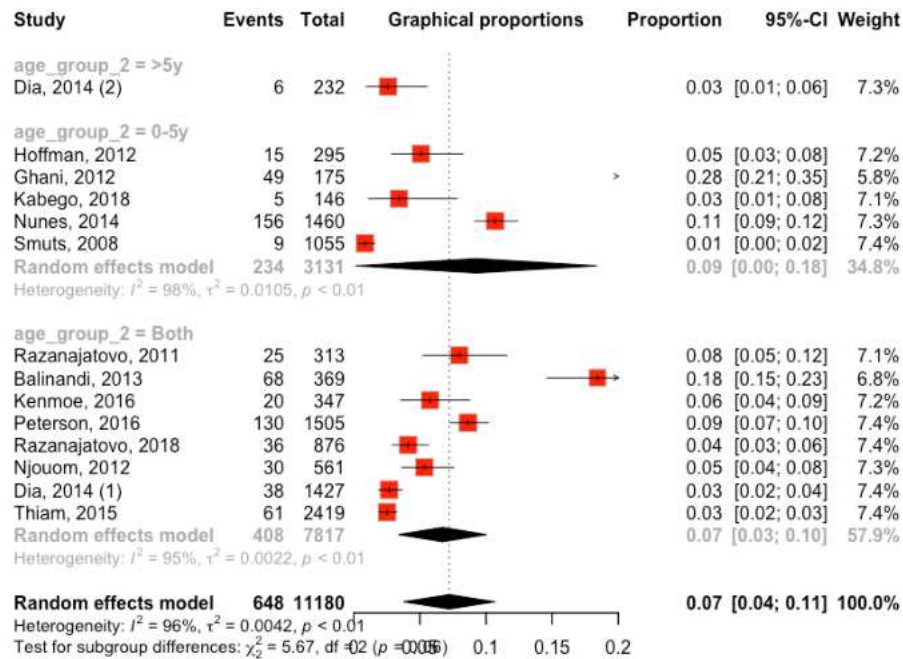

Supplement: Supplementary file 2 — Supplementary Material 2 [file 12879_2025_12122_MOESM2_ESM.zip › Fig.S9.pdf]
